# Dual E3 ligase recruitment by monovalent degraders for tunable SMARCA 2/4 degradation

**DOI:** 10.1038/s41589-026-02224-y

**Published:** 2026-05-12

**Authors:** Valentina A. Spiteri, Dmitri Segal, Alejandro Correa-Sáez, Kentaro Iso, Ryan Casement, Miquel Muñoz i Ordoño, Mark A. Nakasone, Gajanan Sathe, Caroline Schätz, Hannah E. Peters, Mark Doward, Lisa Kainacher, Angus D. Cowan, Alessio Ciulli, Georg E. Winter

**Affiliations:** 1 Centre for Targeted Protein Degradation, School of Life Sciences, University of Dundee, Dundee, UK; 2 CeMM Research Center for Molecular Medicine of the Austrian Academy of Sciences, Vienna, Austria; 3 AITHYRA Research Institute for Biomedical Artificial Intelligence of the Austrian Academy of Sciences, Vienna, Austria

**Keywords:** Mechanism of action, Chemical genetics, Target identification

## Abstract

Proteolysis-targeting chimeras (PROTACs) and molecular glue degraders (MGDs) target proteins for degradation by co-opting an E3 ligase. While heterotrivalent PROTACs that can recruit multiple E3 ligases have been described, all MGDs reported to date depend on a single E3. Using orthogonal genetic screening, biophysical and structural analyses, we show that a monovalent MGD can recruit CUL4^DCAF16^ and CRL1^FBXO22^ in parallel to degrade SMARCA2/4. Deep mutational scanning identifies C173 in DCAF16 as essential for degrader activity and intact protein mass spectrometry confirms covalent modification at this site. Elucidating the ternary complex structure reveals a unique binding mode and a distinct interface of neointeractions that underlie degrader specificity. We demonstrate that ligase dependency is chemically and genetically tunable. Minimal compound modifications shift preference from DCAF16 to FBXO22, while a single substitution boosts degrader dependency on DCAF16. These results establish a framework for designing tunable dual E3 ligase degraders to mitigate potential resistance mechanisms.

**Figure:**
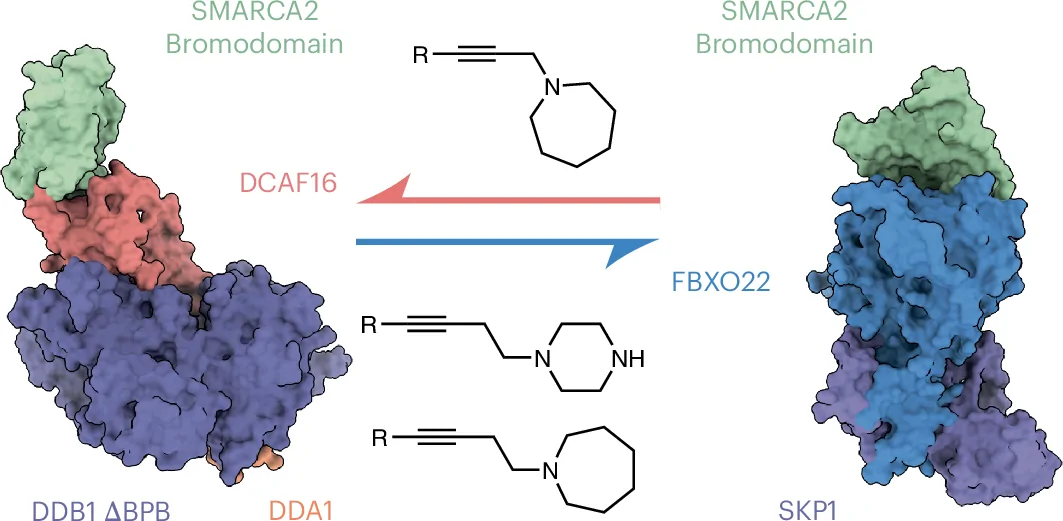

## Abstract (web summary)

The authors identified a series of covalent, monovalent glue degraders that can co-opt two E3 ligases, DCAF16 and FBXO22, in a parallel fashion to degrade SMARCA2/4 and demonstrated that ligase preference can be chemically and genetically tuned.

## Main

Targeted protein degradation (TPD) is a therapeutic modality that can induce potent and selective depletion of protein levels through a catalytic mode of action^1–4^. It relies on small-molecule degraders that function by recruiting a target protein to an E3 ubiquitin ligase, triggering protein ubiquitination and subsequent proteasomal degradation. Degraders are typically categorized into either bifunctional compounds known as proteolysis targeting chimeras (PROTACs), or monovalent compounds known as molecular glue degraders (MGDs). PROTACs consist of two ligands that can individually bind the E3 ligase and the target, which are connected by a linker. In contrast, MGDs typically bind either the E3 ligase or the target protein to create a composite protein-ligand interface that mediates recruitment of the other protein into a ternary complex^5^. Most PROTACs, including all clinically evaluated compounds, co-opt the E3 ligase substrate receptors cereblon (CRBN) or von Hippel–Lindau (VHL)^2,6^. By leveraging chemoproteomics approaches and high-throughput phenotypic screening, additional E3 ligases have been unlocked for PROTAC design^7–12^.

In contrast to the rational design of PROTACs, MGD discovery frequently relies on target-agnostic, phenotypic screening approaches, followed by in-depth mechanistic validation studies^13–19^. Recently, successful target-focused MGD discovery campaigns have been reported, which are based on attaching reactive handles, termed degradation tails, to the solvent-exposed region of the target ligand. This strategy was successfully applied to identify and characterize degraders that recruit additional E3 ligase substrate receptors including DCAF16 (refs. ^11,20–24^), DCAF11 (refs. ^23,25^) and FBXO22 (refs. ^26,27^). To date, all drug-like degraders function through a single E3 ligase. In theory, degraders that depend on more than a single ligase would enable more resilient on-target activity and delay or potentially overcome E3 ligase driven resistance mechanisms^28–32^. In a proof of concept, a dual-ligase strategy has been described by us and others using heterotrivalent PROTACs designed to recruit both VHL and CRBN with a single compound to facilitate the degradation of a target protein^33^. Whether drug-like MGDs could achieve similar dual E3 ligase activity remains to be demonstrated.

Here, we mechanistically characterize a suite of monovalent SMARCA2/4 ligands that work as degraders using genetic screens, biophysical assays and structural deconvolution. We uncover the first example of a monovalent glue that recruits two distinct E3 ligase complexes, a primary driver, CRL4^DCAF16^, and a secondary contributor, CRL1^FBXO22^, using the same degradation tail in a functionally redundant manner. Deep mutational scanning (DMS), cryo-electron microscopy (cryo-EM) and intact mass spectrometry (MS) reveal covalent engagement of DCAF16 through C173. Our cryo-EM structure illuminates the modification in detail, further revealing a previously unresolved loop in DCAF16 that engages the degrader, leading to a distinct orientation of the SMARCA2 bromodomain (BD) relative to DCAF16-dependent BRD4 glues^34,35^. Lastly, we demonstrate that DCAF16 dependency can be chemically and genetically fine-tuned, enabling a framework for the rational design of MGDs with tunable E3 ligase profiles.

## Results

### Compound 1 degrades SMARCA2/4 through FBXO22 and DCAF16

**Fig. 1:**
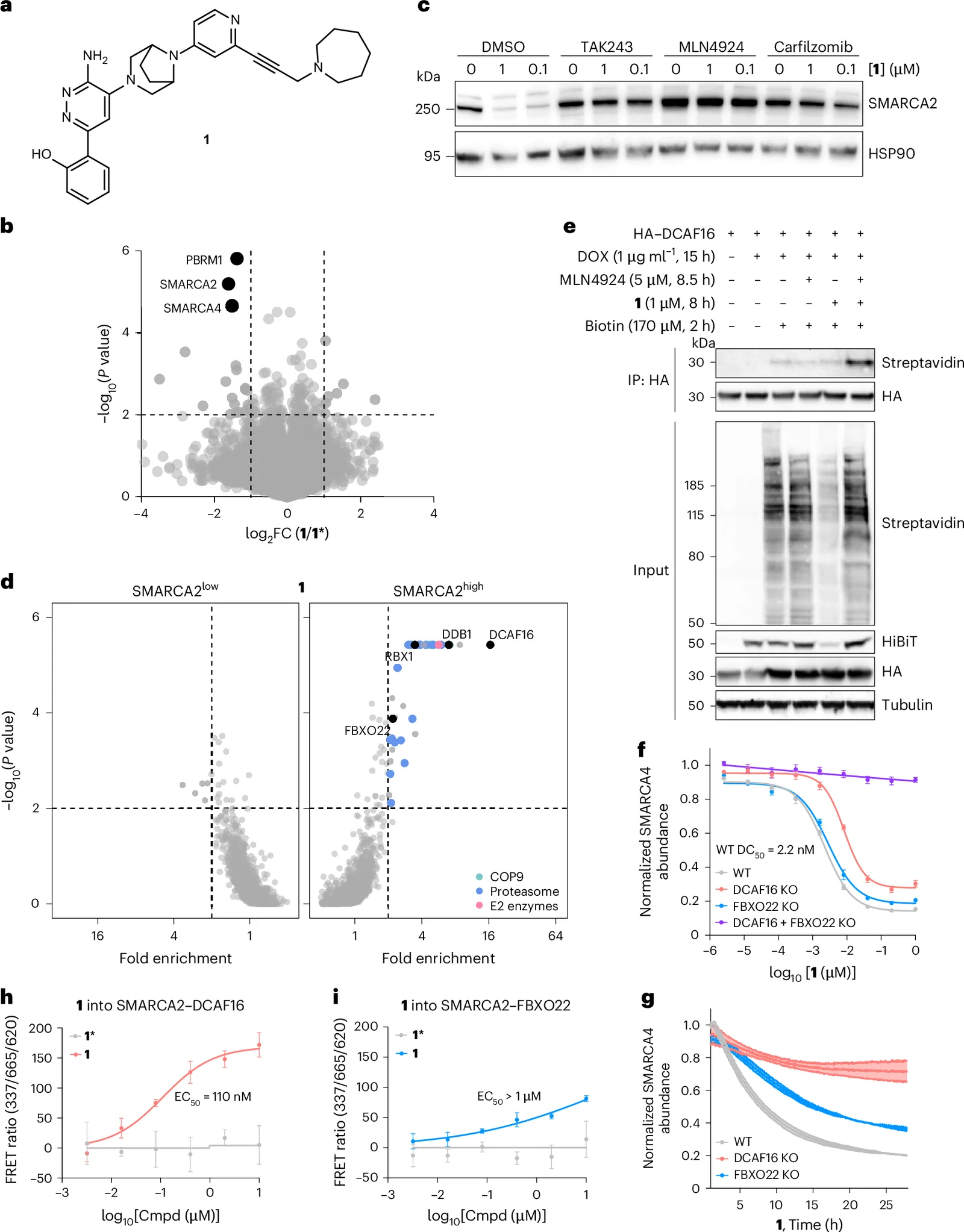
Compound **1** recruits both DCAF16 and FBXO22 to degrade SMARCA2/4. **a**, Structure of **1**. **b**, Whole-proteome changes in HEK293 cells after 12 h of degrader treatment (**1** vs 1*, 1 μM). *P* values were derived from a two-sided permutation-based Student’s *t*-test with 250 randomizations for **1** compared to 1*. Significant hits featured log_2_FC (fold change) ≥ |1| and −log_10_*P* value ≥ 2 (dotted lines). **c**, Degradation rescue by proteasome and neddylation inhibitors. HEK293T cells cotreated with **1** (0.1 or 1 μM) and DMSO, TAK243, MLN4924 or carfilzomib (0.5 μM) for 18 h. **d**, FACS-based CRISPR screen for SMARCA2 stability. SMARCA2 BD stability reporter cells were treated with **1** (0.1 μM) for 24 h and sorted on the basis of SMARCA2–BFP levels. *P* values for the SMARCA2^high^ and SMARCA2^low^ populations were derived versus the SMARCA2^mid^ population using the robust rank aggregation algorithm (MAGeCK). Significant hits featured fold enrichment ≥ 2 and −log_10_*P* value ≥ 2 (dotted lines). **e**, Biotin proximity labeling of DCAF16 by miniTurboID–SMARCA2 BD following treatment with **1** (1 μM, 8 h). HA-tagged DCAF16 was immunoprecipitated and biotinylated proteins were detected by streptavidin immunoblotting. Input and immunoprecipitations were run on separate gels. Input for HiBit was run on a separate gel from the loading control. **f**, HiBiT endpoint degradation assay. SMARCA4–HiBiT cells treated with a dilution series of **1** for 16 h. **g**, HiBiT kinetic degradation assay. Live-cell luminescence measurements of SMARCA4–HiBiT cells treated with **1** (1 μM). **h**,**i**, TR-FRET ternary complex formation assay. Anti-GST–europium bound to GST–SMARCA2 BD was incubated with Cy5-labeled DCAF16–DDB1 (ΔBPB)–DDA1 (**h**) or Cy5-labeled FBXO22–SKP1 (**i**) and increasing concentrations of **1** or 1*. Data shown are from *n* = 3 independent replicates (**b**,**g**,**h**,**i**), *n* = 2 independent replicates (**d**) or *n* = 6 independent replicates (**f**). Data represent the mean ± s.e.m. (**f**–**i**). Source data

**Extended Data Fig. 1:**
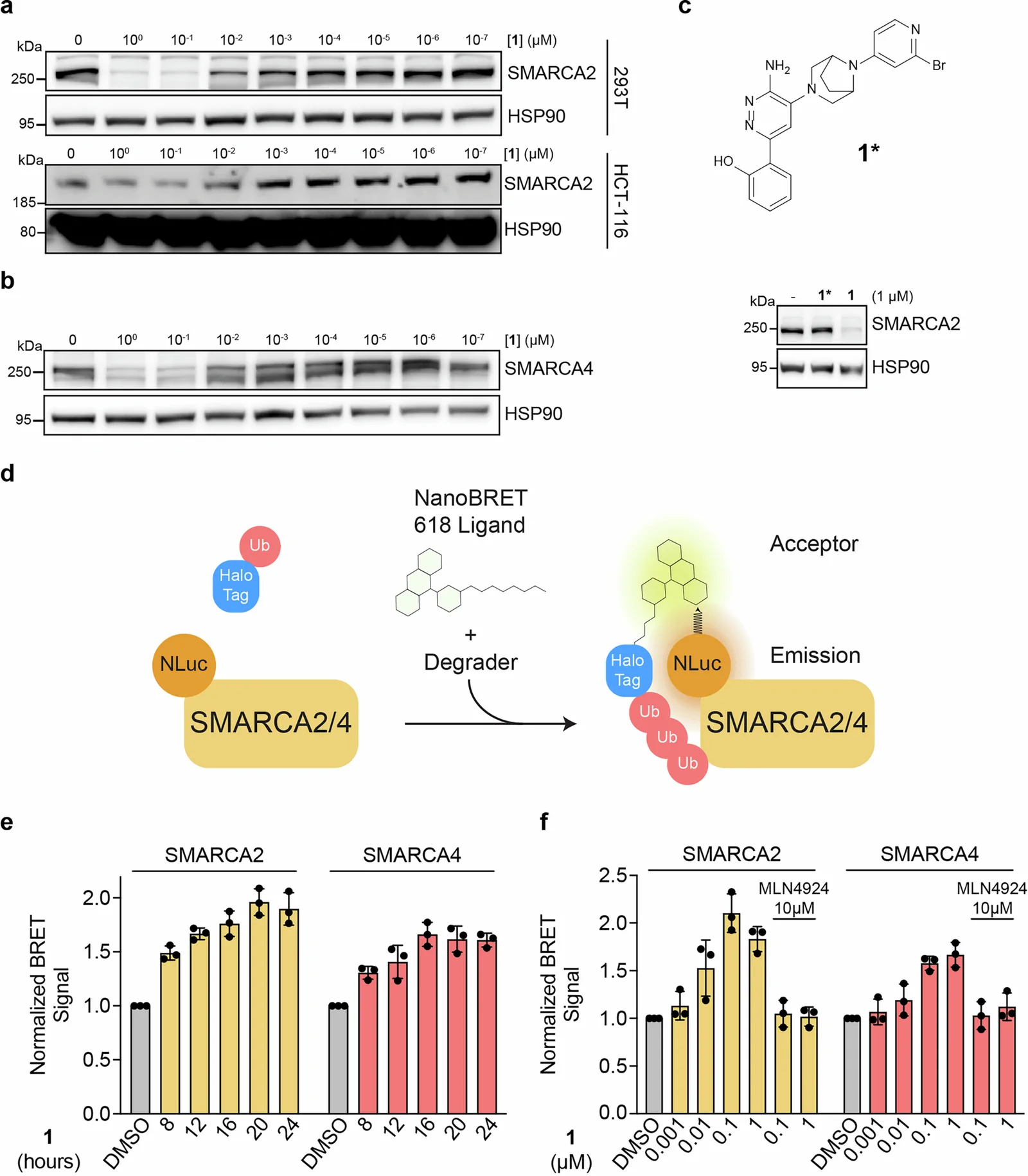
Compound 1 induces ubiquitination and degradation of SMARCA2/4. **a**, **b**, Compound **1** induced degradation of SMARCA2 (**a**) and SMARCA4 (**b**). HEK293T and HCT116 cells treated with a dilution series of **1** for 24 h. **c**, Structure of inactive control compound (1*) and immunoblot of SMARCA2 in HEK293T cells after treatment with DMSO, 1*, or **1** (1 µM) for 24 hours. **d**, Schematic of NanoBRET ubiquitination assay to evaluate cellular ubiquitination of SMARCA2/4 in the presence of a degrader. **e**, Kinetic NanoBRET ubiquitination assay. SMARCA2/4-HiBiT cells transfected with LgBiT and HaloTag-Ubiquitin were treated with **1** (0.1 µM) for various time points. **f**, Concentration-based NanoBRET ubiquitination assay. SMARCA2/4-HiBiT cells transfected with LgBiT and HaloTag-Ubiquitin were treated with increasing concentrations of **1** ( ± 10 µM MLN4924, 1 hour pre-treatment) for 24 hours. For **e**, **f**, n = 3 independent replicates. Data represent mean ± SD for **e**-**f**. Source data

A recent patent filing (WO2023018648) reported a SMARCA2/4 degrader, herein referred to as compound **1** (**1**) (Fig. 1a), which comprises a SMARCA2/4 BD ligand bearing a propargyl-azepane tail group^36^. Compound **1** was found to induce degradation of SMARCA2/4 in HEK293T and HCT-116 cells (Extended Data Fig. 1a,b) and quantitative MS proteomics confirmed degradation selectivity toward SMARCA2/4 and PBRM1 (Fig. 1b and Supplementary Table 1)^37^. In contrast, 1*, a close analog of **1** lacking the propargyl-azepane tail, had no degradation activity (Extended Data Fig. 1c), indicating that this moiety is essential for degradation. SMARCA2 degradation was prevented by inhibiting the ubiquitin-activating enzyme UBA1 (TAK243), neddylation (MLN4924) and the proteasome (carfilzomib) (Fig. 1c), consistent with Cullin–RING ligase (CRL)-mediated degradation. In support of this, NanoBRET ubiquitination assays in live cells (Extended Data Fig. 1d) showed dose-dependent and time-dependent increases in SMARCA2/4 ubiquitination after treatment with **1**, which was abolished by MLN4924 treatment (Extended Data Fig. 1e,f).

**Extended Data Fig. 2:**
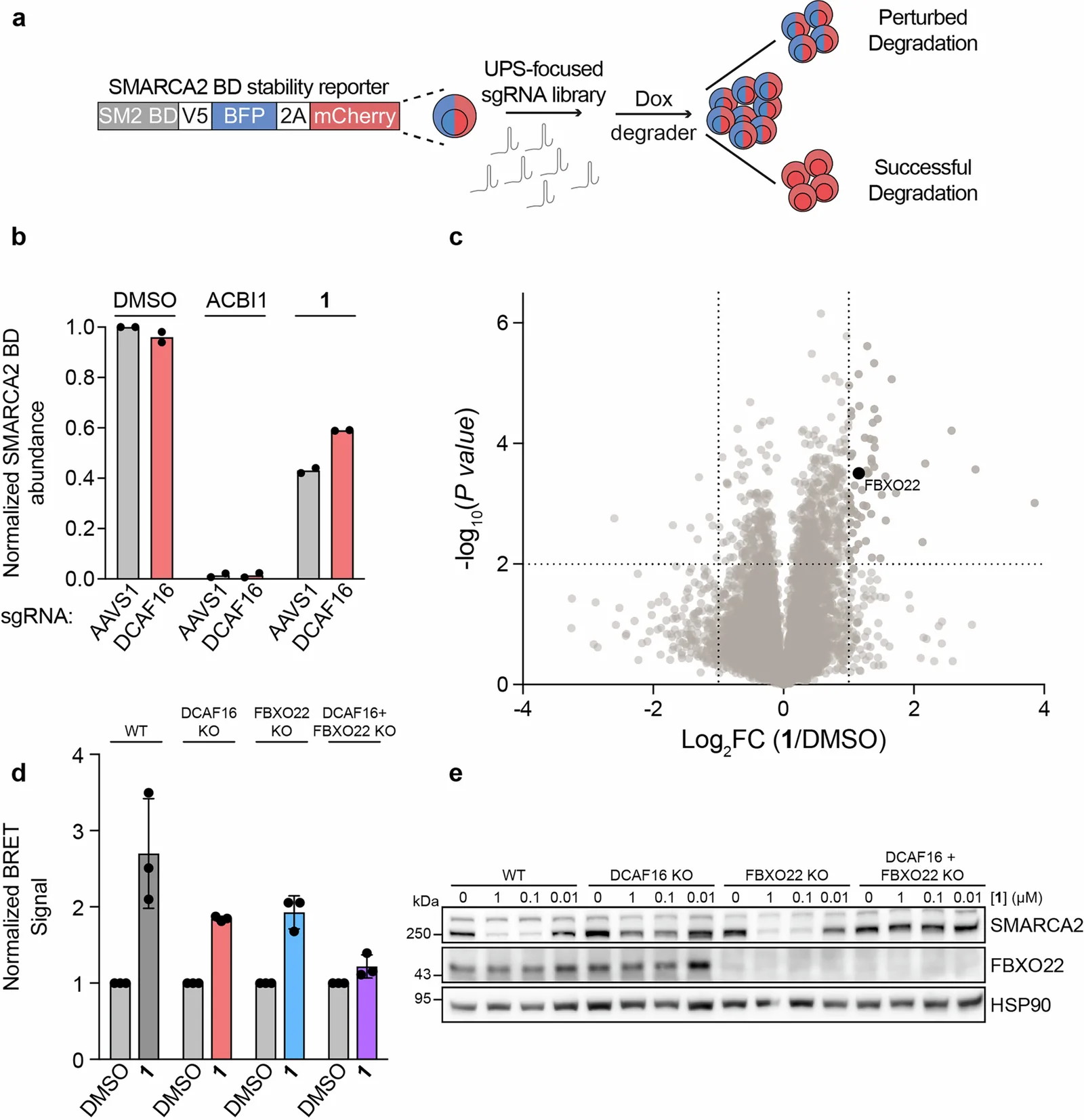
Compound 1 degrades SMARCA2/4 via DCAF16 and FBXO22. **a**, Schematic for FACS-based CRISPR-Cas9 screen with a SMARCA2 stability reporter**. b**, CRISPR screen validation. SMARCA2 BD stability reporter cells transduced with AAVS1 or DCAF16-targeting sgRNAs were treated with DMSO, ACBI1 (1 µM), or **1** (1 µM) for 24 hours. Data represent mean. **c**, BioID analysis with **1** treatment. Proximity labelling in HEK293 Flp-In T-REx miniTurboID-SMARCA2-BD inducible cell line (10 µM of **1** vs DMSO). P values derived from two-sided permutation-based Student’s t-test with 250 randomizations for **1** compared to DMSO. Significant hits: Log_2_FC ≥ |1| and -log_10_P value ≥ 2 (dotted lines). **d**, NanoBRET ubiquitination assay in KO cell lines. SMARCA4-HiBiT cells with single and double E3 ligase KOs transfected with LgBiT and HaloTag-Ubiquitin were treated with DMSO or **1** (1 µM) for 24 hours. **e**, Genetic rescue of SMARCA2 degradation. HEK293T cells with *DCAF16* and *FBXO22* KOs treated with DMSO or increasing concentrations of **1** for 24 hours. For **c**, **d**, n = 3 independent replicates, for **b**, n = 2 independent replicates. Data represent mean ± SD for **d**. Source data

To identify the CRL required for the activity of **1**, we set up a time-controlled, fluorescence-activated cell sorting (FACS)-based CRISPR screen leveraging a dual fluorescence SMARCA2 BD stability reporter and a ubiquitin–proteasome system (UPS)-focused single guide RNA (sgRNA) library (Extended Data Fig. 2a)^26^. Following treatment with **1**, the substrate receptor DCAF16 alongside other CRL4^DCAF16^ core complex members, DDB1 and RBX1, emerged as top hits. As expected, we also identified components of the 20S proteasome and COP9 signalosome (Fig. 1d and Supplementary Table 2), again indicative of a CRL-dependent and proteasome-dependent mechanism. To validate the role of DCAF16 in driving **1**-mediated degradation of SMARCA2, we used a proximity-dependent biotin identification (BioID) approach based on miniTurboID-tagged SMARCA2 BD and HA-tagged DCAF16 (ref. ^38^). Treatment with **1** resulted in enhanced biotinylation of DCAF16, demonstrating induced proximity of both proteins upon compound treatment (Fig. 1e). However, to our surprise, knockout (KO) of *DCAF16* failed to fully abrogate degradation of the SMARCA2 BD stability reporter by **1** (Extended Data Fig. 2b). Thus, we wondered whether additional factors are redundantly involved in SMARCA2/4 degradation induced by **1**. To that end, we conducted SMARCA2 BD proximity labeling through unbiased proteomics, demonstrating a significant (*P* value = 3.14 × 10^−4^, fold change = 2.23) increase in biotinylation of the CRL1 substrate receptor FBXO22 in the presence of **1** (Extended Data Fig. 2c and Supplementary Table 3). Intriguingly, even though genetic loss-of-function approaches frequently fail to resolve genetic redundancies, FBXO22 was also identified as a weak hit in the FACS-based CRISPR screen (Fig. 1d and Supplementary Table 2), suggesting the involvement of two unrelated ligases, CRL4^DCAF16^ and CRL1^FBXO22^, in SMARCA2/4 degradation mediated by **1**.

To validate this hypothesis, we generated single-KO and double-KO cell lines for both *DCAF16* and *FBXO22*. Through western blotting and NanoBRET ubiquitination, we found that only the double-KO was able to completely ablate ubiquitination and degradation of SMARCA2/4 mediated by **1** (Extended Data Fig. 2d–e). A similar trend was observed in dose-response HiBiT degradation assays for endogenously tagged SMARCA4 (wild type (WT) half-maximal degradation concentration (DC_50_) = 2.2 nM; Fig. 1f). Moreover, *DCAF16* and *FBXO22* single-KO cell lines showed slowed but not abrogated degradation in time-course experiments (Fig. 1g). Together, these experiments suggest that both ligases are functionally required to elicit SMARCA2/4 degradation by **1**. To assess whether both ligases also form a ternary complex with SMARCA2 BD in the presence of **1** in vitro, we performed time-resolved fluorescence resonance energy transfer (TR-FRET) assays. Indeed, **1** induced dose-dependent ternary complex formation between SMARCA2 BD and both CRLs, which was more potent for DCAF16 (half-maximal effective concentration (EC_50_) = 110 nM) compared to FBXO22 (EC_50_ > 1 μM; Fig. 1h,i). No complex formation was observed in the presence of the nondegrading analog 1*. Together, our data are consistent with an unprecedented glue degrader mechanism that co-opts two distinct E3 ligase complexes: CRL4^DCAF16^ as the primary driver mediating degradation and CRL1^FBXO22^ as a secondary contributor.

### Compound 1 covalently adducts DCAF16 at C173

**Fig. 2:**
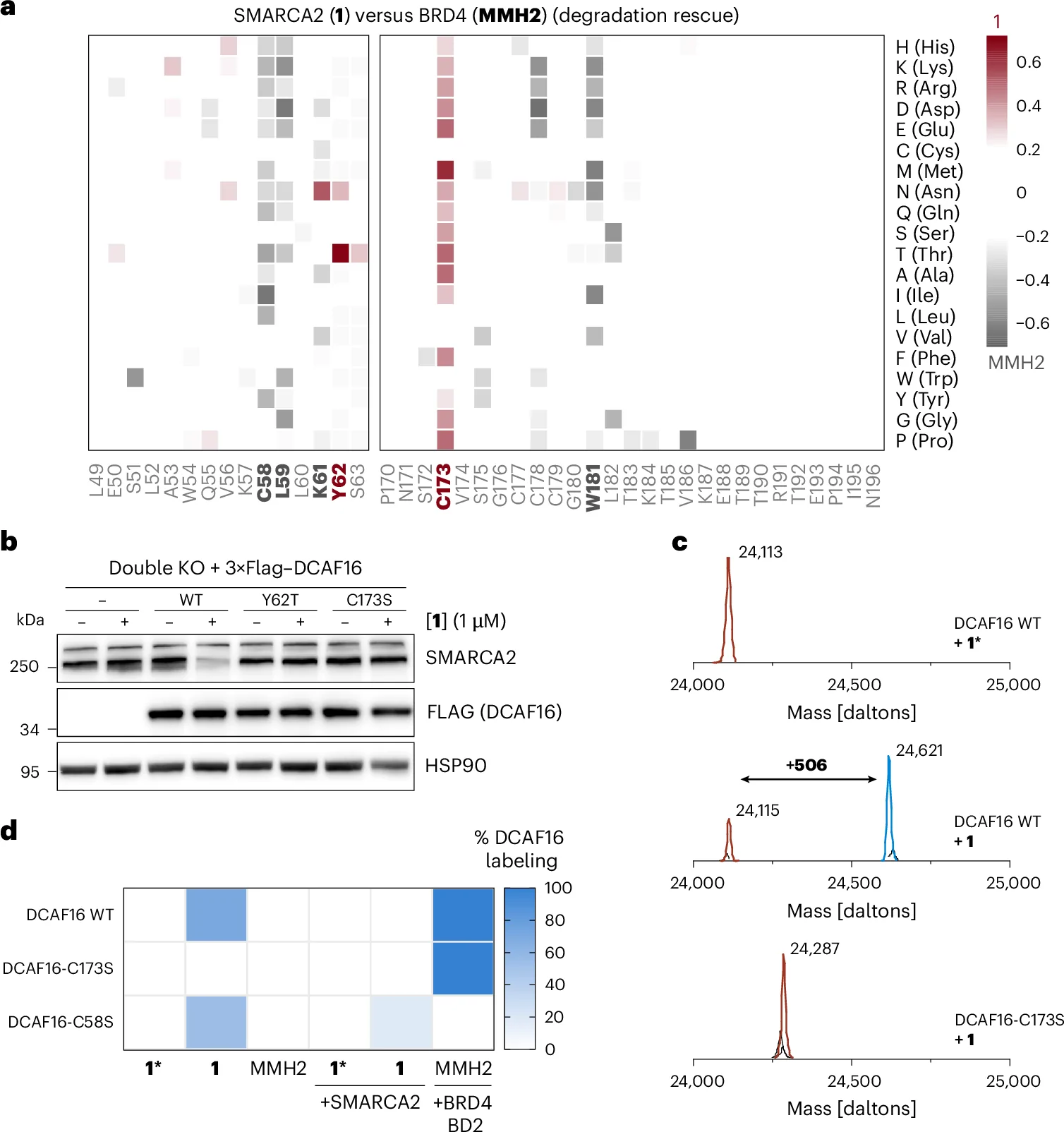
Compound **1** covalently engages with DCAF16 through C173. **a**, DMS of DCAF16 for degradation rescue. *DCAF16* and *FBXO22* double-KO HEK293T cells stably expressing stability reporters for SMARCA2 BD or BRD4 tandem BDs were transduced with a library of EGFP-tagged DCAF16 variants containing single-amino-acid substitutions at every position. SMARCA2 and BRD4 stability reporter cells were treated with **1** (1 µM) and MMH2 (1 µM) for 24 h, respectively. Heat map depicting differential log_2_ fold enrichment of DCAF16 substitutions normalized to maximum log_2_FC versus unsorted control between SMARCA2 targeting compound **1** and BRD4 targeting MMH2. Data correspond to sorted SMARCA2^high^ and BRD4^high^ populations, showing substitutions that specifically prevent SMARCA2 degradation (red) and BRD4 degradation (gray) (*n* = 3 independent measurements). **b**, Validation of DMS. Double-KO HEK293T cells stably expressing 3×Flag tagged DCAF16 variants treated with **1** (1 µM) for 24 h. **c**, Intact protein mass spectra for WT DCAF16 coincubated with inactive control 1* (top), WT DCAF16 coincubated with **1** (middle) and DCAF16-C173S mutant coincubated with **1** (bottom). Red peaks represent unmodified DCAF16 and blue peaks represent covalently modified DCAF16. Covalent adduct formation of DCAF16–**1** is shown by the arrow. All incubations were performed with 10 µM DCAF16 and 20 µM 1* or **1** at room temperature for 18 h. **d**, Proportion of labeled DCAF16. Heat map depicting the proportion (%) of labeling from intact MS for each DCAF16 variant (10 µM) in the presence of 1*, **1** or MMH2 (all at 20 µM). Intact MS experiments for DCAF16 were performed with or without the presence of SMARCA2 BD or BRD4 BD2 (both at 20 µM). Source data

**Extended Data Fig. 3:**
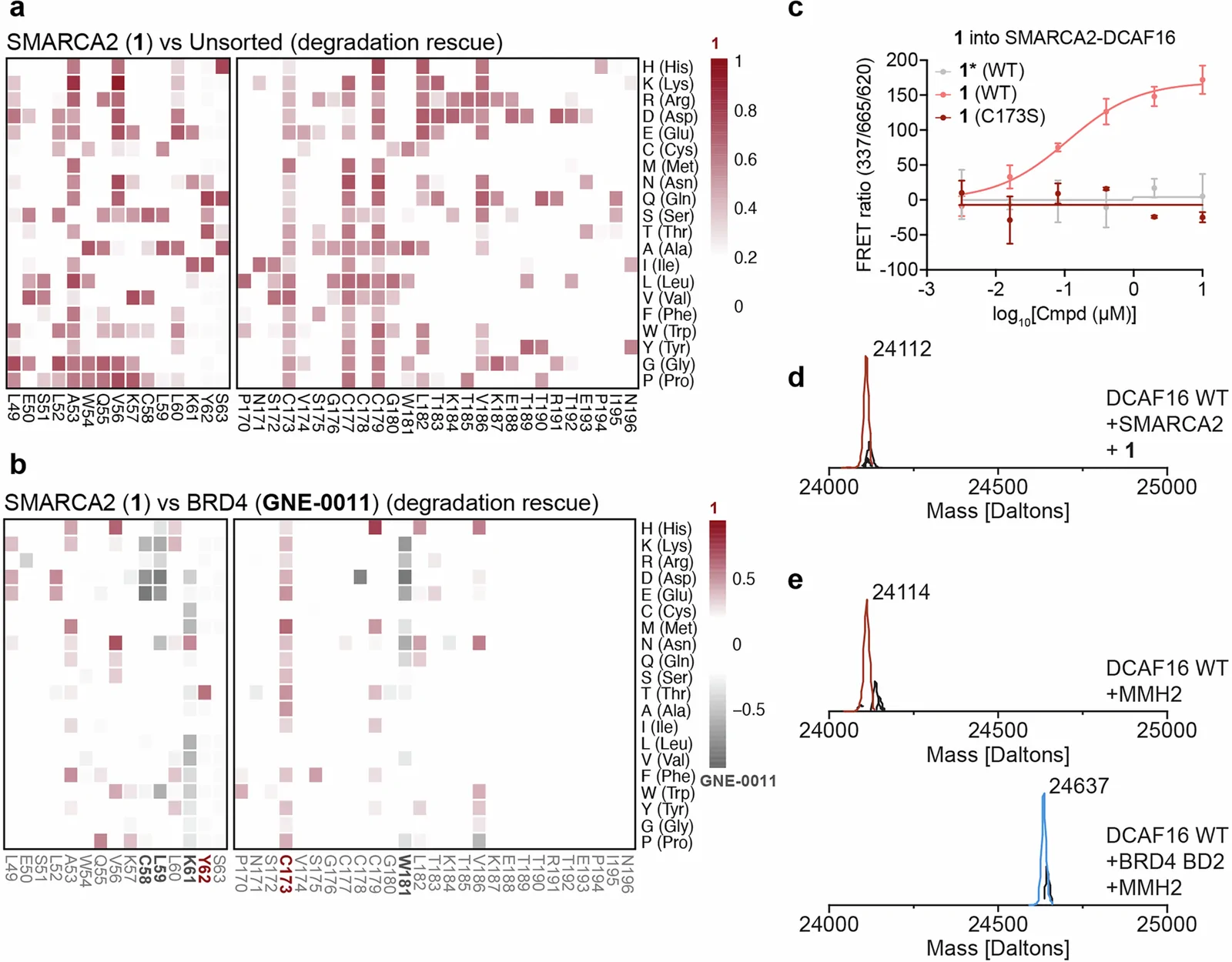
Compound 1 does not engage with DCAF16 in a template-assisted manner. **a**, **b**, DMS of DCAF16 for degradation rescue. *DCAF16* and *FBXO22* double-knockout HEK293T cells stably expressing stability reporters for SMARCA2 BD or BRD4 tandem-BDs were transduced with a library of EGFP tagged DCAF16 variants containing single amino acid substitutions at every position. SMARCA2 and BRD4 stability reporter cells were treated with **1** (1 µM) and GNE-0011 (0.1 µM) for 24 hours, respectively. Heatmap depicting mean log_2_ fold enrichment of DCAF16 mutations normalized to maximum log_2_ fold changes for **1** versus unsorted control (**a**). Heatmap depicting differential log_2_ fold enrichment of DCAF16 mutations normalized to maximum log_2_ fold changes versus unsorted control between SMARCA2 targeting **1** and BRD4 targeting GNE-0011 (**b**). Data corresponds to SMARCA2^High^ and BRD4^High^ sorted populations, showing mutations that specifically prevent SMARCA2 degradation (red) and BRD4 degradation (gray), n = 3 independent replicates. **c**, TR-FRET ternary complex formation assay. Anti-GST–europium bound to GST–SMARCA2 BD was incubated with Cy5-labelled DCAF16(C173S)–DDB1(ΔBPB)–DDA1 and increasing concentrations of **1**. Data represent mean ± SEM, n = 2 independent replicates. TR-FRET data for DCAF16 C173S is overlayed onto TR-FRET data corresponding to WT DCAF16 from Fig. 1h. **d**, Intact protein mass spectra for WT DCAF16 (10 µM) and **1** (20 µM) in the presence of SMARCA2 BD (20 µM). **e**, Intact protein mass spectra for WT DCAF16 (10 µM) and MMH2 (20 µM) in the absence (top) or presence (bottom) of BRD4 BD2 (20 µM). Red peaks represent unmodified DCAF16 and blue peaks represent covalently modified DCAF16. Source data

To identify functional hotspots on DCAF16, we performed DMS of the full-length protein. This enabled us to systematically assess the effects of every possible point substitution (4,300 variants in total) in DCAF16 on SMARCA2 BD degradation mediated by **1**. We performed the screen in *DCAF16* and *FBXO22* double-KO HEK293T cells to eliminate background interference from endogenous ligases. To distinguish context-specific effects from general defects caused by mutations that disrupt overall DCAF16 stability, we concurrently monitored BRD4 BD1–BD2 degradation mediated by two previously described DCAF16-dependent monovalent glue degraders, MMH2 and GNE-0011 (refs. ^34,39^). Comparative analysis between degrader-specific variants revealed that most substitutions in C173 profoundly disrupted SMARCA2 degradation induced by **1** (Fig. 2a, Extended Data Fig. 3a,b and Supplementary Table 4). Exceptions constituted a subset of substitutions, including C173L, C173V and C173W, which likely disrupt stability of DCAF16. Hence, these substitutions abrogate degradation for both SMARCA2 and BRD4 and are consequently not resolved by a differential analysis. Substitutions at C173 contrast with substitutions at C58, L59, K61 and W181, which specifically affect degradation of BRD4 BD1–BD2 mediated by MMH2 or GNE-0011, as previously shown^34^. Additionally, Y62T was shown to disrupt SMARCA2 degradation induced by **1**. The impact of both C173S and Y62T was confirmed by western blot (Fig. 2b). TR-FRET titrations further demonstrated that **1** cannot induce ternary complex formation between SMARCA2 and DCAF16 C173S (Extended Data Fig. 3c). Thus, the results identify C173 as a hotspot for the mode of action of **1** that is unique compared to known DCAF16-dependent BRD4 degraders, suggesting a differentiated mechanism of ligase recognition and a potentially altered architecture of the induced ternary complex.

Given the importance of C173, we investigated whether **1** recruits DCAF16 through a covalent mechanism. Leveraging intact protein MS, we demonstrated covalent labeling of recombinant WT DCAF16 and DCAF16-C58S by **1**, while labeling was abrogated for the DCAF16-C173S mutant (Fig. 2c,d), indicating that **1** specifically covalently adducts C173. Interestingly, no labeling of DCAF16 was observed upon coincubation with SMARCA2 BD (Fig. 2d and Extended Data Fig. 3d). This pattern markedly contrasts with MMH2, where labeling of DCAF16 depends on coincubation with BRD4 BD2 (Fig. 2d and Extended Data Fig. 3e). This suggests that, unlike the template-assisted covalent mechanism of MMH2, which is recapitulated in our experimental setup, labeling of DCAF16 is not templated by the SMARCA2 BD, at least at the micromolar range of concentrations used in these experiments^34^. Collectively, the biophysical and unbiased genetics data show that **1** mediates the formation of a ternary complex between the SMARCA2/4 BDs and its driver ligase DCAF16 through covalent modification of C173 on DCAF16.

### Structure of SMARCA2 and compound 1 glued to DCAF16

**Fig. 3:**
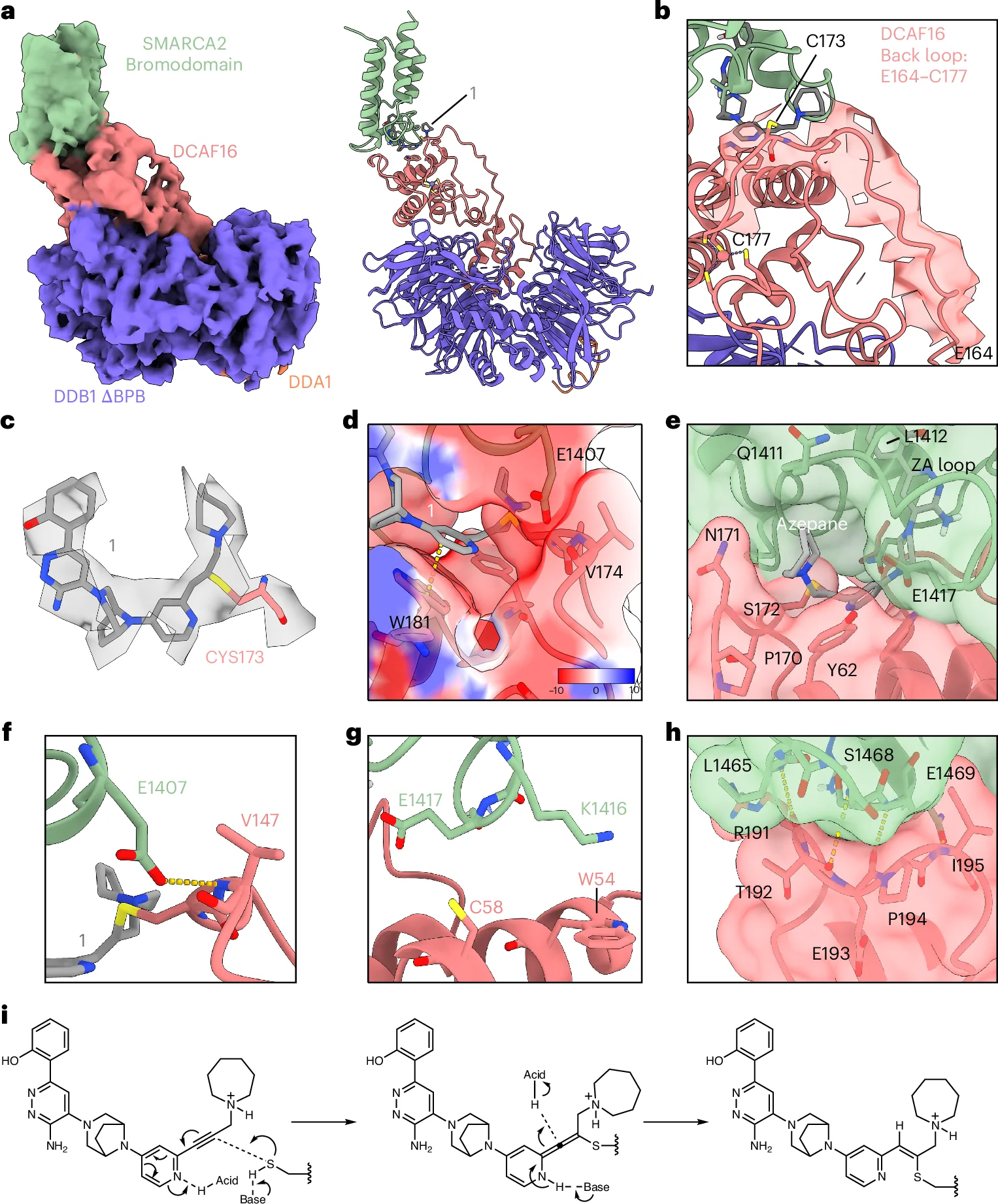
Cryo-EM structure of the ternary complex formed between compound **1**, SMARCA2 and DCAF16. **a**, Structure of the complex between DCAF16 (red), DDB1(ΔBPB) (purple), DDA1 (orange) and SMARCA2 BD (green), glued by **1** (gray), solved by cryo-EM at a global resolution of 3.47 Å. The electron density (left) and atomic model (right) are shown. **b**, Electron density of the newly resolved back loop (E164–C177) of DCAF16 that harbors C173 and is covalently engaged by **1**. **c**, Electron density around **1** and C173. **d**, Electrostatic surface potential showing area of negative potential (red) around the pyridine ring of **1** because of the presence of the V174 backbone carbonyl (DCAF16) and E1407 side chain (SMARCA2). The pyridine of **1** stacks with W181 of DCAF16. **e**, The azepane ring of **1** nestles into a pocket formed by residues Q1411–L1412 of SMARCA2 and P170–S172 of DCAF16, while also stacking with Y62 of DCAF16. **f**, Protein–protein interaction between the side-chain carboxylate of E1407 on SMARCA2 and the backbone amide of V174 on DCAF16. **g**, K1416 and E1417 from the ZA loop of SMARCA2 BD point toward W54 and C58 in DCAF16. **h**, SMARCA2 BD BC loop residues, L1465–E1469, hydrogen bond with residues R191–I195 on a DCAF16 loop. **i**, Schematic showing the predicted covalent attachment of **1** to the side chain of C173.

**Extended Data Fig. 4:**
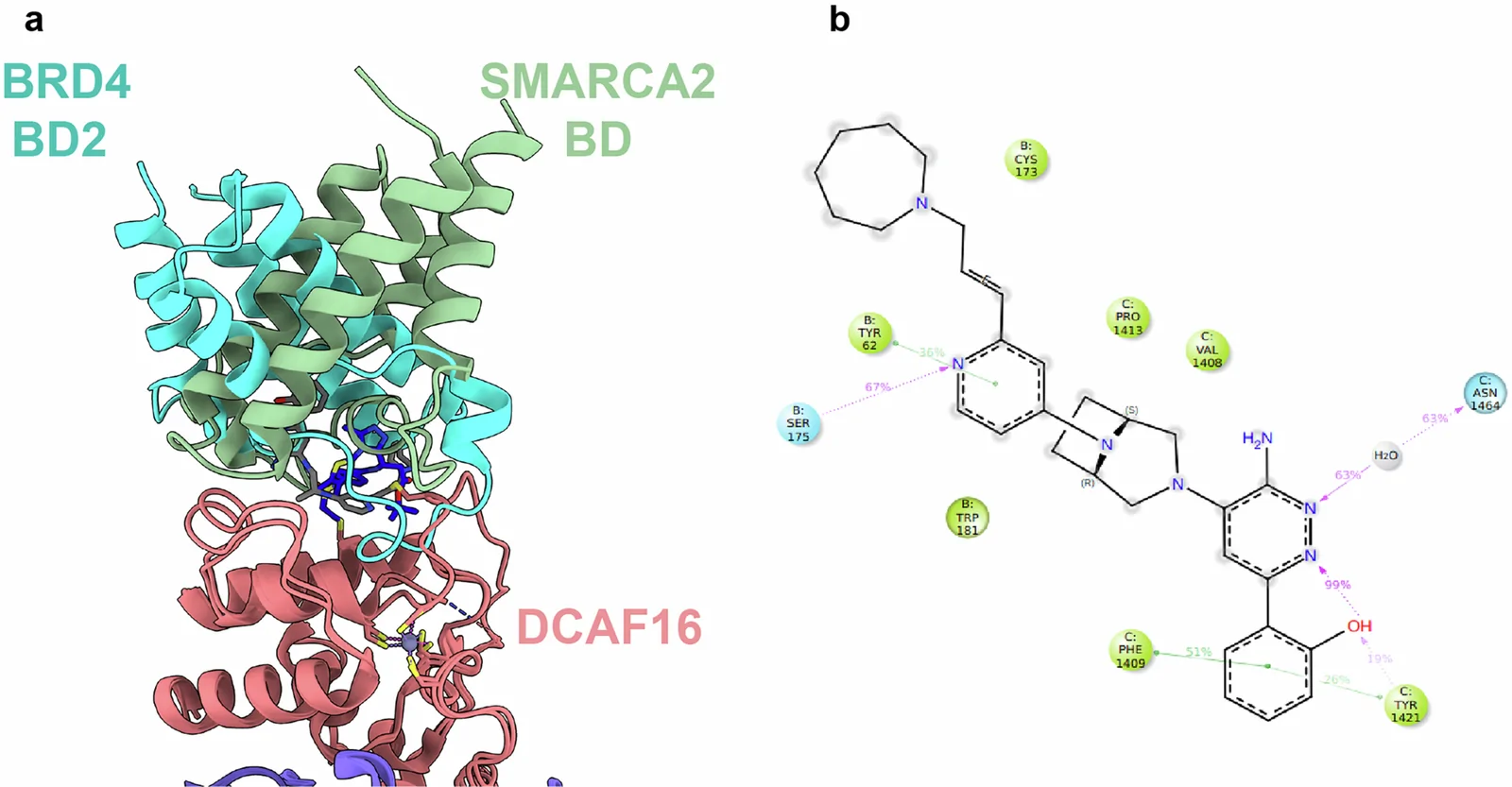
Compound 1 glues SMARCA2 to DCAF16 in a structurally distinct manner relative to BRD4. **a**, Super-imposed binding mode of BRD4 BD2 (PDB ID: 8G46) and SMARCA2 BD on DCAF16. **b**, Protein-ligand interactions map from molecular dynamics simulation of DCAF16:DDB1 (ΔBPB): DDA1, SMARCA2, **1** ternary complex.

To gain a structural understanding of the mechanism underpinning **1**-induced SMARCA2 BD degradation, we solved the structure of the ternary complex involving SMARCA2 BD, **1** and DCAF16:DDB1(ΔBPB):DDA1 by cryo-EM with a resolution of approximately 3.47 Å (Fig. 3a and Supplementary Figs. 1 and 2). We observed a clear density for SMARCA2 BD sitting on top of DCAF16 in an orientation tilted slightly relative to the core of the ligase complex. We detected density corresponding to a back loop (residues E164–C177; Fig. 3b) that was unresolved in previously published structures of DCAF16 (PDB 8OV6 and 8G46)^34,35^. Strikingly, a clear continuous density is seen between C173 that resides on this loop and the compound, consistent with formation of a covalent bond (Fig. 3b,c). The position of the SMARCA2 BD is distinct relative to the MMH2-induced position of BRD4 BD2 (PDB 8G46). SMARCA2 adopts a backward-tilted orientation toward the DCAF16 loop region, in contrast to the forward tilt observed for BRD4 BD2 (Extended Data Fig. 4a). This difference likely arises from covalent labeling of distinct cysteine residues on DCAF16 and highlights the structural flexibility of DCAF16 in accommodating similar BD conformations in distinct orientations, thereby enabling adaptable degrader engagement across diverse targets.

In addition to the covalent bond with C173, **1** forms extensive direct interactions with DCAF16. The pyridine group of **1** engages in a *π* stacking interaction with W181 of DCAF16 (Fig. 3d), directing the degradation tail to insert at the interface between the ZA loop of the SMARCA2 BD and DCAF16 (Fig. 3e). Specifically, the terminal azepane ring is optimally positioned to fit into a pocket defined by Q1411–L1412 on the BD, P170–S172 on the DCAF16 loop and Y62 on DCAF16 (Fig. 3e). DCAF16 Y62 was also identified as functionally relevant by DMS (Fig. 2a). The pyridine of the degradation tail sits in a negatively charged pocket, including the E1407 side-chain carboxylate from the ZA loop of SMARCA2 and the backbone carbonyl of V174 on DCAF16 (Fig. 3d). This environment suggests that the pyridine is protonated, further activating the alkyne for nucleophilic attack. Moreover, a molecular dynamics (MD) simulation of the ternary complex revealed that S175 on DCAF16 can hydrogen bond with the nitrogen in the pyridine ring of **1** (Extended Data Fig. 4b). Beyond the direct interactions formed between the degrader and DCAF16, we observe extensive induced protein–protein contacts at the ternary complex interface. These contacts bury a total surface area of 1,057 Å^2^, comparable to other PROTAC and MGD ternary structures, ranging from 680 to 2,672 Å^2^ (https://www.ebi.ac.uk/pdbe/prot_int/pistart.html)^5,40^. Several protein–protein interactions stabilize the ternary complex, including a hydrogen bond between the side-chain carboxylate of E1407 on SMARCA2 and the backbone amide of V174 on DCAF16 (Fig. 3f). On the other side of the complex, K1416 and E1417 from the ZA loop of SMARCA2 BD point toward a helix in DCAF16 containing C58, which is covalently engaged by previously reported MGDs (Fig. 3g)^34,41^. Lastly, at the front end of the interface, SMARCA2 BD residues from L1465 to E1469 in the BC loop form hydrogen bonds and exhibit close side-chain packing with the R191–I195 loop of DCAF16 (Fig. 3h). The direct degrader–ligase contacts and induced protein–protein interactions orient SMARCA2 BD and DCAF16 into a compact ternary complex, stabilized by covalent bonding, hydrogen bonds, *π* stacking and extensive surface burial across several interacting loop regions.

Taken together, the cryo-EM structure unveils, in atomic detail, the covalent modification of DCAF16 at C173 by **1**, and recruitment of SMARCA2. This, coupled with the biophysical and mutagenesis data, supports proposal of a mechanism for covalent labeling of DCAF16 by **1**, where C173 undergoes a nucleophilic addition to the electron-deficient acetylene moiety of the compound, which is activated by the electron-poor pyridine ring. This leads to regiospecific and stereospecific covalent bond formation and recruitment of the SMARCA2/4 BD to the modified DCAF16 (Fig. 3i).

### Chemical tunability of E3 ligase selectivity

**Fig. 4:**
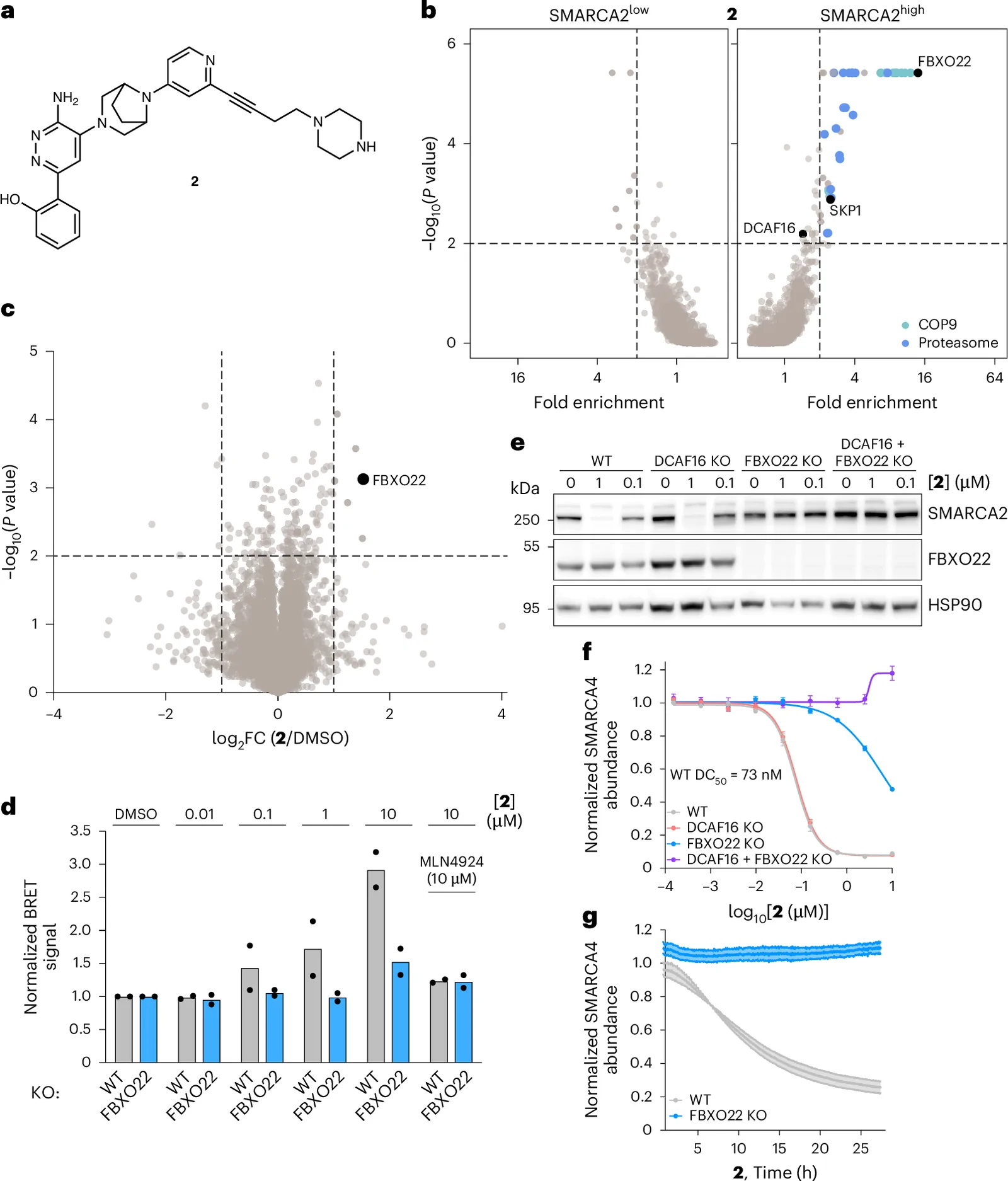
Compound 2 exhibits a driver ligase switch from DCAF16 to FBXO22. **a**, Structure of **2**. **b**, FACS-based CRISPR screen for SMARCA2 stability. SMARCA2 BD stability reporter cells were treated with **2** (1 µM) for 24 h and sorted on the basis of SMARCA2–BFP levels. *P* values for SMARCA2^high^ and SMARCA2^low^ populations were derived versus the SMARCA2^mid^ population using the robust rank aggregation algorithm (MAGeCK). Significant hits feature fold enrichment ≥ 2 and −log_10_*P* value ≥ 2 (dotted lines). **c**, BioID analysis with **2** treatment. Proximity labeling in HEK293 Flp-In T-REx miniTurboID–SMARCA2 BD inducible cell line (10 µM of **2** versus DMSO). *P* values were derived from a two-sided permutation-based Student’s *t*-test with 250 randomizations for **2** compared to DMSO. Significant hits feature log_2_FC ≥ |1| and −log_10_*P* value ≥ 2 (dotted lines). **d**, NanoBRET ubiquitination assay. SMARCA4–HiBiT cells transfected with LgBiT and HaloTag–ubiquitin were treated with increasing concentrations of **2** (±10 µM MLN4924, 1 h of pretreatment) for 24 h. Data represent the mean. **e**, Genetic rescue of SMARCA2 degradation. HEK293T cells with *DCAF16* and *FBXO22* KO treated with DMSO or **2** (0.1 and 1 µM) for 24 h. **f**, HiBiT endpoint degradation assay. SMARCA4–HiBiT cells were treated with a dilution series of **2** for 24 h. **g**, HiBiT kinetic degradation assay. Live-cell luminescence measurements of SMARCA4–HiBiT cells treated with **2** (1 µM). Data shown are from *n* = 2 independent replicates (**b**,**d**), *n* = 3 independent replicates (**c**,**g**) or *n* = 6 independent replicates (**f**). Data represent the mean ± s.e.m. (**f**,**g**). Source data

**Extended Data Fig. 5:**
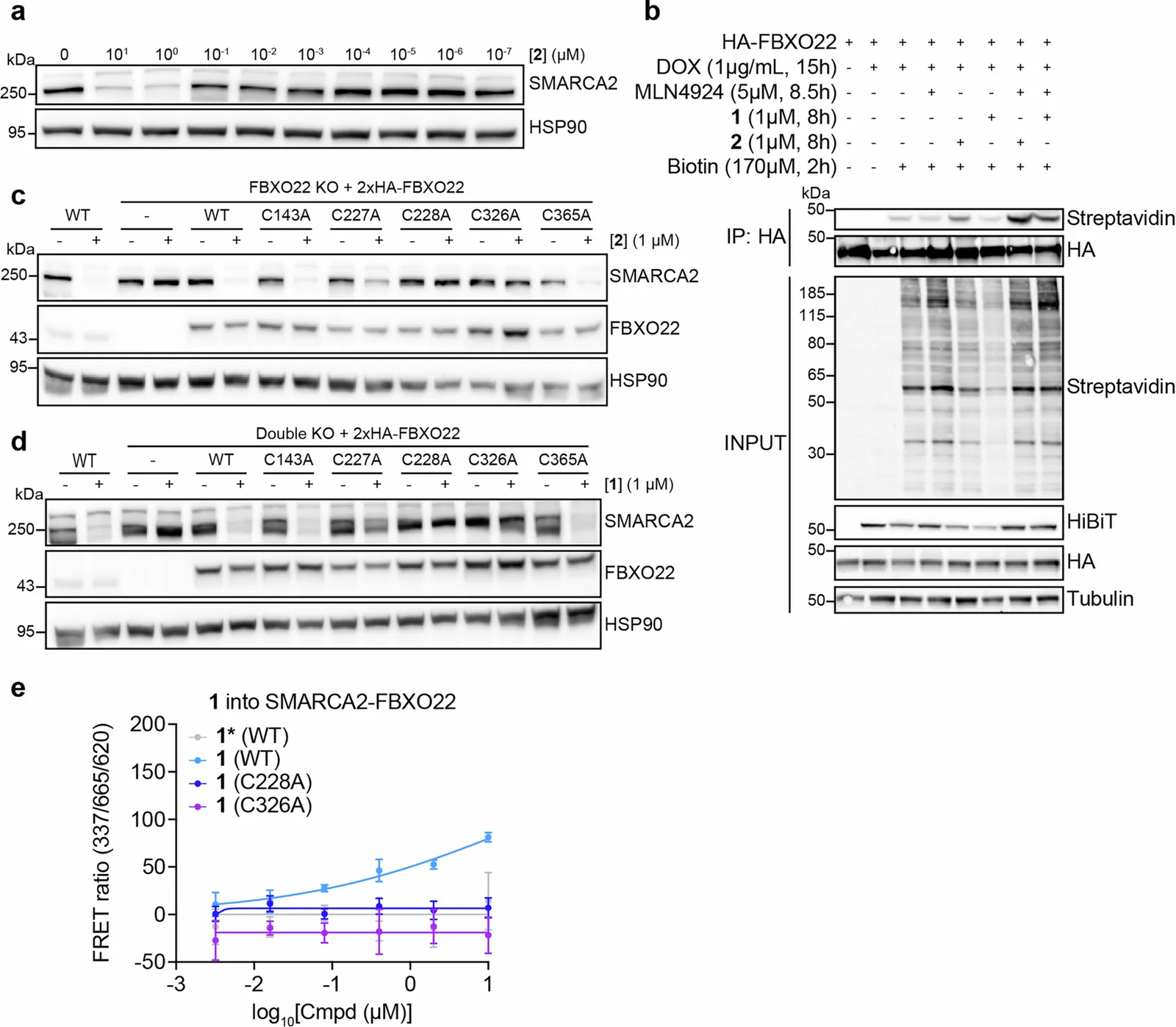
Monovalent SMARCA2/4 degraders engage with FBXO22 via C228 and C326. **a**, Degradation of SMARCA2 by **2**. HEK293T cells treated with a dilution series of **2** for 24 h. **b**, Biotin proximity labelling of FBXO22 by miniTurboID-SMARCA2-BD following treatment with **1** or **2** (1 μM, 8 hours). HA-tagged FBXO22 was immunoprecipitated and biotinylated proteins were detected via streptavidin immunoblotting. Input and immunoprecipitations were run on separate gels. **c**, **d**, Immunoblots with FBXO22 Cysteine mutants. *FBXO22* KO HEK293T cells stably expressing HA tagged FBXO22 variants treated with **2** (1 µM) for 24 hours (**c**). Double-KO HEK293T cells stably expressing HA tagged FBXO22 variants treated with **1** (1 µM) for 24 hours (**d**). **e**, TR-FRET ternary complex formation assay. Anti-GST–europium bound to GST–SMARCA2 BD was incubated with Cy5-labelled FBXO22(C228A or C326A)–SKP1 and increasing concentrations of **1**. Data represent mean ± SEM, n = 3 independent replicates. TR-FRET data for FBXO22 C228A and C326A is overlayed onto TR-FRET data corresponding to WT FBXO22 from Fig. 1i. Source data

To investigate how changes to the degradation tail might impact ligase recruitment, we aimed to characterize compound **2**, another SMARCA2/4 degrader from the same patent (Fig. 4a and Extended Data Fig. 5a; WO2023018648)^36^. In contrast to **1**, here, the azepane group was changed to a piperazine and an extra carbon was added to the linker. To determine E3 ligase substrate receptor preference of **2**, we conducted another FACS-based CRISPR–Cas9 screen with the SMARCA2 BD stability reporter, which revealed a complete switch in driver ligase preference to CRL1^FBXO22^ compared to **1** (Fig. 4b and Supplementary Table 2). Moreover, unbiased BioID proximity labeling revealed that FBXO22 was among the few proteins significantly enriched upon treatment with **2** (Fig. 4c and Supplementary Table 3). The preferential proximity between SMARCA2 BD and FBXO22 induced by **2** was further validated by BioID labeling coupled to western blotting (Extended Data Fig. 5b). We then confirmed that *FBXO22* single KO is sufficient to abrogate SMARCA4 ubiquitination and SMARCA2 degradation induced by **2** (Fig. 4d,e). Lastly, the driver ligase switch was confirmed by SMARCA4–HiBiT degradation assays in both dose–response (Fig. 4f) and time-course experiments (Fig. 4g), showing DCAF16 dependence only at higher concentrations. Together, these findings demonstrate that subtle structural modifications to the degradation tail are sufficient to reprogram ligase engagement from CRL4^DCAF16^ to CRL1^FBXO22^, highlighting a chemically tunable mechanism for modulating E3 ligase selectivity in monovalent degraders.

Given that **1** engages DCAF16 through a covalent mechanism, we asked whether the same might be true for the driver ligase of **2**, FBXO22. Therefore, we substituted surface-exposed cysteine residues of FBXO22 and measured their impact on **2-**induced SMARCA2 degradation. Substitutions of C228 and C326 both abolished SMARCA2 degradation induced by **2** (Extended Data Fig. 5c), highlighting a set of previously tractable cysteines in FBXO22 used for TPD^26,42^. Compound **1** was also shown to rely on the same set of cysteines to induce SMARCA2 degradation (Extended Data Fig. 5d) and enable ternary complex formation through TR-FRET (Extended Data Fig. 5e), suggesting a shared mechanism of FBXO22 engagement between both compounds.

**Extended Data Fig. 6:**
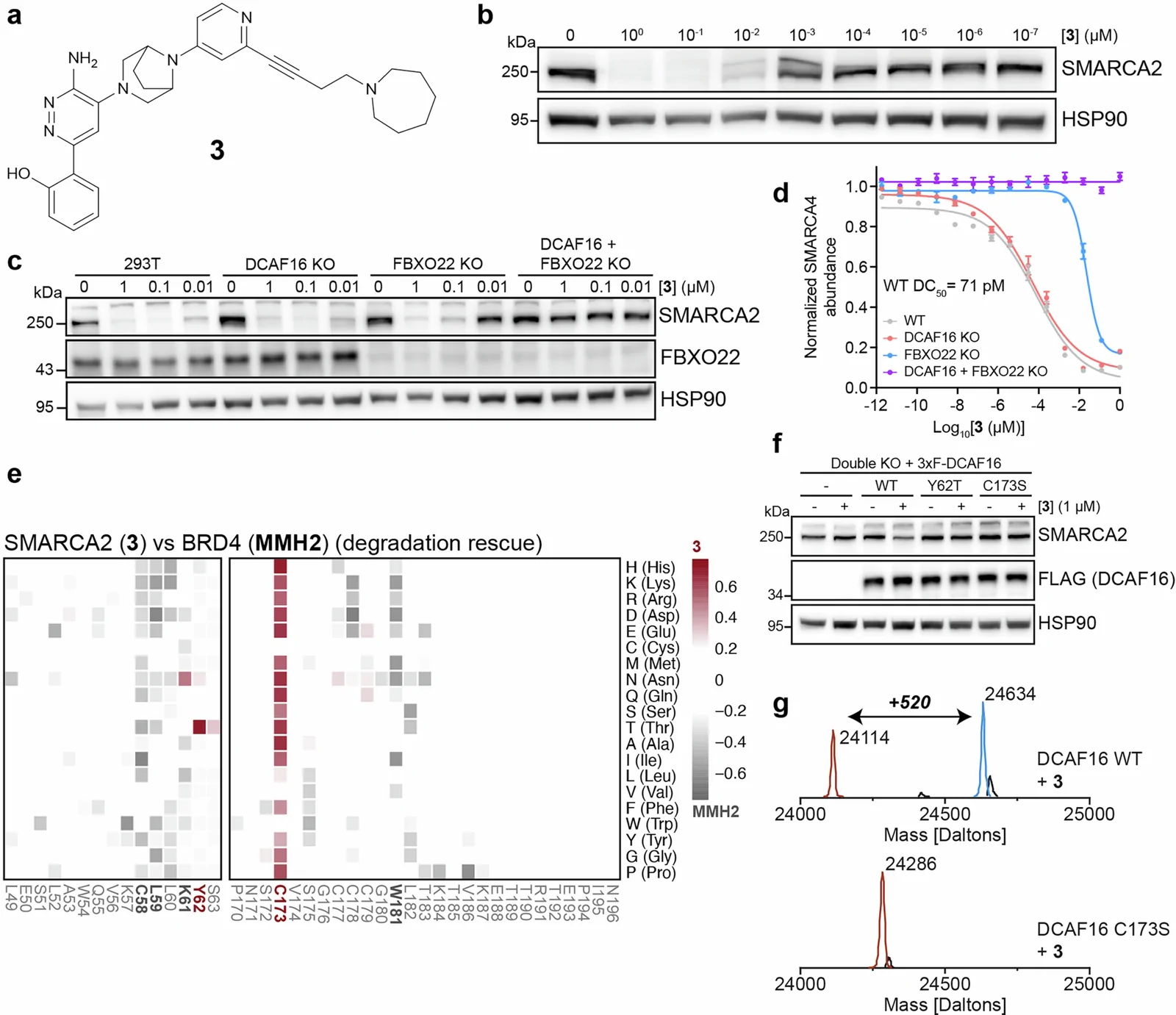
Compounds 3 also exhibits a driver ligase-switch and covalently interacts with DCAF16 via C173. **a**, Structure of Compound 3 (**3**). **b**, Degradation of SMARCA2 by **3**. HEK293T cells treated with a dilution series of **3** for 24 h. **c**, Genetic rescue of SMARCA2 degradation. HEK293T cells with *DCAF16* and *FBXO22* KOs treated with DMSO or increasing concentrations of **3** for 24 hours. **d**, HiBiT endpoint degradation assay. SMARCA4-HiBiT cells treated with a serial dilution of **3** for 16 hours. Data represent mean ± SEM, n = 6 independent replicates. **e**, Deep mutational scanning (DMS) of DCAF16 for degradation rescue. *DCAF16* and *FBXO22* double-KO HEK293T cells stably expressing stability reporters for SMARCA2 BD or BRD4 tandem-BDs were transduced with a library of EGFP tagged DCAF16 variants containing single amino acid substitutions at every position. SMARCA2 and BRD4 stability reporter cells were treated with **3** (1 µM) and MMH2 (1 µM) for 24 hours, respectively. Heatmap depicting differential log_2_ fold enrichment of DCAF16 mutations normalized to maximum log_2_ fold changes versus unsorted control between SMARCA2 targeting **3** and BRD4 targeting MMH2. Data corresponds to SMARCA2^High^ and BRD4^High^ sorted populations, showing mutations that specifically prevent SMARCA2 degradation (red) and BRD4 degradation (gray). n = 3 independent replicates. **f**, Validation of deep mutational scanning. Double-KO HEK293T cells stably expressing 3xFlag tagged DCAF16 variants were treated with **3** (1 µM) for 24 hours. **g**, Intact protein mass spectra for WT (top) or C173S mutant (bottom) DCAF16 (10 µM) and **3** (20 µM). Red peaks represent unmodified DCAF16 and blue peaks represent covalently modified DCAF16. Covalent adduct formation of DCAF16-**3** is shown by the arrow on the top panel. Source data

The same patent and a recent publication also described a potent FBXO22-dependent degrader, G-6599, hereafter referred to as compound **3** (Extended Data Fig. 6a, b)^43^. Compound **3** is almost identical to **1** and only features an additional methylene at the linker between the alkyne and azepane groups. Consistent with **1**, a simultaneous KO of *DCAF16* and *FBXO22* was needed for full rescue of SMARCA2/4 degradation (Extended Data Fig. 6c,d), suggesting that **3** is also dual ligase dependent. Similar to **2**, we observed that **3** also undergoes a driver ligase switch to become more CRL1^FBXO22^ dependent (Extended Data Fig. 6d). To understand whether the driver ligase switch observed with **3** alters the molecular recognition of DCAF16, we turned to our DMS screening data. Consistent with **1**, C173 and Y62T substitutions resulted in the greatest loss of SMARCA2 degradation after treatment with **3** (Extended Data Fig. 6e,f and Supplementary Table 4). We confirmed C173 is also covalently labeled by **3** using intact protein MS, where WT DCAF16 was labeled in the presence of **3**, unlike the DCAF16-C173S mutant (Extended Data Fig. 6g). These findings illustrate how the most minimal of chemical modifications, such as the addition of a single methylene group, can be sufficient to induce a switch in primary ligase dependency.

**Extended Data Fig. 7:**
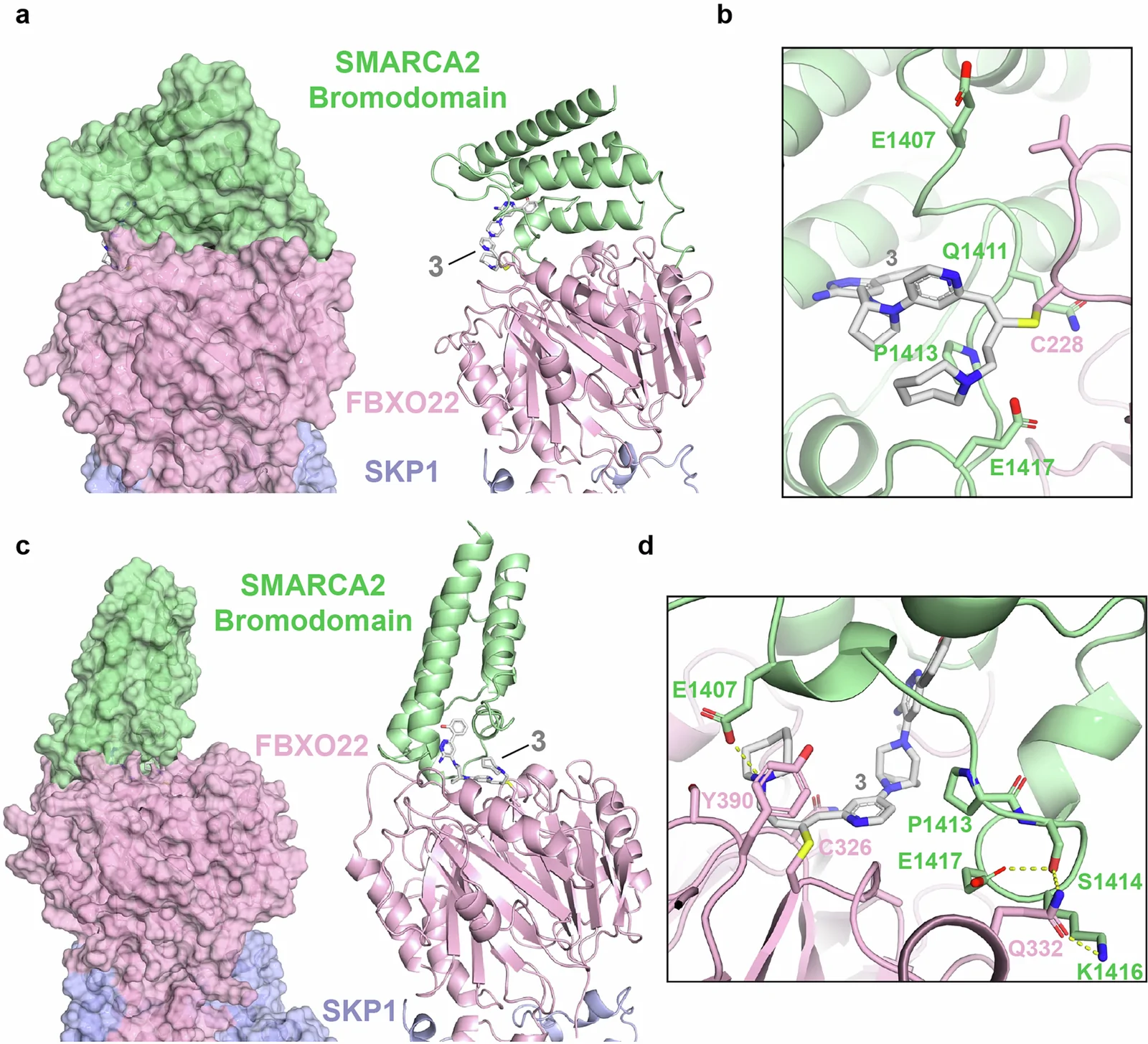
Structural modelling of the ternary complex formed between compound 3, SMARCA2, and FBXO22. **a**, Predicted structure of the FBXO22 (pink)-Skp1 (purple)-SMARCA2 BD (green) ternary complex stabilised by **3** (grey), obtained from molecular dynamics simulations using the Desmond Molecular Dynamics System. The simulation was anchored on a preconditioned covalent adduction of **3** at FBXO22 C228. A representative snapshot from the simulation is shown (left), together with the corresponding atomic model (right). **b**, Close-up view of the region modelled around C228 of FBXO22, which is positioned to be covalently engaged by **3**. **c**, Predicted structure of the FBXO22-Skp1-SMARCA2 BD ternary complex stabilised by **3**, obtained from molecular dynamics simulations using the Desmond Molecular Dynamics System. The simulation was anchored on a preconditioned covalent adduction of **3** at FBXO22 C326. A representative snapshot from the simulation is shown (left), together with the corresponding atomic model (right). **d**, Close-up view of the region modelled around C326 of FBXO22, which is positioned to be covalently engaged by **3**.

Given that all three degraders induce ternary complex formation between FBXO22 and SMARCA2/4, we sought to investigate the underlying structural determinants. We developed predicted structure models for compound **3**, which primarily drives degradation through FBXO22. This approach was informed by prior evidence indicating the roles of C228 and C326 of FBXO22 in the mechanism of action^43^. Two distinct ternary complex models were generated to illustrate how adduct formation at these two sites impact interactions. In the first model (Extended Data Fig. 7a,b), **3** is constrained to form a covalent bond with FBXO22 at C228. In the second model (Extended Data Fig. 7c,d), the covalent bond is instead fixed at C326, leading to more extended protein–protein interactions between SMARCA2 and FBXO22 at their interface. The distinct conformations that the SMARCA2 BD may adapt on FBXO22, combined with reactivity at these two cysteine sites, underscore FBXO22’s potential plasticity in accommodating various degrader targets. This plasticity may help explain the tendency of these degraders to shift dependency toward FBXO22 in response to subtle chemical modifications.

**Extended Data Fig. 8:**
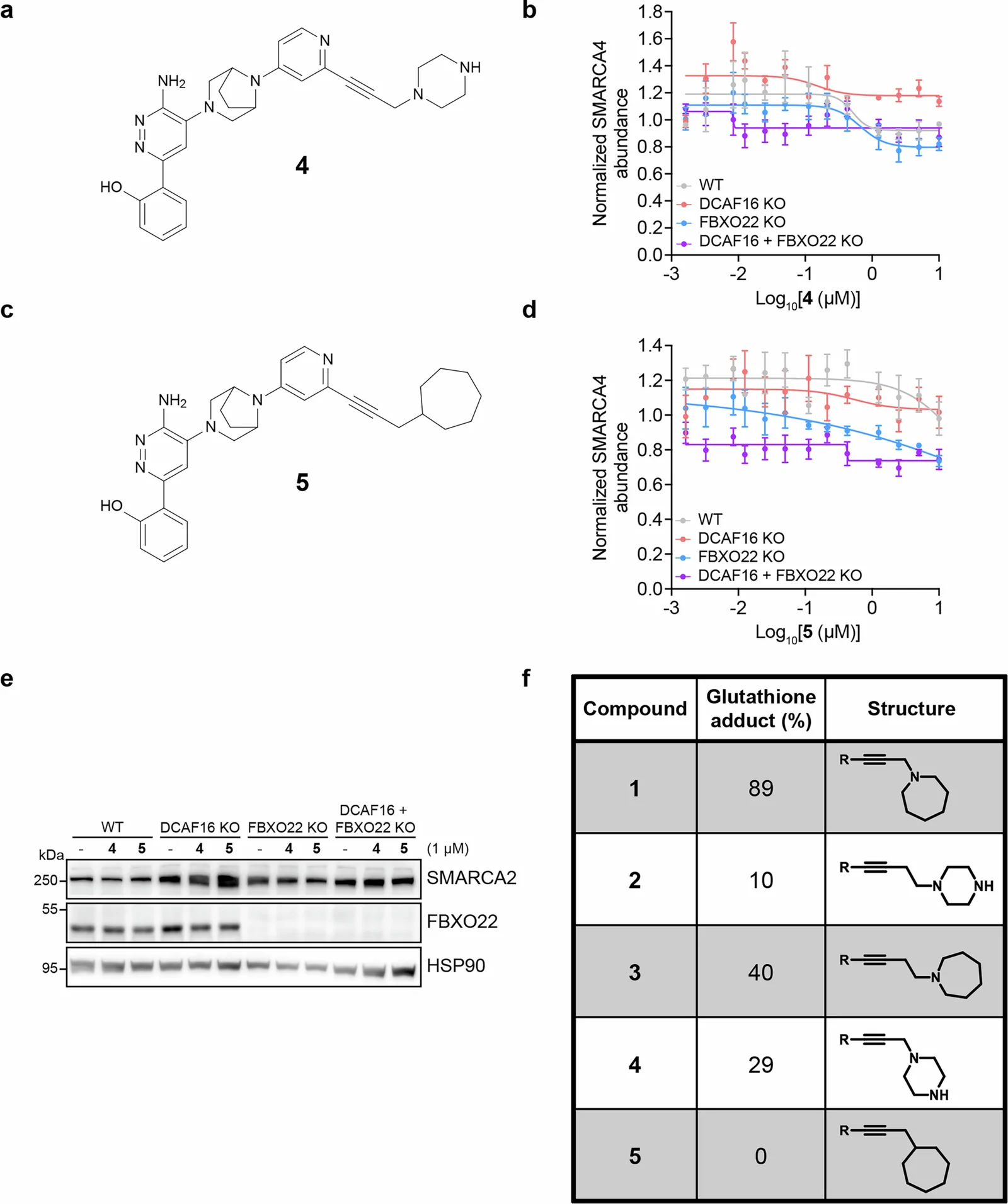
Compounds 4 and 5 do not degrade SMARCA2/4. **a**, Structure of Compound 4 (**4**). **b**, HiBiT endpoint degradation assay. SMARCA4-HiBiT cells treated with a dilution series of **4** for 24 hours. **c**, Structure of Compound 5 (**5**). **d**, HiBiT endpoint degradation assay. SMARCA4-HiBiT cells treated with a serial dilution of **5** for 24 hours. **e**, Genetic rescue of SMARCA2 degradation. HEK293T cells with *DCAF16* and *FBXO22* KOs treated with DMSO or increasing concentrations of **4** or **5** for 24 hours. **f**, Glutathione reactivity assay. The relative reactivity of SMARCA2/4 degraders was assessed using a 3-hour incubation with glutathione at 37 °C. Reactivity was estimated as the UV area ratio of the glutathione adduct to the starting material (%), without external calibration. For **b**, **d**, n = 3 independent replicates. Data represent mean ± SEM for **b**, **d**. Source data

To further explore features in the degradation tail critical for ligase engagement and degrader activity, we synthesized and evaluated a set of analogs of **1**. Modification of the azepane ring on **1** to either the smaller and less basic piperazine (**4**) or a cycloheptane (**5**) resulted in a complete loss of SMARCA2/4 degradation in HiBiT assays and western blotting (Extended Data Fig. 8a–e), highlighting that subtle changes in ring size or basicity profile can disrupt degrader function. We used a glutathione reactivity assay to assess whether the loss in activity correlated with changes in electrophilicity. Compound **1** showed the highest reactivity, followed by **3, 4** and **2**, whereas **5** displayed no detectable reactivity (Extended Data Fig. 8f). These findings emphasize the critical role of the azepane moiety and linker length in maintaining both the chemical reactivity and structural features necessary for selective engagement of DCAF16 and highlight how small modifications can disrupt covalent binding and degradability.

### Genetic tunability

**Fig. 5:**
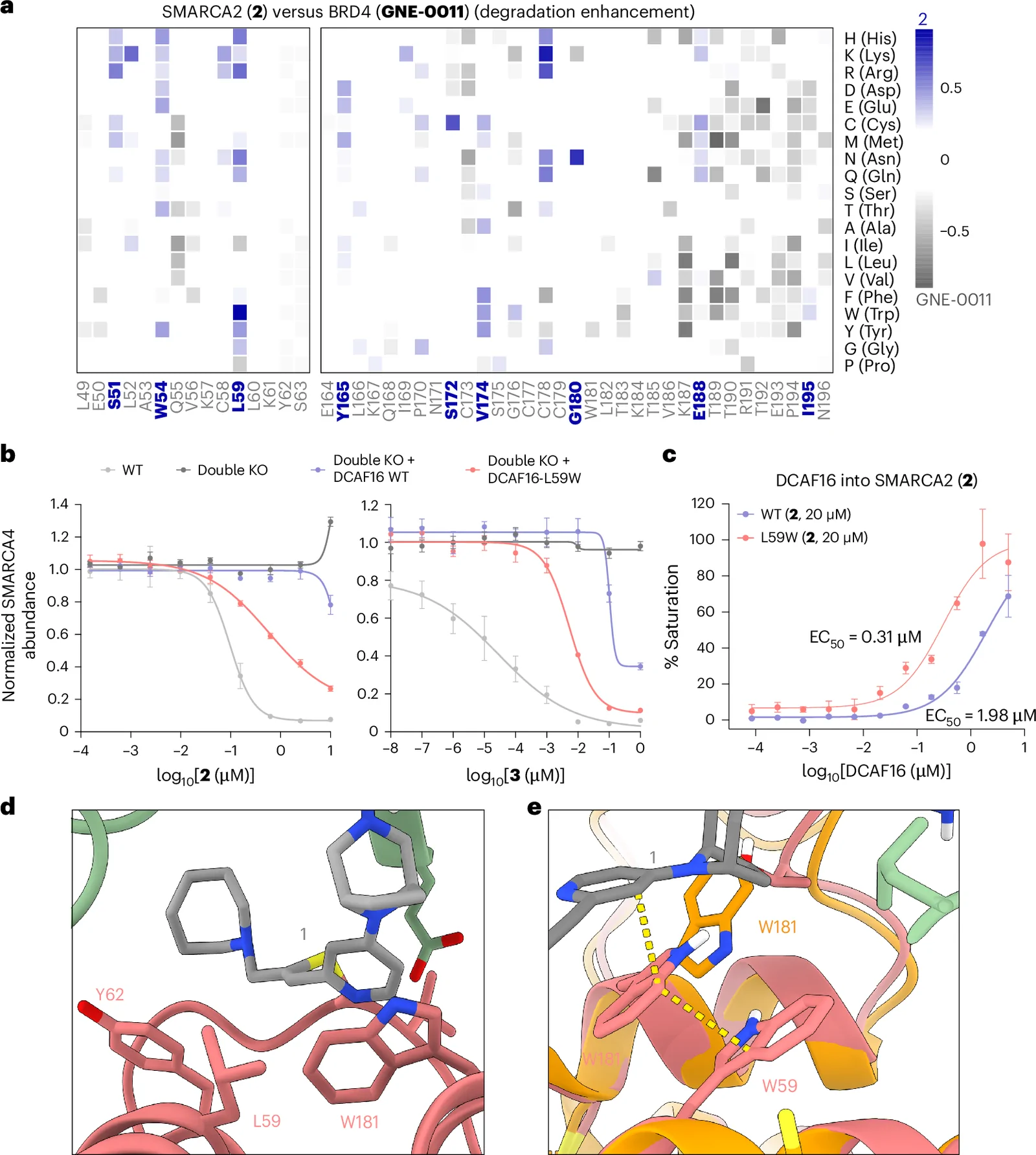
DCAF16 L59W substitution genetically fine-tunes E3 ligase dependency of compounds 2 and 3. **a**, DMS of DCAF16 for degradation enhancement. *DCAF16* and *FBXO22* double-KO HEK293T cells stably expressing stability reporters for SMARCA2 BD or BRD4 tandem BDs were transduced with a library of EGFP tagged DCAF16 variants containing single-amino-acid substitutions at every position. SMARCA2 and BRD4 stability reporter cells were treated with **2** (1 µM) and GNE-0011 (0.1 µM) for 24 h, respectively. Heat map depicting differential log_2_ fold enrichment of DCAF16 substitutions normalized to maximum log_2_FC versus unsorted control between SMARCA2 targeting **2** and BRD4 targeting GNE-0011. Data correspond to sorted SMARCA2^low^ and BRD4^low^ populations, showing substitutions that specifically enhance SMARCA2 degradation (blue) and BRD4 degradation (gray). **b**, DMS validation HiBiT endpoint degradation assay. Double-KO SMARCA4–HiBiT cell lines stably expressing 3×Flag tagged DCAF16 variants treated with a dilution series of **2** for 24 h (left) or **3** for 18 h (right). **c**, TR-FRET ternary complex formation assay. Anti-GST–europium bound to GST–SMARCA2 BD was incubated with either 20 µM of **2** or DMSO and increasing concentrations of DCAF16 (WT or L59W)–DDB1(ΔBPB)–DDA1. Signals obtained in the presence of **2** were background-subtracted using the corresponding DMSO control and subsequently normalized to the top signal value, determined by nonlinear regression, to represent the percentage of saturation. **d**, Position of L59 of DCAF16 in the ternary complex. **e**, Model of the L59W substitution stabilizing W181 that exhibits *π* stacking with the pyridine of **1**. The L59W model with **1** is overlaid with another DCAF16 structure (PDB 8G46; orange) where W181 is flipped outward. Data shown are from *n* = 3 independent replicates (**a**,**c**), or *n* = 6 independent replicates (**b**). Data represent the mean ± s.e.m. (**b**,**c**). Source data

**Extended Data Fig. 9:**
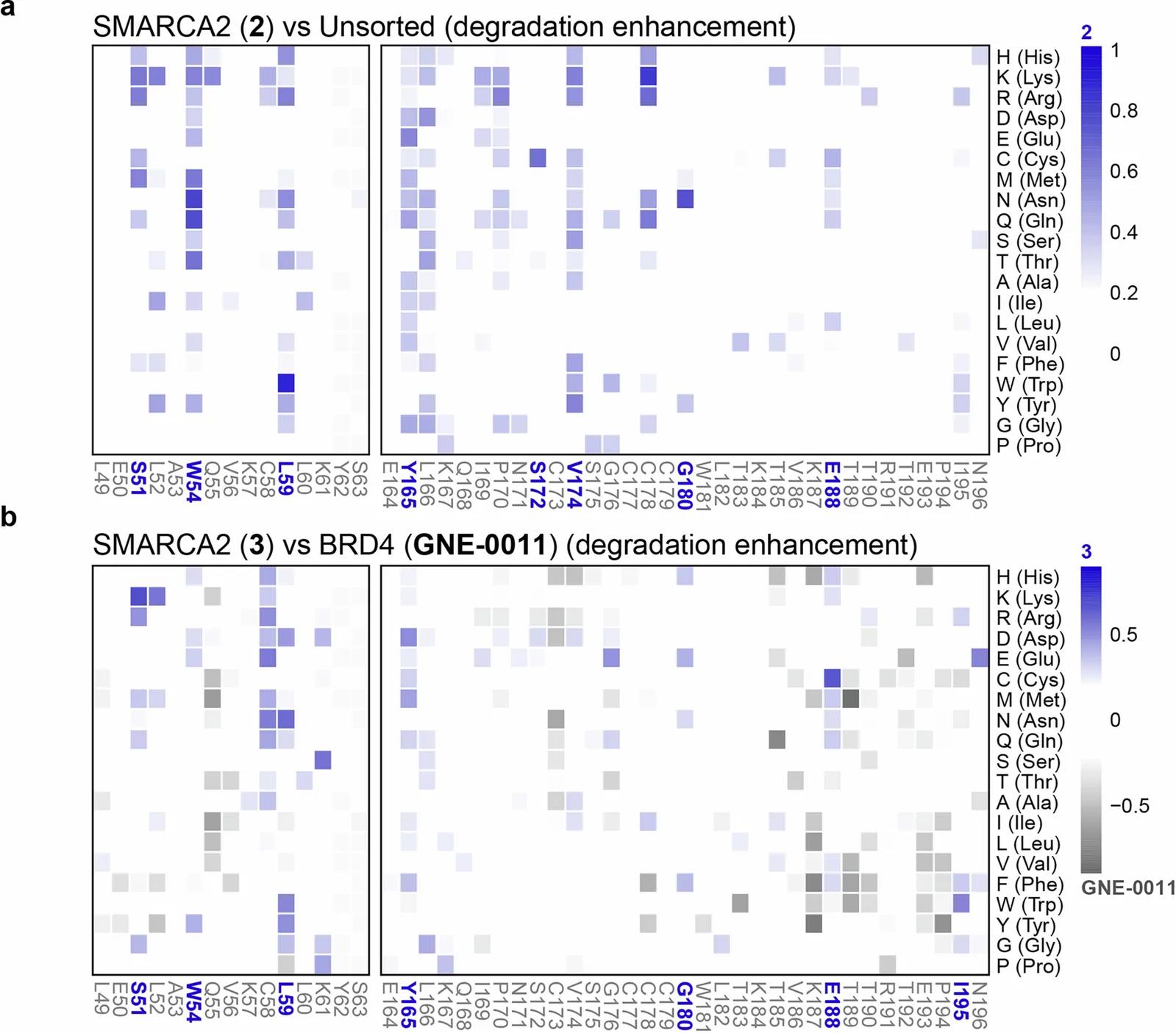
Mutations in DCAF16 that enhance degradation of SMARCA2 via compounds 2 and 3. **a, b**, Deep mutational scanning (DMS) of DCAF16 for degradation enhancement. *DCAF16* and *FBXO22* double-knockout HEK293T cells stably expressing stability reporters for SMARCA2 BD or BRD4 tandem-BDs were transduced with a library of EGFP tagged DCAF16 variants containing single amino acid substitutions at every position. SMARCA2 stability reporter cells were treated with **2** (1 µM) or **3** (1 µM), and BRD4 stability reporter cells were treated with GNE-0011 (0.1 µM) for 24 hours. Heatmap depicting mean log_2_ fold enrichment of DCAF16 mutations normalized to maximum log_2_ fold changes for **2** versus unsorted control (**a**). Heatmap depicting differential log_2_ fold enrichment of DCAF16 mutations normalized to maximum log_2_ fold changes versus unsorted control between SMARCA2 targeting compound **3** and BRD4 targeting GNE-0011 (**b**). Data corresponds to SMARCA2^Low^ and BRD4^Low^ sorted populations, showing mutations that specifically enhance SMARCA2 degradation (blue) and BRD4 degradation (gray). n = 3 independent replicates. Source data

**Extended Data Fig. 10:**
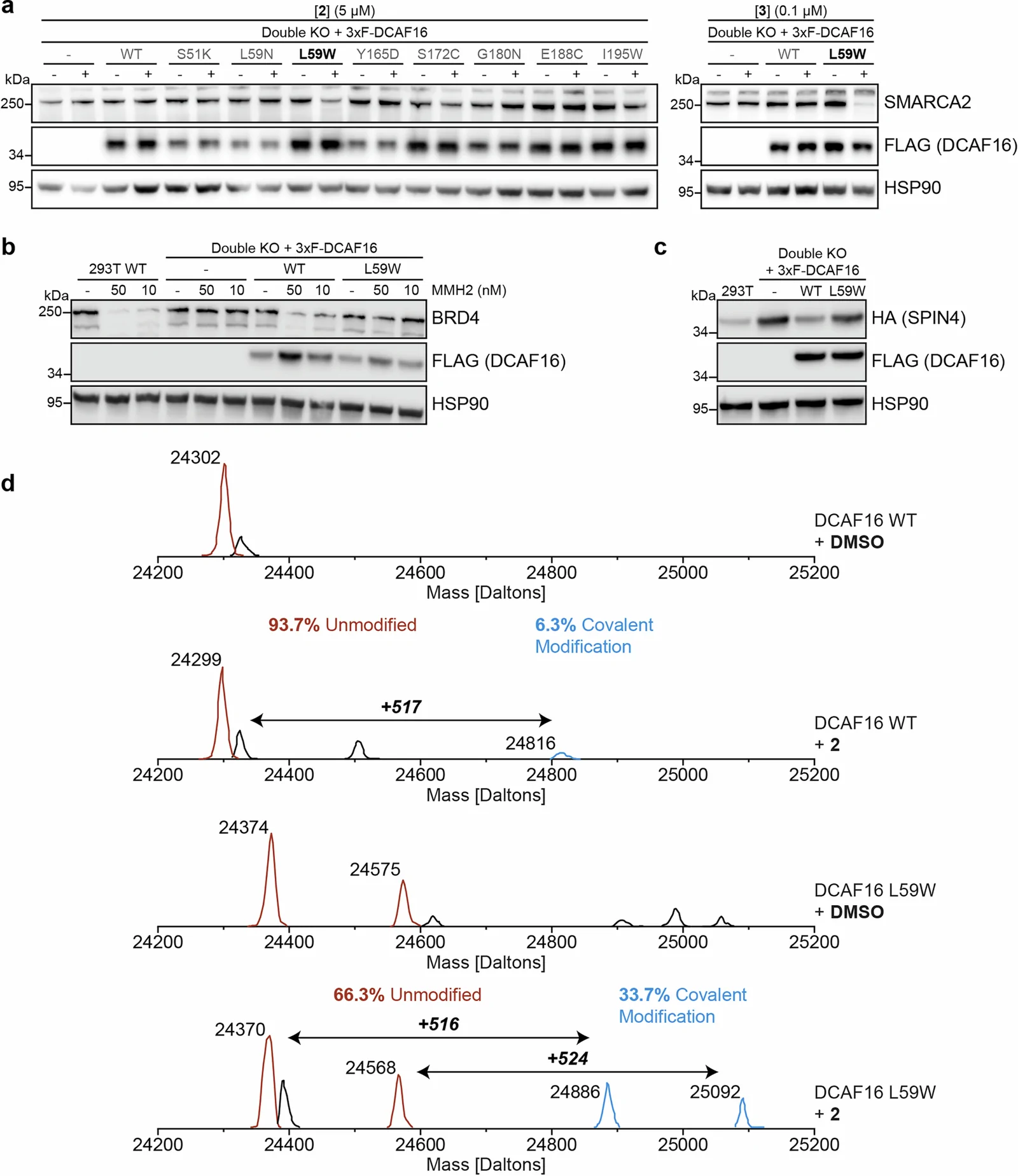
DCAF16 L59W mutation promotes degradation of SMARCA2 via compounds 2 and 3. **a**, Validation of DMS. Double-KO HEK293T cells stably expressing 3xFlag tagged DCAF16 variants were treated with **2** (5 µM) (left) or **3** (0.1 µM) (right) for 24 hours. Left and right represent separate Western blots. **b**, Effect of DCAF16 L59W mutation on BRD4 degradation. Double-KO HEK293T cells stably expressing 3xFlag tagged DCAF16 variants were treated with MMH2 (50 nM, 10 nM) or DMSO for 16 hours. **c**, Effect of DCAF16 L59W mutation on SPIN4 degradation. 2xHA-SPIN4 was stably expressed in double-KO HEK293T cells stably expressing 3xFlag tagged DCAF16 variants. **d**, Intact protein mass spectrometry analysis of DCAF16 labelling by **2**. Intact mass spectra of DCAF16 (WT or L59W)-DDB1(ΔBPB)-DDA1 following co-incubation with **2** or DMSO. All incubations were performed with 10 µM DCAF16–DDB1(ΔBPB)–DDA1 and 20 µM of **2** at room temperature for 18 h. Red peaks represent unmodified DCAF16 and blue peaks represent covalently modified DCAF16. Covalent adduct formation of DCAF16-**2** is shown by the arrows in the second and fourth panels. Source data

After having established that chemical modifications offer a means to fine-tune ligase selectivity, we next asked whether similar control could be achieved through genetic modulation. We again used FACS-based DCAF16 DMS in double-KO HEK293T cells to genetically adapt the topology of the DCAF16 surface, aiming to induce an enhancement or gain of function in SMARCA2 BD degradation by **2** and **3**. To distinguish specific substitutions from those that simply stabilize DCAF16, we concurrently evaluated how substitutions affect BRD4 BD1–BD2 degradation by the DCAF16-dependent degrader GNE-0011. DMS results for **2** and **3** indicated that substitutions at several residues including S51, W54, L59, V174, Y165, G180, E188 and I195 were associated with increased SMARCA2 degradation upon treatment (Fig. 5a, Extended Data Fig. 9a,b and Supplementary Table 4). On the basis of these findings, we expressed the corresponding mutant variants and subsequently examined SMARCA2 degradation using western blot analysis in the presence of **2**. Among the variants tested, L59W emerged as the only substitution capable of unambiguously promoting SMARCA2 degradation in presence of **2** and **3** compared to WT DCAF16 (Extended Data Fig. 10a). Consistently, L59W also caused a marked shift in SMARCA4–HiBiT degradation compared to WT DCAF16 using **2** and **3** (Fig. 5b), illustrating that ligase dependency can be genetically fine-tuned. In contrast, the L59W substitution had the opposite effect on MMH2-induced degradation of BRD4 and degradation of the native DCAF16 substrate (SPIN4)^44^ (Extended Data Fig. 10b,c), abrogating degradation in both cases. This suggests that the gain-of-function degradation-promoting effect with this mutation specifically applies to SMARCA2/4.

To further study the underlying mechanism, the impact of the L59W substitution in the process of ternary complex formation induced by compound **2** was analyzed by TR-FRET. Dose–response analysis revealed that DCAF16-L59W has a substantially lower EC_50_ (0.31 µM) for SMARCA2 engagement compared to WT DCAF16 (1.98 µM) in the presence of **2** (Fig. 5c). In parallel, intact MS was used to assess differences in covalent labeling between variants. Labeling of WT DCAF16 by **2** was limited to 6.3%, whereas labeling increased to 33.7% for the L59W mutant (Extended Data Fig. 10d). Taken together, these data support enhanced compound engagement and ternary complex formation with the L59W mutant. Mechanistically, the role of the L59W substitution could be explained by its proximity to the degraders in the pocket formed by DCAF16 and SMARCA2 BD (Fig. 5d). We noticed that the W181 side chain flips ‘out of plane’ from alternative positions observed in previous DCAF16 structures (PDB 8G46 and 8OV6) to form its *π* stack with the pyridine of **1** (Fig. 5d). From modeling the L59W substitution, we hypothesize that W59 sits below W181, stabilizing the flipped conformation of this residue and forming an extended ‘WW–pyridine shelf’ stacking with the pyridine rings of **2** and **3**, which ultimately allows for increased DCAF16 engagement with these compounds (Fig. 5e).

In sum, these findings demonstrate that, in addition to chemical tuning, ligase choice can be genetically fine-tuned through specific substitutions at the degrader–ligase interface. In particular, the L59W substitution enhances DCAF16 engagement by **2** and **3**, likely by optimizing interactions within the degrader-binding pocket, offering a potential rational approach to enhance DCAF16 recruitment.

## Discussion

Target-focused and ligase-agnostic discovery strategies for small-molecule degraders have been instrumental in expanding the scope of E3 ligases that can be pharmacologically co-opted, circumventing the need to develop new binding ligands for the E3 ligase substrate receptors. Applying such approaches to study SMARCA2/4 degraders featuring a unique propargyl-azepane group at the solvent-exposed exit vector, we identified the first monovalent degraders capable of recruiting two E3 ligase substrate receptors, DCAF16 and FBXO22, in a parallel and redundant fashion. The described compounds are, hence, distinct from monovalent degraders and bivalent PROTACs described to date, which typically recruit a single E3 ligase substrate receptor for target degradation.

A suite of previous studies have identified the structurally unrelated E3 ligase substrate receptors, DCAF16, FBXO22 and DCAF11, as particularly susceptible to covalent labeling^11,12,22,24–27,34,35,41,45,46^. It has also been shown that, depending on the exit vector decoration of a small-molecule degrader, target protein degradation can be biased to either go through DCAF16 or DCAF11 (refs. ^23,35^). A possible explanation for the susceptibility of these ligases is the cysteine-rich surfaces that may correlate with their native roles, which have thus far largely remained elusive. Previous studies outlined template-assisted covalent modification on DCAF16 for degraders of BRD4 and BRD9 (refs. ^22,34,41^). Our findings indicate that the degraders described in this manuscript do not conform to this model. DCAF16 covalent modification can occur without SMARCA2 orienting the degrader for bond formation. This suggests that the propargyl-azepane degradation tail is sufficient to productively orient the degrader on the DCAF16 surface. We also observed that **1** was the most reactive compound in the glutathione assay followed by **2** and **3** (Extended Data Fig. 8f). The reactivity seems to correlate to DCAF16 dependence in cellulo, suggesting a connection between reactivity and ligase dependence. Further work is needed to fully probe such possible connection between ligase reactivity and ligase specificity.

Previous electrophilic compounds have largely targeted C58 and C178 in more structured or conformationally rigid regions of DCAF16 (refs. ^11,20,34,41,46^). In contrast, the cryo-EM structure of DCAF16–DDB1 (**Δ**BPB)–DDA1, SMARCA2 BD and **1** reported in this manuscript reveals that covalent adduction can also occur at C173, located within a conformationally flexible loop. Notably, a recent study also identified C173 as a site for electrophilic engagement, suggesting that this loop may represent a broader hotspot for DCAF16-targeted covalent degrader design^24^.

Here, we also demonstrate that small changes in the degradation tail can influence E3 ligase preference. Specifically, increasing the linker length by one carbon between the alkyne and azepane (**3**) dialed out contributions from DCAF16, thus resulting in a degrader that relies mostly on FBXO22. Complementing the chemical effort to fine-tune ligase recruitment, we also identify a DCAF16 gain-of-function mutant, L59W. This mutant can synthetically dial in dependency on DCAF16 for **2** and **3**, which otherwise leverage FBXO22 as the driver ligase, by locally fine-tuning key interactions for degrader and neosubstrate recruitment. Collectively, these findings outline how chemical and genetic adaptation of complementary surfaces between E3 ligases and target proteins can inform the design of tunable degraders.

The uncovered dual ligase mechanism has relevant implications as it may enhance the therapeutic efficacy of degraders and potentially overcome resistance mechanisms that arise through E3 ligase mutations or downregulation in cancer cells^47^. We previously showed how resistance mutations concentrate in substrate receptors, CRBN and VHL, particularly in regions crucial for assembling the ternary complex with the degrader and target^29^. Given that loss of E3 ligase function is a key resistance mechanism, we later developed heterotrivalent PROTACs, combining VHL, CRBN and target ligands^33^. The compounds described in this study present an alternative strategy to overcome resistance through monovalent and compact degrader designs. Their ability to chemically toggle between distinct E3 ligase complexes adds a valuable layer of flexibility, enabling modulation of ligase engagement to help bypass or delay resistance mechanisms. As novel chemical modalities continue to emerge, such approaches will be essential for advancing next-generation targeted therapies with greater adaptability and resistance resilience.

## Methods

### Cell culture

HEK293, HEK293T and HCT116 cells, originally sourced from the American Type Culture Collection, were provided by the Medical Research Council (MRC) Protein Phosphorylation and Ubiquitylation Unit (PPU) reagents facility at the University of Dundee. KBM7 iCas9 cells were a gift from J. Zuber (IMP Vienna). HEK293T, HCT-116 and Lenti-X 293T lentiviral packaging cells (Clontech) and Flp-In T-REx-293 (Thermo Fisher Scientific) cells were maintained in high-glucose DMEM (Gibco/Sigma-Aldrich). KBM7 cells were maintained in Iscove’s modified Dulbecco’s medium (IMDM; Sigma-Aldrich). Both DMEM and IMDM were supplemented with 10% FBS and 1% (v/v) penicillin–streptomycin (all supplied by Thermo Fisher Scientific/Sigma-Aldrich). Cell lines were grown in a humidified incubator at 37 °C and 5% CO_2_, routinely tested for *Mycoplasma* contamination and authenticated by short tandem repeat profiling.

### Plasmids and oligonucleotides

Generation of the human CRL-focused sgRNA library used for SMARCA2 stability reporter screens, lentiviral sgRNA expression vector used for *DCAF16* gene KO and viral vectors used for the engineering of inducible Cas9 cell lines was previously described^26,35^. The fluorescent protein stability reporter for SMARCA2 was generated by subcloning the BD (residues 1360–1534) of SMARCA2 from SMARCA2 pDONR223 (a gift from M. Taipale) and inserting it into a pRRL lentiviral vector, fused to a 3×V5 tag and mTagBFP at the C terminus and coupled to mCherry with a P2A self-cleaving peptide for normalization. The same SMARCA2 BD sequence was used to create a fusion construct with a miniTurboID enzyme^48^ at the N terminus, plus a flexible linker and a HiBiT tag. This construct was cloned into the pcDNA5/FRT/TO vector (V652020, Thermo Fisher), generously provided by the R. Hay lab, and verified by sequencing to ensure proper insertion and orientation.

The fluorescent protein stability reporter for BRD4 tandem BDs has been previously described^35^. To generate KO cell lines, *DCAF16* and *FBXO22* sgRNA was cloned into a pSpCas9(BB)-2A-EGFP vector from F. Zhang (PX458; Addgene, 48138). For reconstitution experiments, WT and cysteine mutants of FBXO22 fused to a 2×HA tag at the N terminus in a pLEX lentiviral vector were previously described^26^. Exogenous expression of SPIN4 was achieved using the same N-terminal 2×HA construct. Exogenous expression of DCAF16 was achieved from a pRRL lentiviral vector with a 3×Flag tag at the N terminus, as previously described^35^. Expression clones of DCAF16 mutants were generated from the WT 3×Flag tag vector using the Q5 site-directed mutagenesis (SDM) kit (New England Biolabs, E0552), according to the manufacturer’s instructions. For SDM studies, amino acids 2–216 of DCAF16 were selected for site saturation library design and cloning into a pRRL-SFFV-EGFP-cpHalo-(GSG)x9-DCAF16 backbone by GenScript. Quality control of the library by next-generation sequencing (NGS) was also performed by GenScript and resulted in a library coverage of 99.80% and 534×. For immunoprecipitation experiments, expression plasmids pRK5-HA-DCAF16 and pCMV5 HA-FBXO22 were kindly provided by X. Zhang (Northwestern University) or acquired from the MRC PPU reagents facility at the University of Dundee (DU42092). LgBiT and HaloTag–ubiquitin plasmids were purchased from Promega (N2681 and N2721 respectively). Flp-recombinase expression vector (pOG44) was kindly provided by the R. Hay lab. All plasmids and sgRNAs used in this study are shown in Supplementary Tables 5 and 6. The CRL-focused sgRNA library used for FACS-based CRISPR–Cas9 screens is shown in Supplementary Table 2.

### Lentivirus production and transduction

Semiconfluent Lenti-X cells in 10-cm dishes were cotransfected with 1 µg of the envelope plasmid pMD2.G (Addgene, 12259), 2 µg of packaging plasmid psPAX2 (Addgene, 12260) and 4 µg of the lentiviral plasmid using polyethylenimine (PEI MAX, molecular weight: 40,000; Polysciences). Then, 3 days after transfection, supernatant containing virus was collected and filtered through a 0.45-mm filter. Target cells were infected in the presence of 8 μg ml^−1^ polybrene (szabo scandic, SACSC-134220).

### Generation of monoclonal HEK293T KO cell lines

Semiconfluent HEK293T cells in six-well plates were transfected with 2.5 µg of a pSpCas9(BB)-2A-EGFP plasmid containing either *DCAF16* or *FBXO22* sgRNA (Supplementary Table 6) using Lipofectamine 2000 (Thermo Fisher Scientific, 11668027). Then, 2 days after transfection, single cells expressing EGFP were sorted into 96-well plates and allowed to grow for 2 weeks. Monoclonal cell colonies were verified for *FBXO22* or *DCAF16* KO by western blotting and preventing BRD4 degradation in the presence of IBG3 (ref. ^35^). As described with single-KO cell lines, double-KO clonal cells were generated starting from a clonal population of *DCAF16*-KO cells transfected with an *FBXO22* sgRNA plasmid.

### Generation of SMARCA HiBiT cell lines and KO clones

SMARCA2 and SMARCA4–HiBiT CRISPR–Cas9 knock-in cell lines were generated by ribonucleoprotein (RNP) transfection according to the manufacturer’s instructions for single-stranded DNA oligonucleotides (ssODNs; Integrated DNA Technologies, IDT). In brief, ssODNs served as donor templates, while recombinant SpCas9 Nuclease (IDT) was complexed with target-specific sgRNAs (Supplementary Table 6). For RNP transfection, 1 × 10^6^ HEK293T cells were resuspended in MaxCyte buffer (cytiva) and mixed with SMARCA2/4-specific HiBiT ssODNs and RNPs. The mixture was filled into an electroporation cuvette to a final volume of 25 µl and electroporated with a MaxCyte ExPERT ATx. Following electroporation, cells were immediately transferred to a six-well plate as droplets and allowed to recover at 37 °C and 5% CO_2_ for 20 min. Following recovery, 2 ml of prewarmed DMEM (10% FBS and 1% penicillin–streptomycin) supplemented with Alt-R HDR Enhancer V2 (IDT) was added to the cells. The following day, the medium was exchanged and cells were allowed to expand. Then, 3 days after electroporation, the edited cells pools were analyzed with the HiBiT lytic assay (Promega, N3030) to detect luminescence and the HiBiT blotting system (Promega, N2410) to detect proteins tagged with HiBiT at the correct weight through western blotting. To obtain monoclonal populations, single cells were sorted into 96-well plates and allowed to grow for 2 weeks. Clones were expanded and validated as homozygous SMARCA2/SMARCA4–HiBiT clones by genotyping and by performing a lytic HiBiT degradation assay with the SMARCA-specific PROTAC, ACBI1 (ref. ^49^).

SMARCA4–HiBit HEK293T single-KO cell lines were generated by RNP electroporation using target-specific sgRNA (IDT; Supplementary Table 6) and spCas9 (CAS9PROT, Sigma-Aldrich). After forming the RNP complex through 20 min of incubation at room temperature, 4 × 10^5^ HiBiT–SMARCA4 HEK293T cells were resuspended in Neon NxT Resuspension R Buffer (N10025, Thermo Fisher Scientific) along with the RNP complex and electroporated using a 10-µl Neon electroporation system cuvette tip (Thermo Fisher Scientific). Immediately following electroporation, the cells were placed in prewarmed DMEM supplemented with 10% FBS and 1% (v/v) penicillin–streptomycin. Then, 3 days after electroporation, individual cells were sorted into 96-well plates and allowed to grow for 2 weeks. To confirm the successful KO of the *FBXO22* or *DCAF16* genes, western blotting was used to verify the gene KO and to assess the prevention of BRD4 degradation in the presence of IBG3. Lastly, double-KO clonal cells were generated starting from a clonal population of *FBXO22*-KO cells that were subsequently electroporated with a *DCAF16* sgRNA.

### Western blotting

HEK293T and HCT116 cells were plated in six-well or 12-well plates depending on experimental setup at 0.5 × 10^6^–0.6 × 10^6^ cells per ml. Stock solutions of compounds were prepared in DMSO at a concentration of 10 mM and stored at −80 °C. In all experiments, the medium from cell seeding was exchanged with fresh DMEM containing working dilutions of experimental compounds. For the ubiquitin–proteasome inhibition assay, cells were cotreated for 18 h with **1** and 0.5 µM of TAK243, MLN4924 or carfilzomib.

For cell collection, cells were washed once with PBS before lysis on ice for 20 min with RIPA buffer (150 mM NaCl, 1% Triton X-100, 0.5% sodium deoxycholate, 0.1% SDS and 50 mM Tris-HCl pH 8) supplemented with benzonase (1:1,000; Sigma-Aldrich, 70746-3), HALT EDTA-free protease inhibitor cocktail (1:100; Thermo Fisher Scientific, 78437) and 20 mM DTT. After removal of the insoluble fraction by centrifugation at 15,000*g* at 4 °C for 15 min, supernatants were stored at −80 °C. Protein concentration was determined by bicinchoninic acid (BCA) assay (23225, Thermo Fisher Scientific). For HEK293, lysates were prepared with 4× Bolt LDS sample buffer (Thermo Fisher, B0008), heated at 95 °C for 5 min and then run on Bolt 4–12% Bis–Tris gels (Thermo Fisher Scientific) with Bolt MES SDS running buffer (Thermo Fisher Scientific). For HCT116, 4× NuPAGE LDS sample buffer was added to the cell lysates of (NP0007, Thermo Fisher Scientific) supplemented with 10% DTT and heated at 95 °C for 5 min. Samples (20–30 µg) were analyzed by protein electrophoresis with a NuPAGE MOPS SDS running buffer (Thermo Fisher Scientific). Proteins were transferred to nitrocellulose membranes (GE Healthcare, Amersham Protran supported 0.4 mm NC), blocked for 1 h with 5% milk in Tris-buffered saline with Tween-20 (TBS-T) at room temperature, before incubation with primary antibodies at 4 °C overnight. The following primary antibodies were used: HSP90 (1:1,000; Cell Signaling Technology, 4877), FBXO22 (1:200; Santa Cruz, sc-100736), SMARCA2 (1:1,000; Bethyl Laboratories, A301-015A), SMARCA4 (1:1,000; Bethyl Laboratories, A300-813A), HA epitope (3F10) (1:1,000; Merck, 11867423001), HA epitope (C29F4) (1:1,000; Cell Signaling Technology, 3724), HiBiT (1:1,000; Promega, N7200), IRDye 800CW Streptavidin (1:1,000; Licor, 926-32230), hFABTM rhodamine anti-tubulin (1:5,000; Bio-Rad, 12004165) and Flag (1:1,000; Sigma-Aldrich, F1804-200UG). Following overnight treatment, membranes were washed in TBS-T and incubated with horseradish peroxidase (HRP)-conjugated or fluorescence 800CW secondary antibodies for 1–2 h at room temperature. The following secondary antibodies were used: HRP anti-rabbit IgG (1:5,000; Cell Signaling Technology, 7074), HRP anti-mouse IgG (1:5,000; Cell Signaling Technology, 7076), IRDye 800CW anti-rabbit (1:5,000; Licor, 926-32211), IRDye 800CW anti-mouse (1:5,000; Licor, 926-32210), IRDye 800CW anti-rat (1:5,000; Licor, 926-32219) and HRP anti-mouse IgG (1:5,000; Cell Signaling Technology, 7076). Lastly, membranes were washed again with TBS-T and then imaged on ChemiDoc Touch imaging system (Bio-Rad) operated on Image Lab software (version 2.4.0.03). The biological conclusions of the western blots in this study were confirmed in multiple independent experiments. With the exception of Fig. 1c,e and Extended Data Figs. 1b, 5b and 6b,c, all western blot images are representative of 2–3 independent replicates. In addition, multiple orthogonal assays confirmed biological conclusions observed in western blots.

### HiBiT degradation assays

SMARCA4–HiBiT cells were plated in 384-well or 96-well plates at a density of 3 × 10^3^ or 3 × 10^4^ cells per well, respectively. The following day, 5× stock solutions of compounds were dispensed either manually or with a Labcyte Echo550/555 liquid handler dispenser (Beckman Coulter Life Sciences). Cells were treated for 16, 18 or 24 h, as indicated in the respective figure legends before lysis using the HiBiT lytic assay buffer (Promega, N3030) according to the manufacturer’s instructions. Luminescence in each plate was measured using a PerkinElmer Victor X3 plate reader operated on PerkinElmer 2030 software (version 4.0). Treated wells were normalized to a DMSO-only control and analyzed using GraphPad Prism (version 10.0.3) through fitting of four-parameter nonlinear regression curves for extraction of DC_50_ values.

### NanoBRET ubiquitination and kinetic degradation assay

For NanoBRET ubiquitination assays, 0.8 × 10^6^ SMARCA2/4–HiBiT HEK293T cells were seeded in six-well plates. The following day, 1 µg of LgBiT (Promega, N2681) and 1 µg of HaloTag–Ubiquitin complementary DNA (cDNA; Promega, N2721) were transfected using FuGENE HD (Promega, E2311) at a 3:1 transfection reagent-to-plasmid ratio. After 8 h, cells were trypsinized and resuspended in phenol-red-free OptiMEM (Gibco) supplemented with 4% FBS and seeded in 96-well plates at a density of 0.2 × 10^6^ cells per ml in the presence or absence of 0.1 mM HaloTag NanoBRET 618 ligand (Promega, G9801). Following overnight incubation, experimental compounds were added at the indicated concentrations. After 24 h of compound treatment, NanoBRET Nano-Glo substrate (Promega, N1571) was diluted in OptiMEM and added to cells at a 1× final concentration. The 96-well plates were analyzed using a PHERAstar (BMG Labtech) plate reader operated on PHERAstar software (firmware version 1.33) for NanoBRET ratio metric calculation (donor emission: 460 nm, acceptor emission: 618 nm). Data were processed by subtracting NanoBRET ligand-free controls before plotting the NanoBRET signal normalized to DMSO in GraphPad Prism (version 9.5.1).

Kinetic degradation assays were conducted using HiBiT-tagged SMARCA4 HEK293T cells that were transfected with exogenous LgBiT, following the same protocol as described for the NanoBRET ubiquitination assays. The cells were incubated with Endurazine substrate (1:100; N2570, Promega) for 2.5 h at 37 °C before the addition of 10× concentrations of experimental compounds. Luminescence measurements were taken every 5 min for a duration of 28 h using a GloMAX Discover microplate reader (Promega, software version 4.0.0, firmware version 4.92). The data were normalized to DMSO-only controls and T_0_ and then plotted as the luminescence signal over time using GraphPad Prism (version 9.5.1).

### Generation of 293 Flp-In T-REx pools

A total of 1 × 10^6^ Flp-In T-REx 293 cells (R78007, Thermo Fisher) were cotransfected using Lipofectamine 3000 with 3.6 µg of a Flp-recombinase expression vector (pOG44, Thermo Fisher Scientific) and 0.4 µg of a pcDNA5/FRT/TO plasmid containing a HiBiT–SMARCA2 BD with miniTurbo on the N terminus. Then, 24 h after transfection, polyclonal cell pools were washed, replaced with complete medium and, the following day, selected for 2 weeks with 200 µg ml^−1^ hygromycin B (Thermo Fisher Scientific, 10687010). Cells were expanded and then construct expression and biotinylation activity were induced with 1 µg ml^−1^ doxycycline (D9891, Merck) and biotin (B4501, Merck) to be validated by western blotting as described above.

### BioID of SMARCA2 BD

Flp-In T-REx 293 stable cell line containing miniTurbo–SMARCA2 BD was divided into three treatment conditions, with each condition consisting of three 10-cm dishes at ~70% confluency. Expression of the fusion protein was induced by 1 µg ml^−1^ doxycycline treatment for a total of 20.5 h. Then, 12 h after doxycycline induction, cells were then pretreated with 10 µM MLN4924 neddylation inhibitor (5054770001, Merck) for 30 min, followed by addition of 10 µM **1**, **2** or DMSO for 8 h. Biotin (100 µM final concentration) was added to cells undergoing treatment with compounds or DMSO for 2 h. At the endpoint, the culture medium was removed and cells were collected with PBS by scraping in 15-cm tubes pelleted by centrifugation at 600*g* for at least 5 min and washed for a second time in PBS. BioID was performed as described previously^50,51^. Cells were lysed with RIPA lysis and extraction buffer (25 mM Tris-HCl pH 7.6, 150 mM NaCl, 1% NP-40, 1% sodium deoxycholate and 0.1% SDS) (89900, Thermo Fisher Scientific) supplemented with complete EDTA-free protease inhibitor cocktail (11873580001, Roche) and 1:1,000 benzonase nuclease (70746-3, Sigma-Aldrich). Lysates were incubated at 4 °C for 15 min and then centrifuged at 13,000*g* for 15 min at 4 °C. Protein concentration was determined by BCA assay as described above. Next, 300 µg of protein lysate was incubated with 30 µl of precleared high-capacity streptavidin agarose beads (Pierce, 20359) and the mixture was incubated on a rotating wheel for 12 h at 4 °C. Beads were pelleted by centrifugation at 380*g* for 5 min and then transferred with 1 ml of lysis buffer to a fresh Eppendorf tube. Beads were washed five times with washing buffer (50 mM Tris-HCl and 0.5% NP-40) and eluted with 5% SDS.

Elutes were reduced using a final concentration of 10 mM Pierce DTT (A39255, Thermo Fisher Scientific) dissolved in triethylammonium bicarbonate buffer (TEABC) (T7408, Sigma Aldrich) at 60 °C for 30 min. Then, tubes were incubated to room temperature for 20 min in the dark and diluted to 20 mM iodoacetamide (A39271, Thermo Fisher Scientific) dissolved with TEABC. Afterward, samples were processed with an S-Trap mini spin column (PROTIFI, C02-mini) according to the manufacturer’s instructions. Lastly, tryptic digestion was performed by incubating the processed sample with 10 µg of Pierce trypsin protease MS-grade (90058, Thermo Fisher Scientific) dissolved in 100 µl of 100 mM TEABC overnight at 37 °C. Samples were then eluted from the column and transferred to a fresh Eppendorf tube. Columns were washed first with 0.15% formic acid and then with 50% acetonitrile in 0.15% formic acid and pooled with the eluate. Finally, samples were lyophilized and resuspended in 1% formic acid.

The dried peptides were reconstituted in 1% formic acid and analyzed on an Orbitrap Astral MS instrument connected to a Thermo Fisher Scientific Vanquish Neo ultrahigh-performance liquid chromatography (UHPLC) system. The peptides were enriched on a trap column and separated on an analytical column (Easy-Spray PepMap Neo C18, 2 μm, 75 μm × 150 mm) at 800 nl min^−1^. Chromatographic separation was performed using a gradient elution: 5% buffer A (0.1% formic acid) and 22.5% buffer B (90% acetonitrile and 0.1% formic acid) at 14 min and ramped to 35% at 21 min, followed by a return to 9%. The total run time was 22.6 min. Data acquisition was performed in data-independent acquisition (DIA) mode using the Astral analyzer. The MS data acquired in Orbitrap were operated with a fixed cycle time of 5 ms and with a full scan range of 380–980 *m*/*z* at a resolution of 240,000. The automatic gain control (AGC) was set to 500% and ion injection time is custom. Precursor ion selection width was kept at 4 Th and peptide fragmentation was achieved by higher-energy collisional dissociation (HCD, normalized collision energy 25%). For DIA mode, the scan range used was 150–2,000 *m*/*z*, AGC target was 500%, ion injection was custom and detector was Astral.

### Quantitative proteomics

For unbiased identification of degrader target proteins, 1 × 10^6^ HEK293 cells per condition were treated with DMSO (1:1,000), 1* (1 µM) or **1** (1 µM) for 12 h in biological triplicates. Cells were collected by centrifugation and washed two times in ice-cold PBS; cellular pellets were lysed in 100 µl of RIPA buffer supplemented with benzonase and cOmplete EDTA-free protease inhibitor cocktail. Cell debris was removed by centrifugation at 16,200*g* for 15 min at 4 °C. Supernatant was transferred to fresh tubes and protein concentration determined using the BCA protein assay kit. Next, 50 µg of cell lysate was then diluted to a final 5% SDS concentration and proteins were first reduced at 55 °C for 15 min in DTT (A39255, Thermo Fisher Scientific) dissolved in TEABC (T7408, Sigma-Aldrich) to a final concentration of 5 mM. Proteins were alkylated with to a final 20 mM iodoacetamide (A39271, Thermo Fisher Scientific) dissolved with TEABC in the dark for 10 min at room temperature. Afterward, samples were processed with an S-Trap micro spin column (PROTIFI, C02-micro) according to the manufacturer’s instructions. Lastly, tryptic digestion was performed by incubating the processed sample with 10 µg of Pierce trypsin protease MS-grade (90058, Thermo Fisher Scientific) dissolved in 100 µl of 100 mM TEABC overnight at 37 °C. Samples were then eluted from the column and transferred to a fresh Eppendorf tube. Columns were washed first with 0.15% formic acid and then with 50% acetonitrile in 0.15% formic acid and pooled with the eluate. Finally, samples were lyophilized and resuspended in 1% formic acid. The sample was analyzed on an Orbitrap Ascend Tribrid MS instrument coupled to a Thermo Fisher Scientific Vanquish Neo UHPLC instrument. The peptides were enriched on a trap column and resolved on an analytical column (Easy-Spray PepMap Neo C18, 2 μm, 75 μm × 150 mm) with 800 nl min^−1^. The gradient for separation was used as 2–7% B at 6 min, 7–18% at 89 min, 18–27% at 114 min and 27–35% at 134 min followed by column wash. The total run time used was 155 min. The data were acquired in data-independent acquisition mode in an orbitrap analyzer. The MS data acquired in Orbitrap were operated with standard AGC and auto ion injection time and with a full scan range of 300 to 1,350 *m*/*z* at a resolution of 60,000. For the DIA scan, the precursor mass range used was 400–1,000 *m*/*z*, precursors were isolated in quadrupole with an isolation window 10 Th and peptide fragmentation was achieved by HCD (normalized collision energy 30%). The resolution was set as 15,000.

### MS analysis

Acquired raw files were processed using DIA-NN (version 1.8; https://github.com/vdemichev/DIaNN). Human UniProt was used as the protein sequence database. Two missed cleavages and a maximum of two variable modifications per peptide were allowed (acetylation of protein N termini and oxidation of methionine). Carbamidomethylation of cysteines was set as fixed modification. This data analysis was carried out using library-free analysis mode in DIA-NN with ‘deep learning-based spectra and RTs prediction’ enabled^52^. MBR was enabled. The search results were further processed in Perseus^53^. The data were loaded in Perseus in .txt format. Replicates were grouped on the basis of their annotation to **1**, **2**, 1* or DMSO. The data were log_2_-transformed and missing values were imputed from a normal distribution using the ‘processing → imputation → replace missing values from normal distribution’ function in Perseus. Imputation parameters included a width of 0.3 and a downshift of 1.8 s.d., simulating low-abundance values to replace missing entries. The imputation was applied in ‘whole matrix’ mode. Statistical analysis was performed using Perseus (version 2.0.11.0). Intensity values were log_2_-transformed before analysis. Differentially regulated proteins between conditions were identified using a two-sided permutation-based Student’s *t*-test with 250 randomizations. Results were visualized using volcano plots in GraphPad Prism (version 9.5.1) displaying log_2_ fold change versus −log_10_(*P* value).

### SMARCA2 proximity labeling of DCAF16/FBXO22 with immunoprecipitation

Flp-In T-REx 293 miniTurbo–SMARCA2 BD-expressing cells were seeded in 10-cm dishes with 1.2 × 10^6^ cells. The following day, cells were transfected with 5 μg of pRK5 HA–DCAF16 or pCMV5 HA–FBXO22. Then, 33 h after transfection, miniTurbo–SMARCA2 expression was induced with 1 µg ml^−1^ doxycycline for a total time of 15 h. Afterward, cells were pretreated for 30 min with 5 µM MLN4924, followed by the addition of 1 µM **1** or **2** and 170 µM biotin for 8 and 2 h, respectively. Cells were then washed in PBS and collected by centrifugation. The cell pellet was lysed in RIPA buffer supplemented with benzonase and cOmplete EDTA-free protease inhibitor cocktail. Next, 500 μg of cell lysate were incubated with 4 μg of HA antibody (51064-2-AP, Proteintech) or IgG control (sc-2026, Santa Cruz) overnight at 4 °C. Antibodies were then purified with 25 μl of protein A/G magnetic beads (88802, Thermo Fisher Scientific) for 1 h. Immunoprecipitated proteins were then washed five times in IP washing buffer (50 mM Tris-Hxl + 1% NP40 + 1% SDS) and eluted in 4× LDS sample buffer. Samples were analyzed by immunoblotting.

### Flow-cytometric SMARCA2 reporter assays

KBM7 iCas9 cells were transduced with lentivirus expressing SFFV-SMARCA2 BD-mTagBFP-P2A-mCherry to generate stable reporter cell lines. Cell pools expressing both mTagBFP and mCherry were selected by sorting using a CytoFLEX SRT (Beckman Coulter) cell sorter operated on Beckman Coulter CytExpert SRT (version 1.1.0.10007) software. To quantify the influence of genetic perturbations on compound-induced reporter degradation, stable reporter cell lines were transduced with lentiviral sgRNA (pLenti-U6-sgRNA-IT-EF1αs-THY1.1-P2A-NeoR) and selected with neomycin. Cas9 expression was induced with doxycycline (0.4 µg ml^−1^) for 5 days, followed by 24 h of degrader treatment before flow cytometry analysis on an LSR Fortessa (BD Biosciences) operated on BD FACSDiva software (version 9.0). Data analysis was performed in FlowJo (version 10.10.0). The gating strategy is shown in Supplementary Fig. 3. BFP and mCherry mean fluorescence intensity values from Cas9-expressing cells were first normalized by background subtraction of the respective values from reporter negative cells. SMARCA2 abundance was calculated as the ratio of background subtracted BFP to mCherry mean fluorescence intensity and is displayed normalized to DMSO-treated cells.

### FACS-based CRISPR–Cas9 SMARCA2 stability reporter screens

The SMARCA2 BD stability reporter FACS-based CRISPR–Cas9 screen, library preparation, NGS and data analysis were performed as previously described^54^. In brief, doxycycline-inducible Cas9 KBM7 cells stably expressing SMARCA2 BD-mTagBFP-P2A-mCherry were transduced in the presence of 8 μg ml^−1^ polybrene with a UPS-focused sgRNA library targeting 1,301 ubiquitin-associated human genes, with six sgRNAs per gene at a multiplicity of infection of 0.15 and over 1,000× library representation^26^ (Supplementary Table 2). Cells expressing sgRNAs were selected for 14 days with G418 (1 mg ml^−1^; Sigma-Aldrich, A1720) and then Cas9 expression was induced with doxycycline (0.4 μg ml^−1^; PanReac AppliChem, A2951). Next, 3 days after Cas9 induction, 50 × 10^6^ cells were treated with DMSO, 0.1 μM **1** or 1 μM **2** for 24 h in two biological replicates.

After treatment, cells were washed once with 1× PBS and then incubated and stained with anti-Thy1.1–APC (1:400; BioLegend, 202526), Zombie NIR fixable viability dye (1:1,000; BioLegend, 423105) and human TruStain FcX Fc receptor-blocking solution (1:400; BioLegend, 422301) in FACS buffer (1× PBS, 5% FBS and 1 mM EDTA) for 10 min at 4 °C. Following two washes with FACS buffer, cells were fixed with BD CytoFix fixation buffer (BD Biosciences, 554655) for 45 min at 4 °C, protected from light. Fixed cells were then washed again with FACS buffer and stored overnight in FACS buffer at 4 °C. The following day, cells were strained trough a 35-µm nylon mesh and sorted using a BD FACSAria Fusion (BD Biosciences) operated on BD FACSDiva software (version 8.0.2), equipped with a 70-μm nozzle. Aggregates, dead (Zombie NIR-positive), Cas9-negative (GFP-negative) and sgRNA library-negative (Thy1.1–APC-negative) cells were excluded. The remaining cells were sorted on the basis of their SMARCA2 BD–BFP and mCherry levels into SMARCA2^high^ (~5% of cells), SMARCA2^mid^ (~30%) and SMARCA2^low^ (~5%) fractions, ensuring a minimum library representation of 2,000× per replicate. Gating strategy is shown in Supplementary Fig. 3.

For library preparation, genomic DNA (gDNA) from the sorted cell fractions was isolated by cell lysis (10 mM Tris-HCl, 150 mM NaCl, 10 mM EDTA and 0.1% SDS), proteinase K treatment (New England Biolabs, P8107) and DNAse-free RNAse digestion (Thermo Fisher Scientific). DNA was then purified through two rounds of phenol extraction (Sigma-Aldrich, P4557) and isopropanol precipitation (Sigma-Aldrich, I9516). Isolated gDNA was subjected to a two-step PCR amplification of the sgRNA cassette using AmpliTaq gold polymerase (Thermo Fisher Scientific, 4311818), with sample-specific barcodes introduced during the first PCR and standard Illumina adaptors added in the second PCR. The resulting PCR products were purified using Mag-Bind TotalPure NGS beads (Omega Bio-tek, M1378-00) and the final Illumina libraries were pooled and sequenced on a NovaSeq 6000 platform (Illumina). Screen analysis was performed using the crispr-process-nf Nextflow pipeline (https://github.com/ZuberLab/crispr-process-nf/). Briefly, raw FASTQ files were trimmed with cutadapt (version 4.4) to remove random barcodes and spacer sequences. Demultiplexing was conducted on the basis of sample barcodes. Reads were aligned to the custom UPS sgRNA library using Bowtie2 (version 2.4.5) and guide abundance was quantified with featureCounts (version 2.0.1). Final read count tables were generated and subsequently analyzed using the crispr-mageck-nf workflow (https://github.com/ZuberLab/crispr-mageck-nf/) for downstream statistical analysis. Gene-level enrichment (fold changes and *P* values) was calculated in MAGeCK (version 0.5.9)^55^ by comparing sorted SMARCA2^high^ or SMARCA2^low^ populations against the SMARCA2^mid^ reference population, using median-normalized read counts and replicate-level variance estimation.

### DMS of DCAF16

To generate the lentiviral DCAF16 DMS library comprised of 4,300 DCAF16 single-point mutants fused to EGFP–cpHalo, Lenti-X HEK293T cells were seeded in 10-cm dishes and transfected at approximately 80% confluency with 4 μg of library plasmid, 2 μg of psPAX2 (Addgene, 12260) and 1 μg of pMD2.G (Addgene, 12259) using PEI (PolySciences). Viral supernatant was collected after 72 h and cleared of cellular debris by filtration through a 0.45-µm polyethersulfone filter.

Next, 50 million *FBXO22* and *DCAF16* double-KO HEK293T cells stably expressing SMARCA2 BD-mTagBFP-P2A-mCherry or BRD4-tandem BDs-mTagBFP-P2A-mCherry were transduced in the presence of 8 μg ml^−1^ polybrene with the DCAF16 DMS library virus at a multiplicity of infection of 0.16–0.19, yielding a calculated library representation of over 1,000 cells per variant. Library-transduced cells were selected with 1 μg ml^−1^ puromycin for 7 days. Approximately 20 million cells for each stability reporter were treated with DMSO, 1 μM **1**, 1 μM **2**, 1 μM **3**, 1 μM MMH2 or 0.1 μM GNE-0011 for 24 h in three biological replicates. Following compound treatment, cells were trypsinized, harvested, washed with PBS and then fixed with BD CytoFix fixation buffer (BD Biosciences, 554655) for 40 min at 4 °C in the dark. Fixed cells were washed with PBS and stored in FACS buffer (1× PBS, 5% FBS and 1 mM EDTA) at 4 °C overnight.

The following day, cells were strained through a 35-μm nylon mesh and sorted on a BD FACSAria Fusion (BD Biosciences) operated on BD FACSDiva software (version 8.0.2) and equipped with a 70-μm nozzle. Aggregates, dead, reporter-negative (BFP-negative and mCherry-negative) and library-negative (EGFP-negative) cells were excluded from the sort. The remaining cells were sorted on the basis of their SMARCA2 BD–BFP or BRD4–tandem BDs–BFP and mCherry levels into low and high fractions (~5–15% of cells in each fraction), ensuring a minimum library representation of 1,500× per replicate. The gating strategy is shown in Supplementary Fig. 3. Three replicates of 20 million unsorted cells for each stability reporter were also harvested as controls, directly washed and frozen.

DNA libraries of sorted and unsorted cell pools for NGS were prepared as previously described^54^. In short, gDNA was extracted by cell lysis (10 mM Tris-HCl, 10 mM EDTA, 150 mM NaCl and 0.1% SDS), proteinase K treatment (New England Biolabs, P8107) and DNAse-free RNAse digestion (Thermo Fisher Scientific), followed by two rounds of phenol extraction and isopropanol precipitation. DCAF16 variant cDNAs were amplified by PCR from gDNA using Q5 high-fidelity DNA polymerase (New England Biolabs, M0491L) and the following primers: DCAF16_DMS_seq_F (TGGCACAGGAGGTTCAATG) and DCAF16_DMS_seq_R (TCATAATCCGCAGTTCCAGG). PCR reactions for each sample were pooled and purified using Mag-Bind TotalPure NGS beads (Omega Bio-tek, M1378-00). The library preparation from the amplified DNA was performed using the Tagment DNA TDE1 enzyme and IDT for Illumina unique dual indices (Illumina). Library concentrations were quantified with the Qubit 2.0 fluorometric quantitation system (Life Technologies) and the size distribution was assessed using the 2100 Bioanalyzer instrument (Agilent). For sequencing, samples were diluted and pooled into NGS libraries and sequenced on NovaSeq 6000 instrument (Illumina) following a 100-bp, paired-end recipe.

Raw sequencing reads were converted to FASTQ format with SAMtools (version 1.15.1) and BEDTools (version 2.30.0) using the bamtofastq function. Sequencing reads were trimmed using Trim Galore (version 0.6.6) using nextera and pair modes. Short reads were aligned to the DCAF16 cassette and SAM files were generated using the mem algorithm from the bwa software package (version 0.7.17). SAM files were converted to BAM using SAMtools and mutation calling was performed using the AnalyzeSaturationMutagenesis tool from GATK (version 4.1.8.1). Given the sequencing strategy, >98% of reads corresponded to WT sequences and were filtered out during this step. Next, relative frequencies of variants were calculated for each position and variants that were covered by <1 in 30,000 reads were excluded from further analysis. Read counts for each variant were then normalized to the total read count of each sample and log_2_ fold changes were calculated for sorted SMARCA2/BRD4^high^ or SMARCA2/BRD4^low^ fractions over unsorted pools. To correct for differential drug potency, each variant was then normalized to the maximum log_2_ fold change. For drug comparisons, log_2_ fold changes of SMARCA2/BRD4^high^ or SMARCA2/BRD4^low^ over unsorted pools of each drug were subtracted. Heat maps were generated using pheatmap (version 1.0.12) package in R (version 4.1.0).

### Protein expression and purification

#### DCAF16 and mutants

The coding sequences for full-length DCAF16 with tobacco etch virus (TEV)-cleavable N-terminal His_6_ tags were cloned into a pFastBacDual vector under the control of the *polh* promoter. SDM PCR was used to create DCAF16 mutants (C58S, C58A, L59W, C173S and C173A). Coding sequences for full-length DDB1 or DDB1(ΔBPB) and full-length DDA1 were cloned into a pFastBacDual vector under the control of *polh* and p10 promoters, respectively. Bacmids was generated using the Bac-to-Bac baculovirus expression system (Thermo Fisher Scientific). Baculovirus was generated by adding bacmid (1 µg ml^−1^culture volume) mixed with 2 µg of PEI 25K (Polysciences) per µg of bacmid in 200 µl of PBS and incubated at room temperature for 30 min. The mixture was added to a suspension culture of Sf9 cells at 1 × 10^6^ cells per ml in Sf-900 II SFM (Gibco) and incubated at 27 °C with shaking at 110 rpm. Viral supernatant (P0) was collected after 7 days. For expression in *Trichoplusia* *ni* High Five, cells were grown to densities between 1.5 × 10^6^ and 2 × 10^6^ cells per ml in Express Five SFM (Gibco) supplemented with 18 mM L-glutamine and infected with a total virus volume of 1% per 1 × 10^6^ cells per ml, consisting of equal volumes of DCAF16 and DDB1 + DDA1 baculoviruses. For expression in SF9 cells, 3.5 × 10^6^ cells per ml in SF-900 II SFM (Gibco) and infected with 1% of culture volume of each virus (DCAF16 and DDB1:DDA1). Cells were incubated at 27 °C in 2 L of Erlenmeyer flasks (~500 ml of culture per flask) with shaking at 110 rpm for 72 h. Cells were spun at 1,000*g* for 20 min and supernatant was discarded. Pellets were resuspended in lysis buffer (50 mM HEPES, 500 mM NaCl, 1 mM TCEP pH 7.5, Tween-20 to 1% (v/v), magnesium chloride to 2 mM, benzonase to 1 µg ml^−1^ and cOmplete EDTA-free protease inhibitor cocktail (Roche; two tablets per L of initial culture volume)); the resuspension was frozen and stored at −80 °C. Resuspended pellets were thawed and refrozen twice to help with cell lysis. Cell suspensions were sonicated, and lysates were centrifuged at 66,800*g* for 40 min. Clarified lysate was added to 2 ml of nickel agarose resin per L of culture on a roller at 4 °C for 1 h. The mixture of resin and lysate was centrifuged at 500*g* for 2 min to separate lysate from resin, taking the supernatant each time and washing the resin with wash buffer three times (50 mM HEPES, 500 mM NaCl, 1 mM TCEP and 20 mM imidazole, pH 7.5). Bound protein was eluted with elution buffer (50 mM HEPES, 500 mM NaCl, 1 mM TCEP and 500 mM imidazole, pH 7.5). Eluted protein was added to a dialysis bag (Snakeskin, 3.5-kDa molecular weight cutoff (MWCO); Thermo Fisher Scientific) and TEV protease was added to protein and incubated at 4 °C overnight. The sample was run over nickel agarose resin. Flowthrough and washes were collected and pooled. Protein was buffer-exchanged into ion exchange (IEX) buffer A (50 mM HEPES, 50 mM NaCl and 1 mM TCEP, pH 7.5) through 2 h of dialysis at room temperature with a change of dialysis buffer after 1 h. The sample was then loaded onto a HiTrap Q HP 5-ml column (Cytiva). The column was washed with IEX buffer A and bound protein was eluted with a 0–100% IEX buffer B (50 mM HEPES, 1 M NaCl and 1 mM TCEP, pH 7.5) gradient. Fractions containing protein were pooled, concentrated and run on a 16/600 Superdex 200 pg column or 10/300 Superdex 200 Increase (Cytiva) equilibrated in 20 mM HEPES, 150 mM NaCl and 1 mM TCEP, pH 7.5. Fractions containing the purified protein complex were pooled, concentrated, then aliquoted and flash-frozen in liquid nitrogen for storage at −80 °C.

Before intact protein MS experiments purified DCAF16–DDB1–DDA1 and mutants were dephosphorylated with a 1:40 ratio of *Escherichia* *coli* His–GST–lambda protein phosphatase (made in house) to DCAF16 protein at 4 °C overnight in 20 mM HEPES, 150 mM NaCl and 1 mM TCEP pH 7.5 buffer supplemented with 1 mM MnCl_2_. The next day, nickel resin (Abcam) was incubated with the mixture for 1 h, after which it was spun to sediment resin and recover the supernatant containing dephosphorylated DCAF16 and remove His–GST–lambda protein phosphatase that was immobilized on the resin.

#### SMARCA2

SMARCA2 BD (residues 1376–1590, Δ1403–1420) was PCR-amplified and subcloned into His_12_–SUMO (pRSF-DUET1) using quick ligase with 5′-BamHI and 3′-EcoRI. Plasmid was transformed into *E*. *coli* BL21 (DE3). Cultures were subjected to overnight expression at 18 °C, induced with 0.25 mM IPTG at an optical density of 600 nm (OD_600_) of ~0.8–1. Cells were collected by centrifugation and pellets were resuspended in lysis buffer (50 mM HEPES pH 7.5, 300 mM NaCl, 1 mM TCEP and 10% glycerol, supplemented with 2 mM magnesium chloride, benzonase and cOmplete EDTA-free protease inhibitor cocktail (Roche; one tablet per litre initial culture volume)); the resuspended pellet was frozen and stored at −20 °C. The resuspension was thawed and lysed at 30,000 psi using a CF1 cell disruptor (Constant Systems). The lysate was cleared by centrifugation at 50,400*g* for 30 min at 4 °C. The lysate loaded onto a 5-ml HisTrap HP column (Cytiva) equilibrated in lysis buffer and eluted with an imidazole gradient up to 100% elution buffer (50 mM HEPES, 500 mM NaCl, 0.5 mM TCEP and 500 mM imidazole, pH 7.5). Eluted sample was placed into a dialysis bag (Snakeskin, 3.5-kDa MWCO; Thermo) and ULP1 was added to sample to cleave the His_12_–SUMO tag and the bag was placed into 2 L of lysis buffer to dialyze overnight at 4 °C. SMARCA2 BD was run on a 5-ml HisTrap HP column equilibrated in lysis buffer. The flowthrough and wash containing SMARCA2 BD were pooled and concentrated in Amicon centrifugal filter units (3,000-kDa MWCO; Merck Millipore). The protein was loaded onto a HiLoad 16/600 Superdex 75 pg column (GE LifeSciences) equilibrated in 20 mM HEPES pH 7.5, 150 mM NaCl and 0.5 mM TCEP. Fractions containing pure SMARCA2 BD were confirmed by SDS–PAGE, then pooled, concentrated and aliquoted for storage at −80 °C until use. For GST-tagged SMARCA2 BD (residues 1376–1590, Δ1403–1420), SMARCA2 BD was subcloned into pDEST15 vectors (Invitrogen) and transformed into *E*. *coli* BL21 (DE3). Cultures were subjected to overnight expression at 18 °C, induced with 0.4 mM IPTG at an OD_600_ of ~0.8–1. Cells were collected by centrifugation and pellets were resuspended in lysis buffer (50 mM HEPES pH 7.5, 300 mM NaCl, 1 mM TCEP and 10% glycerol, supplemented with 2 mM magnesium chloride, benzonase and complete EDTA-free protease inhibitor cocktail (Roche; one tablet per L of initial culture volume)); the resuspended pellet was frozen and stored at −20 °C. The resuspension was thawed and lysed at 30,000 psi using a CF1 cell disruptor (Constant Systems). The lysate was cleared by centrifugation at 50,400*g* for 30 min at 4 °C. The lysate loaded onto a 20-ml of glutathione affinity resin (Abcam) equilibrated in lysis buffer and incubated on a roller for 3 h at room temperature. The resin was washed by multiple rounds of adding fresh lysis buffer. Elution buffer (50 mM HEPES pH 7.5, 300 mM NaCl, 1 mM TCEP, 10% glycerol and 25 mM L-glutathione) was added to GST–SMARCA2 BD-bound GST resin and incubated for 3 h on a roller at room temperature. The elution was collected and dialyzed into 50 mM HEPES pH 7.5, 50 mM NaCl, 1 mM TCEP and 10% glycerol, then subjected to a 5-ml Hitrap Q column and eluted on a 50 mM–1 mM NaCl gradient. GST–SMARCA2-containing fractions were loaded onto a HiLoad 16/600 Superdex 75 pg column (GE LifeSciences) equilibrated in 20 mM HEPES pH 7.5, 300 mM NaCl and 0.5 mM TCEP.

#### FBXO22–SKP1

His_10_–SUMO–SKP1–(GGS)×4–FBXO22 was Gibson-assembled into a pRSF-DUET1 plasmid. SDM PCR was used to create FBXO22 mutants (C228A and C326A). Protein expression was performed in BL-21 Rosetta (DE3) *E*. *coli* and overnight expression at 18 °C was induced with 0.25 mM IPTG at an OD_600_ of ~0.8–1. Cells were collected by centrifugation and pellets were resuspended in lysis buffer (25 mM HEPES pH 7.5, 250 mM NaCl, 2.5 mM TCEP and 20 mM imidazole, supplemented with 2 mM magnesium chloride, benzonase and cOmplete EDTA-free protease inhibitor cocktail (Roche, one tablet per L of initial culture volume)); the resuspended pellet was frozen and stored at −20 °C. The resuspension was thawed and lysed at 30,000 psi using a CF1 cell disruptor (Constant Systems). The lysate was cleared by centrifugation at 50,400*g* for 30 min at 4 °C. Clarified lysate was added to 2 ml of nickel agarose resin (Abcam) per L of culture on a roller at 4 °C for 1 h. The mixture of resin and lysate was centrifuged at 500*g* for 2 min to separate lysate from resin, taking the supernatant each time and washing the resin with wash buffer three times (50 mM HEPES, 500 mM NaCl, 1 mM TCEP and 20 mM imidazole, pH 7.5). An additional buffer wash was added with buffer supplemented with MgCl_2_ and ATP to remove heat-shock protein. Washed resin was resuspended in wash buffer and ULP1 was added to cleave the His_10_–SUMO tag and left to incubate overnight at 4 °C. The resin was placed into a Poly-Prep chromatography column (Bio-Rad) and the flowthrough and washes containing cleaved FBXO22-SKP1 were collected and pooled. Pooled protein was added to a dialysis bag (Snakeskin, 3.5-kDa MWCO; Thermo) and dialyzed into IEX buffer A (50 mM HEPES, 100 mM NaCl and 1 mM TCEP, pH 7.5) by 2 h of dialysis at room temperature with a change of dialysis buffer after 1 h. The sample was then loaded onto a HiTrap Q HP 5-ml column (Cytiva). The column was washed with IEX buffer A and bound protein was eluted with a 0–100% IEX buffer B (50 mM HEPES, 1 M NaCl and 1 mM TCEP, pH 7.5) gradient. Fractions containing protein were pooled, concentrated and run on a 10/300 Superdex 200 Increase (Cytiva) column equilibrated in 25 mM HEPES, 150 mM NaCl and 1 mM TCEP (pH 7.5). Fractions containing the purified protein complex were pooled, concentrated, then aliquoted and flash-frozen in liquid nitrogen for storage at −80 °C.

#### BRD4 BD2

BRD4 BD2 (residues 333-460) was expressed in *E*. *coli* BL21(DE3) and purified as described previously^56^. In brief, proteins were purified by nickel affinity chromatography and size-exclusion chromatography (SEC). His_6_ tag cleavage and reverse nickel affinity were performed before SEC for some applications; for others, the tag was left on. Purified proteins in 20 mM HEPES, 150 mM sodium chloride and 1 mM DTT (pH 7.5) were aliquoted, flash-frozen in liquid nitrogen and stored at −80 °C.

### Intact protein MS

Before analysis, 10 µM recombinant human dephosphorylated DCAF16:DDB1:DDA1 (WT, C58S, L59W or C173S) was incubated with 20 µM DMSO or compounds and coincubated with 20 µM recombinant SMARCA2 BD for 18 h at room temperature. Buffer consisted of 25 mM HEPES pH 7.5, 150 mM NaCl and 1 mM TCEP. Samples were injected on a column (ZORBAX 300SB-C3), desalted for 1 min and then eluted to an UHPLC Agilent 1290 Infinity III high-throughput system (Agilent) using a gradient of 10% acetonitrile to 95% in 0.1% TFA and water. The fragmentor voltage was 135 V and the capillary voltage was 4,000 V. The MS instrument acquired full-scan mass spectra (*m*/*z* 600–3,000) on an InfinityLab Pro iQ Series Mass Detect. Mass spectra were deconvoluted using Agilent OpenLab CDS (version 2.8). Labeling efficiency for all intact mass analysis was calculated from relative abundance of the peaks.

#### Cryo-EM sample preparation

Before plunge freezing, 20 µM DDB1(ΔBPB)–DCAF16, 50 µM SMARCA2 BD and 60 µM **1** were incubated at ambient temperature for 1.5 h. The complex was diluted fivefold into 20 mM HEPES, 150 mM NaCl and 1 mM TCEP (pH 7.5). Before application on the grids, lauryl maltose neopentyl glycol (LMNG; Anatrace, NG310) was added to a final concentration of 0.01% (w/v). Then, 300-mesh copper Quantifoil R1.2/1.3 grids were glow-discharged for 60 s under vacuum at 30 mA using a Quorum SC7620 5 min before use. A total of 3.5 μl of the complex with 0.1% LMNG was applied to the carbon side only with final concentrations of 4 µM DDB1(ΔBPB)–DCAF16, 10 µM SMARCA2 BD and 12 µM **1**. A Vitrobot Mark IV (FEI) (4 °C, 100% humidity, wait time = 10 s, blot force = 4, blot time = 4 s) was used for blotting with grade 595 filter paper (Agar Scientific, AG47000) before plunging into liquid ethane.

#### Cryo-EM data acquisition

All grids were clipped and screened at the University of Dundee EM Facility using a Glacios (Thermo Fisher Scientific) operating at 200 kV equipped with a Falcon4i direct electron detector and Selectris energy filter. The dataset for DDB1(ΔBPB)–DCAF16, SMARCA2 BD and **1** was collected at the UK National Electron Bio-Imaging Center (eBIC; Diamond Light Source) under BAG access BI-31827-25 using a Krios-II (m03) operating a Schottky X-FEG at 300 kV and Ametek-Gatan BioQuantum K3 imaging filter with slit width of 20 eV. Data were recorded using single-particle EPU version 3.8 (Thermo Fisher Scientific) with aberration-free image shift at a nominal magnification of ×165,000, calibrated pixel size of 0.513 Å, *C*_2_ aperture of 50 μm and objective aperture of 100 μm. All videos were collected using a Gatan K3 direct electron detector (5,760 × 4,092 pixels) in counting mode over 60 dose fractions, recorded as Tiff LZW non-gain-normalized, with a total dose of 60 e^−^ per Å^2^, exposure time of 1.26 s and dose rate of 15.7 e^−^ per pixel per s. In the first instance, single-particle EPU was used to collect 4,336 videos at 0° alpha tilt. For the high-tilt dataset, alpha tilt was set to 30° in single-particle EPU and 6,550 videos were collected under identical settings.

#### Single-particle cryo-EM data processing

Full videos and corresponding metadata were separately imported to cryoSPARC (version 4.5)^57^ for the 0° tilted and the 30° tilted datasets. Each dataset was subjected to patch motion correction and patch contrast transfer function (CTF) estimation and then manually curated with CTF estimation cut at 5 Å. For the 0° dataset, micrographs were denoised in cryoSPARC using 300 micrographs for training over 1,200 epochs. Unbiased particle picking was carried out with blob picker on denoised micrographs with a circular blob (minimum diameter of 80 Å and maximum diameter of 160 Å). The blob-picked particles were extracted with a box size of 512 pixels, Fourier-cropped to 128 pixels (pixel size = 2.05 Å, 4× binning) and subjected to two-dimensional (2D) classification to remove obvious junk. In the first round, the blob-picked particles were input into ab initio reconstruction with eight classes, followed by heterogeneous refinement. Many classes contained free DDB1(ΔBPB) or distorted volumes but one class did contain the complex of DDB1(ΔBPB)–DCAF16 and SMARCA2 BD. Particles for the complex were subjected to 2D classification to remove obvious junk particles and rebalanced by orientations; a subset of ~8,000 particles were taken for training in TOPAZ (version 0.2.5a)^58^. The trained TOPAZ models were used to extract particles from their respective micrographs, extracting 512 pixels at a pixel size of 2.05 Å. The particles were subjected to ab initio reconstruction with six classes. For the 30° dataset, following manual curation, a template picker using a good volume of the complex from a previous data collection was used and picks were inspected and subjected to 2D classification, where junk and free DDB1(ΔBPB) complexes were removed. Selected particles were taken for training in TOPAZ. The trained TOPAZ models were used to extract particles from their respective micrographs, extracting 512 pixels at a pixel size of 2.05 Å. The particles were subjected to ab initio reconstruction with four classes. Particles for the complex were subjected to 2D classification to remove junk and rebalanced by orientations; a subset of particles were taken for further training in TOPAZ. The particles were subjected to 2D classification, ab initio reconstruction (six classes) and nonuniform refinement of the best class. Particle sets were created from this and a further round of TOPAZ was run, followed by ab initio reconstruction into six classes. At this point, particles from ab initio reconstruction containing the complex were extracted at their full box size of 612 pixels (0.513 Å) in separate extraction jobs. The extracted particles from each dataset were combined for ab initio reconstruction in four classes, followed by heterogeneous refinement. Some junk classes with distorted volumes or incomplete complexes were present but a clear volume of the complex emerged and was taken for nonuniform refinement. From the best ab initio class, duplicate particles were removed. Particles were subjected to global CTF refinement followed by a nonuniform refinement, reconstruction and three-dimensional (3D) classification into five classes. The best class was reconstructed and subjected to further classification (four classes). The best volume from the 3D classification was imported to UCSF ChimeraX (version 1.8), Gaussian-blurred, segmented and resampled; an .mrc volume encompassing SMARCA2 and part of DCAF16 was exported into cryoSPARC. The mask was binarized, soft-padded and dilated in cryoSPARC before local refinement. Final refinement contained 30,549 particles from the 0° dataset and 10,271 particles from the 30° dataset. The full processing workflow, gold-standard Fourier shell correlation curve, local resolution estimation, angular distribution plot and posterior position directional distribution plot are presented in Supplementary Figs. 1 and 2. A summary of the cryo-EM data collection conditions and image processing is shown in Supplementary Table 7.

#### Cryo-EM model building and refinement

DCAF16–DDB1(ΔBPB)–DDA1 was extracted from PDB 8G46 (ref. ^34^) and SMARCA2 BD was derived using the sequence in AlphaFold2 (ref. ^59^) server from ChimeraX. They were manually placed into the map in ChimeraX using the ‘fit in map’ tool, followed by visual inspection of the residues in Coot (version 0.9.8.93). Initial restraints for **1** were generated using a SMILES string with eLBOW (in PHENIX version 1.21-5207)^60^. To help guide proper placement of **1** into density, coordinates from PDB 8QJT (ref. ^61^) were used as it contained a similar SMARCA BD binder. To create a covalent bond, a bespoke restraints file needed to be created and refined in PHENIX (Linux command line, version 1.21.1-5286). After initial fitting, it was apparent that there was density visible at the back of DCAF16 that was previously unresolved in PDB 8G46 (ref. ^34^). Therefore, the chain was built using alanine residues and then replaced with correct side chains. The structure was refined with rounds of model building in Coot and real-space refinement with PHENIX (version 1.20.1-4487). Validation was performed in PHENIX. Figures were generated in ChimeraX and PyMOL (version 3.0.2). The electrostatic potential map was generated using PDB2PQR and the APBS server and the data were visualized in ChimeraX^62^.

#### Cy5 NHS ester labeling

For DCAF16–DDB1–DDA1 and FBXO22–SKP1 labeling, Alexa Fluor 647 Cy5 NHS ester (Thermo Fisher Scientific) in DMF was prepared to a final concentration of 1 mg ml^−^^1^ with a final concentration of DCAF16–DDB1–DDA1/FBXO22–SKP1 at 10 mg ml^−1^ and sodium bicarbonate at 100 mM, diluted in 25 mM HEPES pH 8, 150 mM NaCl and 1 mM TCEP. The solutions were protected from light and incubated for 1 h at room temperature. The solutions were run on a Superdex 200 10/300 GL column (Cytiva) to remove free dye and aggregated protein. Fractions containing the Cy5-labeled protein were pooled and concentrated; the degree of labeling was calculated to be greater than 100% for each batch of labeled protein. Labeled protein was aliquoted, then flash-frozen in liquid nitrogen and stored at −80 °C.

#### TR-FRET proximity assay

Stock solutions of reaction components including Cy5-labeled DCAF16–DDB1–DDA1 (WT, C173S and L59W), Cy5-labeled FBXO22 (WT, C228A and C326A), GST–SMARCA2 BD, compounds and LanthaScreen anti-GST–Europium donor (Thermo Fisher Scientific) were prepared in TR-FRET assay buffer (50 mM HEPES pH 7.5, 150 mM NaCl, 1 mM TCEP and 0.05% Tween-20). In this assay, the compound or protein was titrated to determine ternary complex formation. Compounds were titrated 1:4 into 125 nM GST–SMARCA2 BD and 500 nM Cy5–DCAF16 or Cy5–FBXO22 in a Corning 3820 low-volume 384-well flat-bottom plate (black) to a final well volume of 20 μl. Anti-GST donor and DMSO concentrations were kept constant across the plate for both assay formats at 2 nM and 1%, respectively. Background subtraction was performed with the condition containing Cy5-labeled DCAF16 or FBXO22 and GST–SMARCA2 BD but no compound. For DCAF16(L59W)–DDB1–DDA1 studies, L59W or WT recombinant protein was titrated 1:4 into 125 nM GST–SMARCA2 BD and 20 µM **2** in a Corning 3820 low-volume 384-well flat-bottom plate (black) to a final well volume of 20 μl. Anti-GST donor and DMSO concentrations were kept constant across the plate for both assay formats at 2 nM and 1%, respectively. Background subtraction was performed with the condition containing Cy5-labeled DCAF16 and GST–SMARCA2 BD but no compound. Components were mixed by spinning down plates at 50*g* for 1 min and plates were covered and incubated at room temperature for 30 min. Plates were read on a PHERAstar FS (BMG LABTECH, firmware version 1.33) with fluorescence excitation and dual emission wavelengths of 337 and 620/665 nm, with an integration time between 70 and 400 μs. Data were processed in GraphPad Prism (version 10.4.1); the curve was fitted to a nonlinear fit (log(agonist) versus response) with variable slope (four parameters).

#### Glutathione reactivity assay

A 1 mM solution of L-glutathione (Sigma-Aldrich, G4251) was prepared in DPBS (Gibco, 14287080). Each test compound (2 μl, 10 mM in DMSO) was mixed with 98 μl of the L-glutathione solution and incubated at 37 °C for 3 h. The ultraviolet ratio of the test compound to the glutathione adduct was then measured using LC–MS.

#### Molecular dynamics simulations

The cryo-EM structure of the ternary complex involving **1**, SMARCA2 and DCAF16 was pretreated using the protein preparation workflow (PPW) available in Schrödinger Suite (release 2024-3) and Maestro before initiating the MD simulation experiments. The PPW treatment involved preparing the structure at pH 7.5 by adding hydrogen atoms and building the missing side-chain atoms. The hydrogen-bond network was optimized by reorienting hydroxyl and thiol groups, water molecules, amide groups of asparagine and glutamine and the imidazole ring in histidine and predicting protonation states of histidine, aspartic acid and glutamic acid and tautomeric states of histidine.

Simulations were carried out using the Desmond MD System implemented within the Schrödinger Suite (release 2024-3). The systems for MD simulations were built in an orthorhombic box (with buffer distance of 12 Å) of explicit water molecules (SPC water model). A salt concentration of 0.15 M was maintained and ions (Na^+^, Cl^−^) were placed to neutralize the systems.

The built systems were relaxed before the production run in three sequential steps to relieve strain and optimize geometry. In the first step, all atoms in the proteins and the ligand were constrained. In the second step, only the Cα atoms of the proteins were constrained. In the final step, no positional constraints were applied. This stepwise approach allowed gradual relaxation of the system while maintaining the overall structural integrity.

Production MD simulations were carried out in the NPT ensemble (300 K, 1 bar) using Desmond default settings, Nosé–Hoover chain thermostat (relaxation time: 1 ps), Martyna–Tobias–Klein barostat (2 ps) and a multitimestep RESPA integrator (2 fs for bonded and short-range interactions, 6 fs for long-range interactions). Long-range electrostatics used the u-series decomposition with a 9-Å real-space cutoff. Two independent 500-ns production runs were started from randomized velocities. Coordinates and energies were saved every 500 ps (1,000 frames per run). All analyses reported in this study are based on these production trajectories.

#### FBXO22 models

The structure outputs (**3**, SMARCA2 and FBXO22) from Boltz-2 were pretreated using the PPW available in Schrödinger Suite (release 2024-3). The PPW treatment involved preparing the structure at pH 7.5 by adding hydrogen atoms and building the missing side-chain atoms. The hydrogen-bond network was optimized by reorienting hydroxyl and thiol groups, water molecules, amide groups of asparagine and glutamine and the imidazole ring in histidine and predicting protonation states of histidine, aspartic acid and glutamic acid and tautomeric states of histidine.

Simulations were carried out using the Desmond MD System implemented within the Schrödinger Suite (release 2024-3). The systems for MD simulations were built in an orthorhombic box (with buffer distance of 12 Å) of explicit water molecules (SPC water model). A salt concentration of 0.15 M was maintained and ions (Na^+^, Cl^−^) were placed to neutralize the systems.

The built systems were subjected to a 100-ps energy minimization in three sequential steps to relieve strain and optimize geometry. In the first step, all atoms in the proteins were constrained. In the second step, only the Cα atoms of the proteins were constrained. In the final step, no positional constraints were applied. This stepwise approach allowed gradual relaxation of the system while maintaining the overall structural integrity.

### Reporting summary

Further information on research design is available in the Nature Portfolio Reporting Summary linked to this article.

## Online content

Any methods, additional references, Nature Portfolio reporting summaries, source data, extended data, supplementary information, acknowledgements, peer review information; details of author contributions and competing interests; and statements of data and code availability are available at https://doi.org/10.1038/s41589-026-02224-y.

## Supplementary information

Supplementary Information Chemical synthesis and Supplementary Note, Figs. 1–3 and Tables 5–7.

Reporting Summary

Supplementary Table 1 Quantitative expression proteomics results including gene names and protein descriptions, total and unique peptides counts, log_2_ abundance for each biological replicate, average of log_2_ abundance across replicates, log_2_ fold changes of **1** compared to 1* and *P* values derived from a two-sided permutation-based Student’s *t*-test with 250 randomizations.

Supplementary Table 2 sgRNA library details including guide identifiers, guide sequences and raw read counts for FACS-based SMARCA2 stability CRISPR screens.

Supplementary Table 3 BioID proximity labeling proteomics with SMARCA2 BD results including gene names and protein descriptions, total and unique peptides counts, log_2_ abundance for each biological replicate, average of log_2_ abundance across replicates, log_2_ fold changes of **1** or **2** compared to DMSO and *P* values derived from a two-sided permutation-based Student’s *t*-test with 250 randomizations.

Supplementary Table 4 DCAF16 DMS data with SMARCA2 or BRD4 stability reporters. Datasets show the log_2_ fold enrichment of all possible single-amino-acids substitutions for DCAF16 (excluding stop codon) normalized to maximum log_2_ fold changes for compounds versus the unsorted control. Data for sorted SMARCA2/BRD4^high^ and SMARCA2/BRD4^low^ populations are included, which correspond to degradation rescue and degradation enhancement, respectively.

## Source data

Source Data Fig. 1 Unprocessed western blots.

Source Data Fig. 1 Statistical source data.

Source Data Fig. 2 Unprocessed western blots.

Source Data Fig. 2 Statistical source data.

Source Data Fig. 2 Unmodified intact MS peaks.

Source Data Fig. 4 Unprocessed western blots.

Source Data Fig. 4 Statistical source data.

Source Data Fig. 5 Statistical source data.

Source Extended Data Fig. 1 Unprocessed western blots.

Source Extended Data Fig. 1 Statistical source data.

Source Extended Data Fig. 2 Unprocessed western blots.

Source Extended Data Fig. 2 Statistical source data.

Source Extended Data Fig. 3 Statistical source data.

Source Extended Data Fig. 3 Unmodified intact MS peaks.

Source Extended Data Fig. 5 Unprocessed western blots.

Source Extended Data Fig. 5 Statistical source data.

Source Extended Data Fig. 6 Unprocessed western blots.

Source Extended Data Fig. 6 Statistical source data.

Source Extended Data Fig. 6 Unmodified intact MS peaks.

Source Extended Data Fig. 8 Unprocessed western blots.

Source Extended Data Fig. 8 Statistical source data.

Source Extended Data Fig. 9 Statistical source data.

Source Extended Data Fig. 10 Unprocessed western blots.

Source Extended Data Fig. 10 Unmodified intact MS peaks.

## Data Availability

Additional data for Figs. 1b,d, 2a, 4b,c and 5a and Extended Data Figs. 2c, 3a,b, 6e and 9a,b are included in Supplementary Tables 1–4. Cryo-EM density maps were deposited to the EM Data Bank with accession code EMD-54549. The atomic model was deposited to the Protein Data Bank under accession code 9S3R. Quantitative proteomics data were deposited to the ProteomeXchange Consortium PRIDE repository with accession codes PXD065445 and PXD065523. All biological materials are available upon reasonable request through material transfer agreements with CeTPD (University of Dundee) or CeMM Research Center for Molecular Medicine of the Austrian Academy of Sciences, respectively. Source data are provided with this paper.
