# Supplementary material for: Dual E3 ligase recruitment by monovalent degraders for tunable SMARCA 2/4 degradation
Source: Nat Chem Biol. 2026 May 12;22(10):1666–75. doi: 10.1038/s41589-026-02224-y (PMC13612222; doi:10.1038/s41589-026-02224-y)

EDF 2e

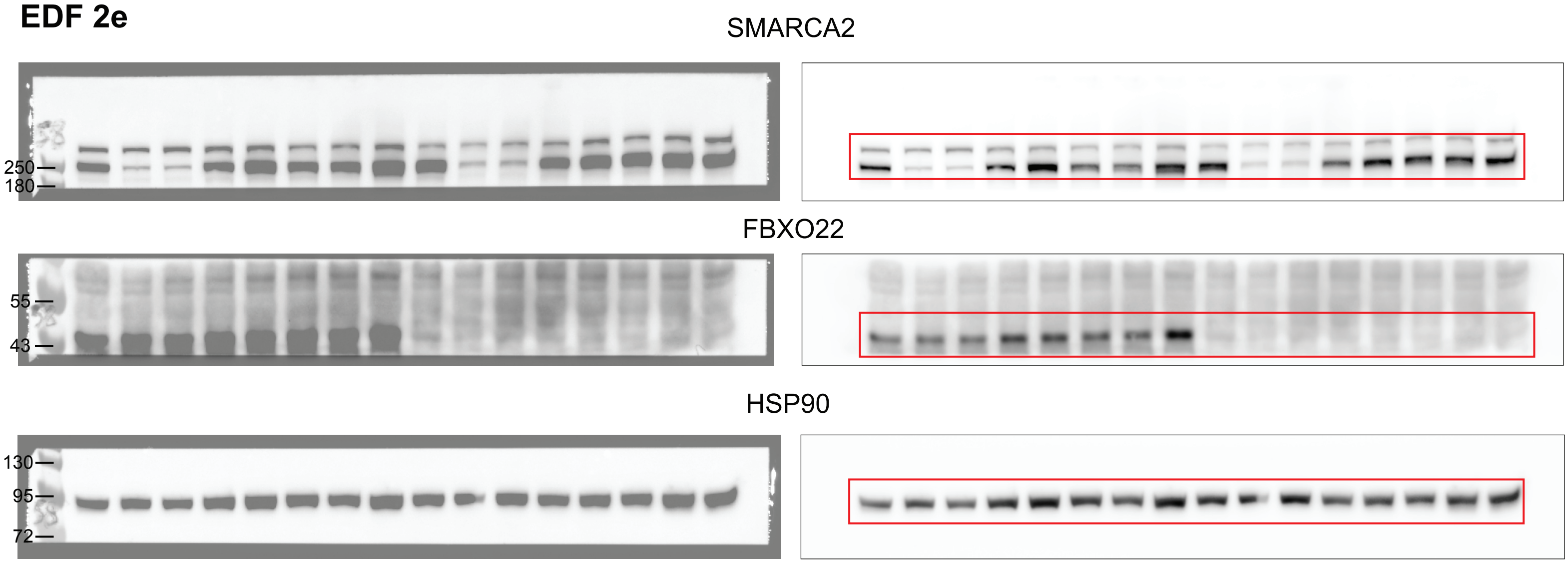

Supplement: Supplementary file 4 [file 41589_2026_2224_MOESM17_ESM.pdf]
