# Supplementary material for: Dual E3 ligase recruitment by monovalent degraders for tunable SMARCA 2/4 degradation
Source: Nat Chem Biol. 2026 May 12;22(10):1666–75. doi: 10.1038/s41589-026-02224-y (PMC13612222; doi:10.1038/s41589-026-02224-y)
Supplement: Supplementary file 5 [file 41589_2026_2224_MOESM1_ESM.pdf]

nature chemical biology

Supplementary information https://doi.org/10.1038/s41589-026-02224-y

Dual E3 ligase recruitment by monovalent degraders for tunable SMARCA 2/4 degradation

In the format provided by the authors and unedited

Supplementary Note: Chemical Synthesis

1. General Information

Chemicals that are commercially available were purchased from Apollo Scientific, Sigma-Aldrich, Fluorochem, CombiBlocks, TCI, and Enamine and were used without further purification. Liquid chromatography–mass spectrometry (LC-MS) was carried out on a Shimadzu HPLC/MS 2020 equipped with a Hypersil Gold column (1.9 μm, 50 × 2.1 mm2), a photodiode array detector, and an electrospray ionization (ESI) detector. The samples were eluted with a 3 min gradient of 5–95% acetonitrile in water containing 0.1% formic acid at a flow rate of 0.8 mL/min. Flash column chromatography was performed on a Teledyne ISCO Combiflash Companion installed with disposable normal phase RediSep Rf columns (230–400 mesh, 40–63 mm; SiliCycle). Preparative HPLC purification was performed on a Gilson preparative HPLC system equipped with a Waters X-Select C18 column (100 mm × 19 mm and 5 μm particle size) using a gradient from 5 to 95% of acetonitrile in water containing 0.1% formic acid over 10 min at a flow rate of 25 mL/min. Compound characterization using NMR was performed either on a Bruker 500 Ultra shield or on a Bruker Ascend 400 spectrometer. The ^{1}H NMR and ^{13}C NMR reference solvents used are $CDCl_3$ (δ_H = 7.26 ppm/δ_C = 77.16 ppm), CD_3OD (δ_H = 3.31 ppm/δ_C = 49.86 ppm), DMSO-*d6* (δ_H = 2.50 ppm/δ_C = 39.52 ppm). Signal patterns are described as singlet (s), doublet (d), triplet (t), quartet (q), multiplet (m), broad singlet (bs), or a combination of the listed splitting patterns. The coupling constants (*J*) are measured in hertz (Hz).

2. Experimental Procedures

2.1. Synthesis of Compound 1*

2-(6-amino-5-(8-(2-bromopyridin-4-yl)-3,8-diazabicyclo[3.2.1]octan-3-yl)pyridazin-3-yl)phenol (1*)

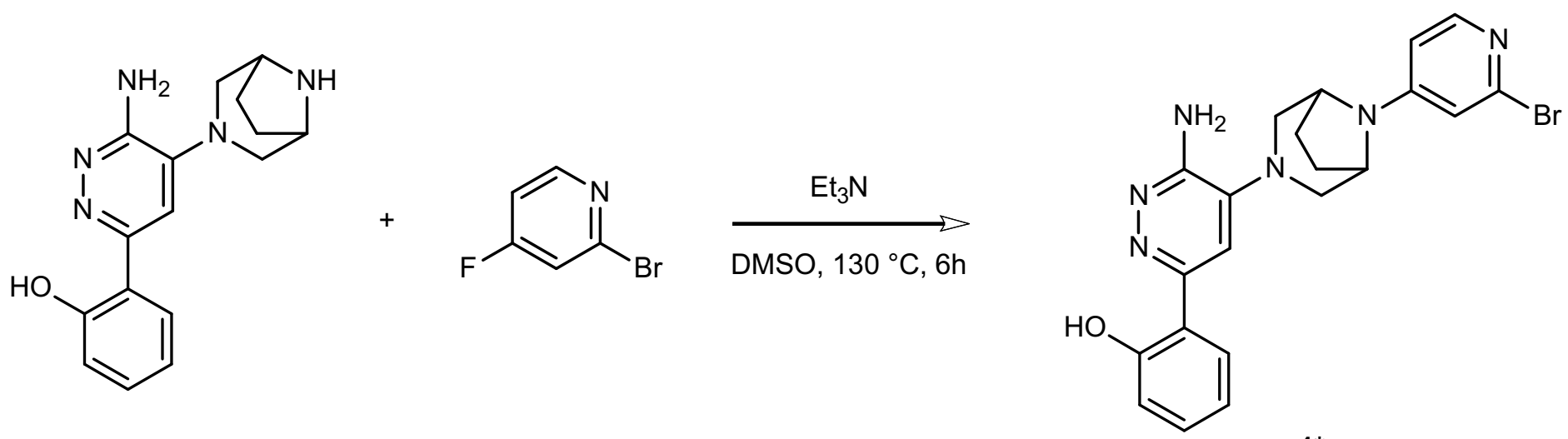

1*

To a solution of 2-(6-amino-5-(3,8-diazabicyclo[3.2.1]octan-3-yl)pyridazin-3-yl)phenol (1.0 g, 3.36 mmol, 1.0 eq.) in DMSO (10 mL) was added 2-bromo-4-fluoro-pyridine (651 mg, 3.7 mmol, 1.1 eq.) and triethylamine (2.3 mL, 16.8 mmol, 5.0 eq.). After being stirred at 130 °C for 6 hrs, the reaction was diluted with EtOAc and water. The mixture was extracted with EtOAc. The organic layer was dried over Na_2SO_4, filtered, and concentrated. The residue was suspended in $CHCl_3$/EtOAc (1/10) and filtered. The solid material was collected to give **Compound 1*** (760 mg, 1.68 mmol) in 50% yield.

HRMS ESI$^+$, m/z calcd. for $C_{21}H_{21}BrN_6O$ $[M+H]^+$ 453.1033; found 453.1038.

^{1}H NMR (500 MHz, DMSO-*d6*) δ 14.11 (s, 1H), 7.87-7.93 (m, 2H), 7.52 (s, 1H), 7.22 (dd, *J* = 7.8, 7.8 Hz, 1H), 7.01 (s, 1H), 6.89-6.82 (m, 3H), 5.98 (s, 2H), 4.60 – 4.55 (m, 2H), 3.27 (d, *J* = 11.7 Hz, 2H), 3.00 (d, *J* = 11.7 Hz, 2H), 2.22-2.15 (m, 2H), 2.00-1.93 (m, 2H).

^{13}C NMR (126 MHz, DMSO-*d6*) δ 158.51, 154.53, 153.70, 152.41, 149.99, 143.08, 140.45, 130.27, 126.43, 118.50, 117.72, 117.40, 111.60, 111.42, 109.52, 53.70, 50.92, 27.03.

2.2. Synthesis of Compound 1

1-(prop-2-yn-1-yl)azepane (SI.1)

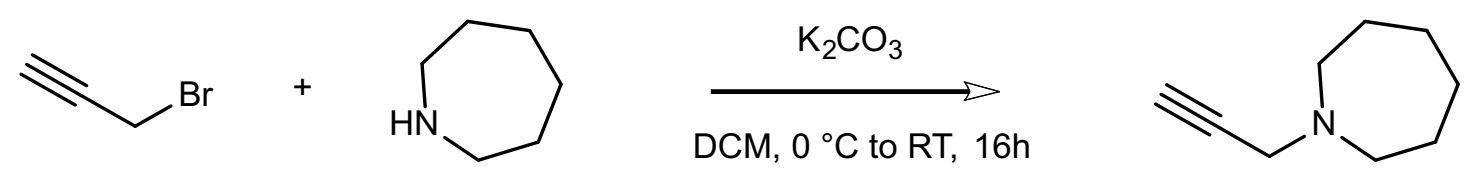

SI.1

To a suspension of potassium carbonate (1.9 g, 13.4 mmol, 2.0 eq.) and azepane (3.8 mL, 33.6 mmol, 5.0 eq.) in DCM was added 3-bromoprop-1-yne (1.0 g, 6.72 mmol, 1.0 eq.) at 0 °C. The reaction was stirred at room temperature for 16 hrs. Then the mixture was filtered, and the filtrate was concentrated. The residue was suspended in heptane and then filtered. The filtrate was concentrated to give the product as a mixture of bis-propargyl azepane and azepane, which was used in the next reaction without further purification.

2-(6-amino-5-(8-(2-(3-(azepan-1-yl)prop-1-yn-1-yl)pyridin-4-yl)-3,8-diazabicyclo[3.2.1]octan-3-yl)pyridazin-3-yl)phenol (1)

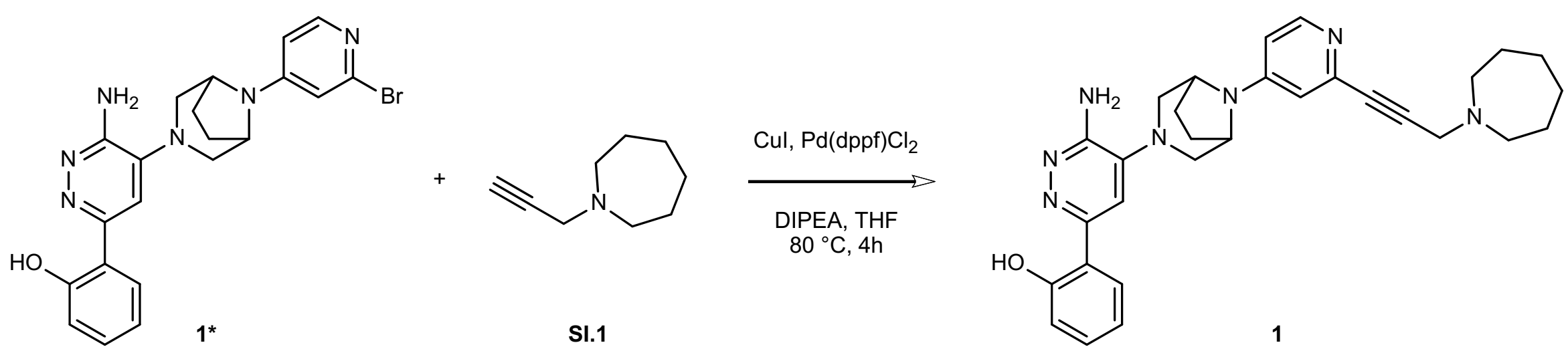

1* SI.1 1

To a solution of 1-prop-2-ynylazepane (45 mg, crude mixture from the previous reaction), Compound 1* (15 mg, 0.033 mmol, 1.0 eq), copper(I) iodide (0.63 mg, 3.3 μmol, 0.1 eq.) and *N*,*N*-diisopropylethylamine (0.06 mL, 0.33 mmol, 10.0 eq.) in THF (2.0 mL) was added [1,1'-[1,1'-bis(diphenylphosphino)ferrocene]dichloropalladium(II) (4.9 mg, 6.6 μmol, 0.2 eq.). After being stirred at 80 °C for 4 hrs under nitrogen atmosphere, the reaction was diluted with EtOAc and water. The mixture was extracted with EtOAc, washed with saturated aqueous NaCl, dried over Na_2SO_4, filtered, and concentrated. Column chromatography (ODS, MeCN/H_2O with 0.1% HCOOH) of the residue gave the product, which was purified with preparative HPLC (MeCN/H_2O with 0.1% NH_4OH) to give **Compound 1** (4.8 mg, 9.3 μmol) in 28% yield.

HRMS ESI$^+$, m/z calcd for $C_{30}H_{36}N_7O$ $[M+H]^+$ 510.2976; found 510.2989.

^{1}H NMR (500 MHz, CD_3OD) δ 8.06 (d, *J* = 6.1 Hz, 1H), 7.73 (m, 1H), 7.47 (s, 1H), 7.22 (m, 1H), 6.99 (m, 1H), 6.92 – 6.84 (m, 2H), 6.80 (m, 1H), 4.71 (s, 1H), 4.57 (s, 2H), 3.60 (s, 2H), 3.40 –

3.32 (m, 2H), 3.12-3.05 (m, 2H), 2.84-2.79 (m, 4H), 2.29-2.21 (m, 2H), 2.17-2.11 (m, 2H), 1.78-1.69 (m, 4H), 1.69-1.61 (m, 4H).

^{13}C NMR (126 MHz, CD_3OD) δ 159.74, 155.98, 155.93, 153.13, 150.66, 144.17, 142.64, 131.66, 127.26, 120.17, 119.18, 118.66, 113.83, 113.27, 110.07, 85.81, 85.27, 56.52, 55.51, 52.64, 49.10, 28.49, 28.41, 27.69.

2.3. Synthesis of Compound 2

***tert*-butyl 4-(4-(4-(3-(3-amino-6-(2-hydroxyphenyl)pyridazin-4-yl)-3,8-diazabicyclo[3.2.1]octan-8-yl)pyridin-2-yl)but-3-yn-1-yl)piperazine-1-carboxylate (SI.2)**

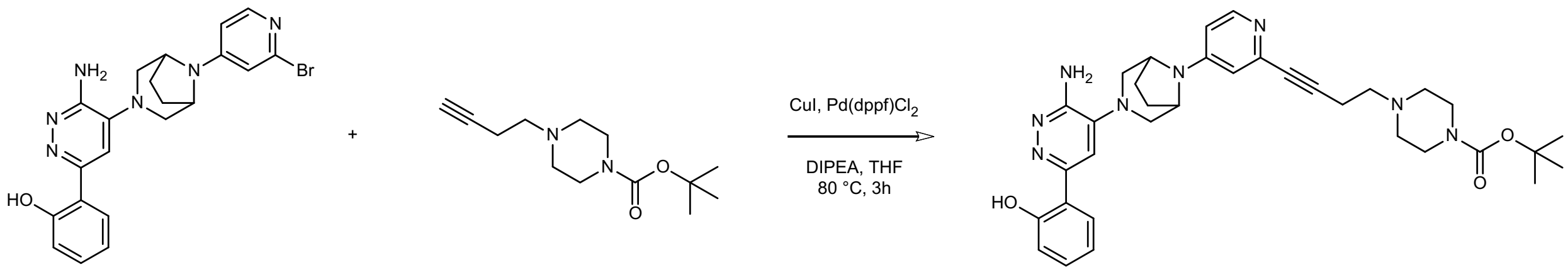

To a solution of Compound 1* (50 mg, 0.11 mmol, 1.0 eq.), tert-butyl 4-but-3-ynylpiperazine-1-carboxylate (237 mg, 0.99 mmol, 9.0 eq.), copper(I) iodide (6.3 mg, 0.033 mmol, 0.3 eq.), and *N*,*N*-diisopropylethylamine (0.38 mL, 2.2 mmol, 20 eq.) in THF (2.0 mL) was added [1,1'-bis(diphenylphosphino)ferrocene]dichloropalladium(II) (16 mg, 0.022 mmol, 0.20 eq.). After being stirred at 80 °C for 3 hrs under nitrogen atmosphere, the reaction was diluted with EtOAc and water. The mixture was extracted with EtOAc, washed with saturated aqueous NaCl, dried over Na_2SO_4, filtered, and concentrated. Column chromatography (ODS 13 g, $MeCN/H_2O$ with 0.1% HCOOH) of the residue gave the impure **SI.2** (70 mg), which was used in the next reaction without further purification.

2-[6-amino-5-[8-[2-(4-piperazin-1-ylbut-1-ynyl)-4-pyridyl]-3,8-diazabicyclo[3.2.1]octan-3-yl]pyridazin-3-yl]phenol (2)

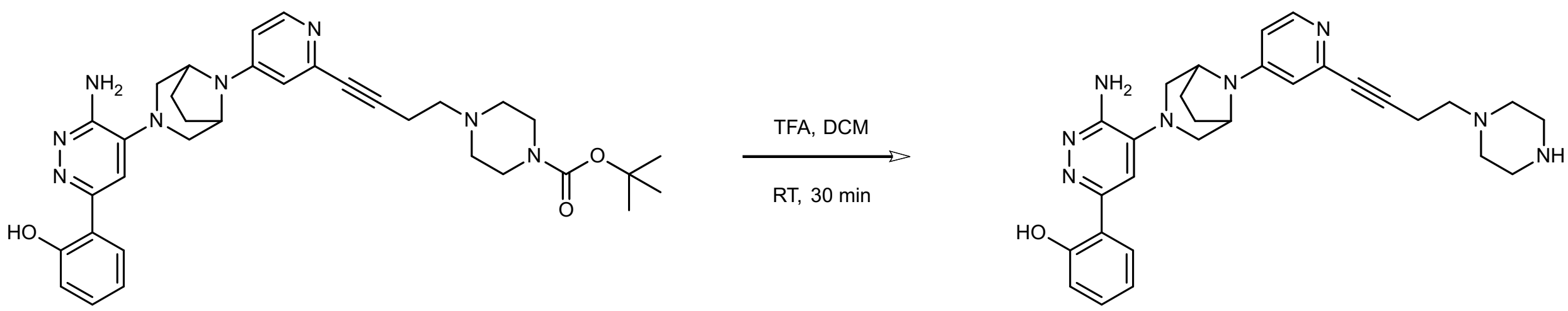

To a solution of **SI.2** (a mixture from the previous reaction, calculated as 0.11 mmol, 1.0 eq.) in DCM (0.5 mL) was added 2,2,2-trifluoroacetic acid (0.5 mL). The reaction mixture was stirred for 30 min and then evaporated. The residue was filtered and purified with preparative HPLC ($MeCN/H_2O$ with 0.1% NH_4OH) to give **Compound 2** (20 mg, 0.0398 mmol) in 36% yield over two steps.

HRMS ESI$^+$, m/z calcd for $C_{29}H_{34}N_8O$ $[M+H]^+$ 511.2983; found 511.2941.

^{1}H NMR (500 MHz, CD_3OD) δ 8.02 (d, *J* = 6.1 Hz, 1H), 7.71 (dd, *J* = 8.1, 1.8 Hz, 1H), 7.44 (s, 1H), 7.21 (ddd, *J* = 8.5, 7.3, 1.6 Hz, 1H), 6.94 (d, *J* = 2.5 Hz, 1H), 6.91 – 6.84 (m, 2H), 6.75 (dd, *J* = 6.2, 2.6 Hz, 1H), 4.54 (brd, *J* = 3.8 Hz, 2H), 3.36-3.32 (m, 2H), 3.06 (d, *J* = 11.6 Hz, 2H), 2.85 (m, 4H), 2.71 – 2.60 (m, 4H), 2.52 (m, 4H), 2.27-2.22 (m, 2H), 2.15-2.08 (m, 2H).

^{13}C NMR (126 MHz, CD_3OD) δ 159.76, 155.95, 155.90, 153.11, 150.51, 144.66, 142.60, 131.66, 127.27, 120.15, 119.20, 118.68, 113.68, 113.22, 109.87, 88.81, 82.07, 58.42, 55.48, 54.54, 52.54, 46.07, 28.41, 17.68.

2.4. Synthesis of Compound 3

1-(but-3-yn-1-yl)azepane (SI.3)

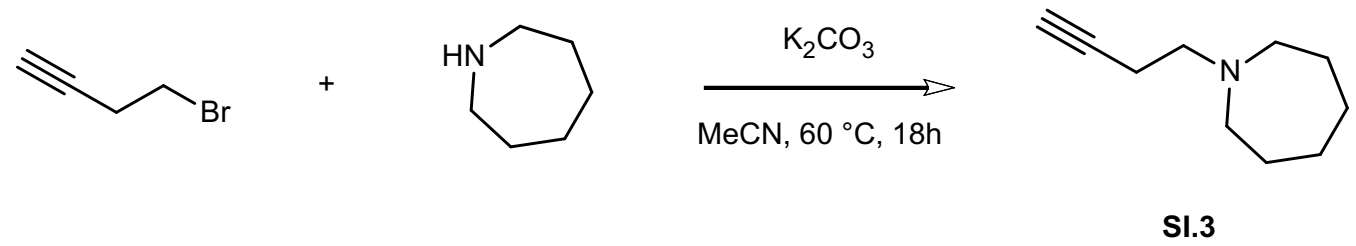

To a suspension of azepane (0.93 mL, 8.3 mmol, 1.1 eq.) and potassium carbonate (1.6 g, 11 mmol, 1.5 eq.) in MeCN (10 mL) was added 4-bromobut-1-yne (0.71 mL, 7.5 mmol, 1.0 eq.). After being stirred at 60 °C for 18 hrs, the reaction was diluted with DCM and water. The mixture was extracted with DCM, dried over Na_2SO_4, filtered, and concentrated to give impure 1-(but-3-yn-1-yl)azepane (410 mg), which was used in the next reaction without further purification.

2-(6-amino-5-(8-(2-(4-(azepan-1-yl)but-1-yn-1-yl)pyridin-4-yl)-3,8-diazabicyclo[3.2.1]octan-3-yl)pyridazin-3-yl)phenol (3)

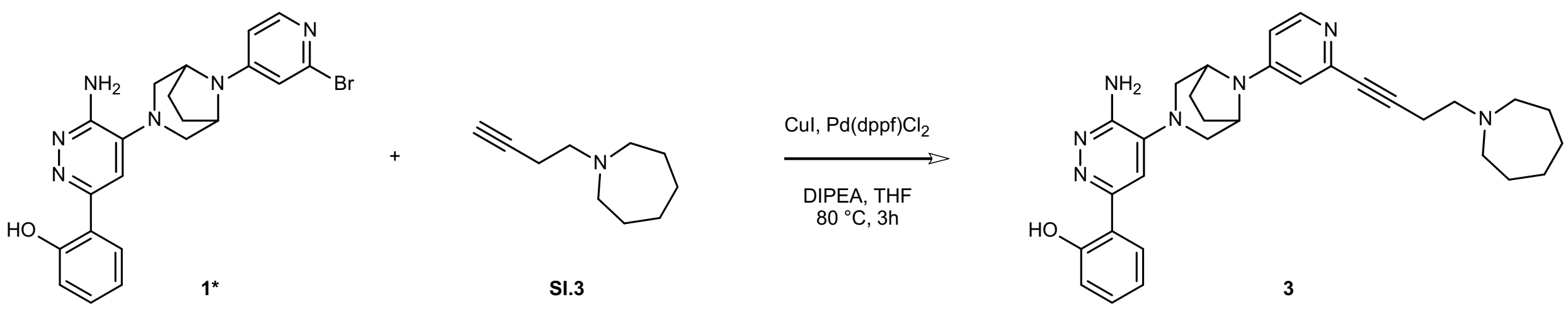

To a solution of Compound 1* (50 mg, 0.11 mmol, 1.0 eq.), a crude 1-(but-3-yn-1-yl)azepane (152 mg, calculated as 1.01 mmol, 9.1 eq.), copper(I) iodide (6.3 mg, 0.033 mmol, 0.3 eq.), and *N*,*N*-diisopropylethylamine (0.38 mL, 2.21 mmol, 20 eq.) in THF (2.0 mL) was added [1,1'-bis(diphenylphosphino)ferrocene]dichloropalladium(II) (16 mg, 0.022 mmol, 0.2 eq.). After being stirred at 80 °C for 3 hrs under nitrogen atmosphere, the reaction was diluted with EtOAc and water. The mixture was extracted with EtOAc, washed with saturated aqueous NaCl, dried over Na_2SO_4, filtered, and concentrated. Column chromatography (ODS, MeCN/H_2O with 0.1% HCOOH) of the residue gave the product, which was repurified with HPLC (MeCN/H_2O with 0.1% NH_4OH) to give **Compound 3** (12.1 mg, 0.023 mmol) in 21% yield.

HRMS ESI$^+$, m/z calcd for $C_{31}H_{37}N_7O$ $[M+H]^+$ 524.3132; found 524.3141.

^{1}H NMR (500 MHz, CD_3OD) δ 8.03 (d, *J* = 6.1 Hz, 1H), 7.72 (dd, *J* = 8.0, 1.6 Hz, 1H), 7.45 (s, 1H), 7.22 (ddd, *J* = 8.5, 7.2, 1.6 Hz, 1H), 6.94 (d, *J* = 2.4 Hz, 1H), 6.91-6.84 (m, 2H), 6.76 (dd, *J* = 6.1, 2.5 Hz, 1H), 4.54 (m, 2H), 3.33 (d, *J* = 11.6 Hz, 2H), 3.07 (d, *J* = 11.6 Hz, 2H), 2.83 (dd, *J*

= 8.3, 6.8 Hz, 2H), 2.77 – 2.71 (m, 4H), 2.62 (dd, *J* = 8.4, 6.9 Hz, 2H), 2.23 (m, 2H), 2.15 (m, 2H), 1.71-1.65 (m, 4H), 1.65-1.58 (m, 4H).

^{13}C NMR (126 MHz, CD_3OD) δ 159.77, 155.97, 155.93, 153.12, 150.50, 144.74, 142.63, 131.65, 127.26, 120.15, 119.20, 118.67, 113.66, 113.23, 109.84, 89.07, 82.06, 57.75, 56.21, 55.48, 52.55, 28.41, 28.33, 28.00, 18.03.

2.5. Synthesis of Compound 4

2-(6-amino-5-(8-(2-(3-(piperazin-1-yl)prop-1-yn-1-yl)pyridin-4-yl)-3,8-diazabicyclo[3.2.1]octan-3-yl)pyridazin-3-yl)phenol (4)

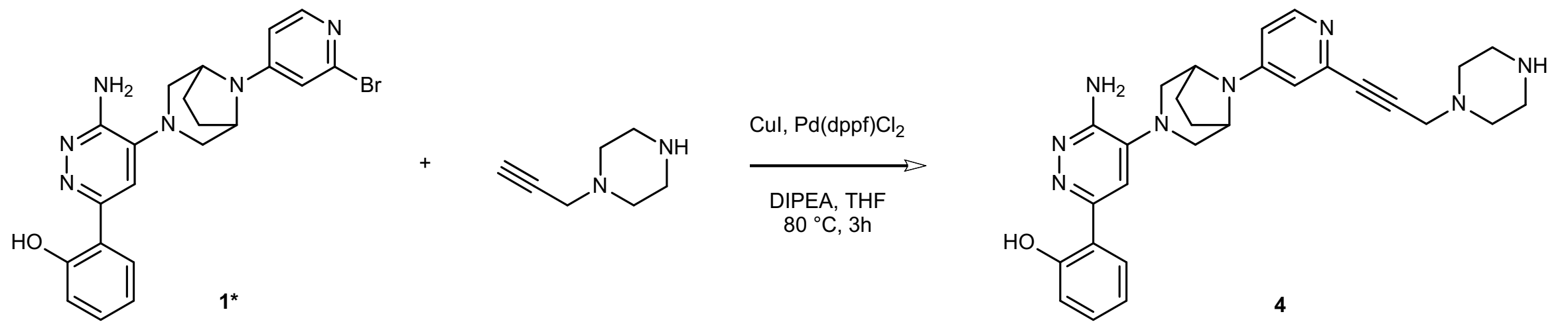

To a solution of Compound 1*, 1-prop-2-ynylpiperazine (75 mg, 0.605 mmol, 9.1 eq.), copper(I) iodide (3.8 mg, 0.020 mmol, 0.3 eq.), and *N*,*N*-diisopropylethylamine (0.23 mL, 1.32 mmol, 20 eq.) in THF (2.0 mL) was added [1,1'-bis(diphenylphosphino)ferrocene]dichloropalladium(II) (9.7 mg, 0.013 mmol, 0.2 eq.). After being stirred at 80 °C for 3 hrs under nitrogen atmosphere, the reaction was diluted with EtOAc and was added water. The mixture was extracted with EtOAc, washed with saturated aqueous NaCl, dried over Na_2SO_4, filtered, and concentrated. Column chromatography (ODS, MeCN/H_2O with 0.1% HCOOH) of the residue gave the product, which was re-purified with preparative HPLC (MeCN/H_2O with 0.1% NH_4OH) to give **Compound 4** (12.4 mg, 0.025 mmol) in 38% yield.

HRMS ESI$^+$, m/z calcd for $C_{28}H_{32}N_8O$ $[M+H]^+$ 497.2772; found 497.2783.

^{1}H NMR (500 MHz, CD_3OD) δ 8.06 (d, *J* = 6.0 Hz, 1H), 7.73 (dd, *J* = 8.0, 1.6 Hz, 1H), 7.47 (s, 1H), 7.22 (ddd, *J* = 8.5, 7.2, 1.6 Hz, 1H), 7.01 (d, *J* = 2.5 Hz, 1H), 6.91-6.85 (m, 2H), 6.80 (dd, *J* = 6.1, 2.6 Hz, 1H), 4.58 (dd, *J* = 4.7, 2.5 Hz, 2H), 3.54 (s, 2H), 3.39 – 3.32 (m, 2H), 3.09 (d, *J* = 11.5 Hz, 2H), 2.89 (m, 4H), 2.66 (m, 4H), 2.29-2.22 (m, 2H), 2.17-2.09 (m, 2H).

^{13}C NMR (126 MHz, CD_3OD) δ 159.75, 155.99, 155.93, 153.13, 150.68, 144.03, 142.63, 131.66, 127.26, 120.16, 119.19, 118.67, 113.88, 113.27, 110.12, 86.20, 84.58, 55.50, 53.51, 52.63, 48.43, 46.07, 28.41.

2.6. Synthesis of Compound 5

2-(6-amino-5-(8-(2-(3-cycloheptylprop-1-yn-1-yl)pyridin-4-yl)-3,8-diazabicyclo[3.2.1]octan-3-yl)pyridazin-3-yl)phenol (5)

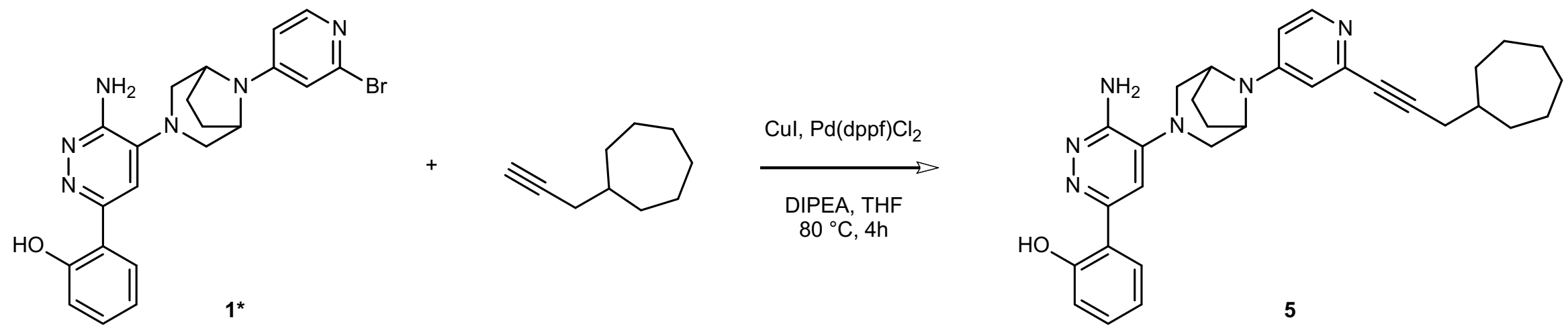

To a solution of Compound 1* (20 mg, 0.044 mmol, 1.0 eq.), prop-2-ynylcycloheptane (50 mg, 0.37 mmol, 8.3 eq.), copper(I) iodide (2.5 mg, 0.0132 mmol, 0.3 eq.), and *N*,*N*-diisopropylethylamine (0.077 mL, 0.44 mmol, 10 eq.) in THF (2.0 mL) was added [1,1'-bis(diphenylphosphino)ferrocene]dichloropalladium(II) (6.5 mg, 0.0088 mmol, 0.2 eq). After being stirred at 80 °C for 4 hrs under nitrogen atmosphere, the reaction was diluted with EtOAc and water. The mixture was extracted with EtOAc, washed with saturated aqueous NaCl, dried over Na_2SO_4, filtered, and concentrated. Column chromatography (ODS, MeCN/H_2O with 0.1% HCOOH) of the residue gave the product, which was re-purified with preparative HPLC (MeCN/H_2O with 0.1% NH_4OH) to give **Compound 5** (10.0 mg, 0.020 mmol) in 45% yield.

HRMS ESI$^+$, m/z calcd for $C_{31}H_{36}N_6O$ $[M+H]^+$ 509.3023; found 509.3030.

^{1}H NMR (500 MHz, CD_3OD) δ 8.02 (d, *J* = 6.1 Hz, 1H), 7.76 – 7.71 (m, 1H), 7.47 (s, 1H), 7.22 (ddd, *J* = 7.7, 7.7, 1.6 Hz, 1H), 6.94 – 6.85 (m, 3H), 6.76 (m, 1H), 4.56 (t, J = 3.8 Hz, 2H), 3.34 (d, *J* = 11.5 Hz, 2H), 3.08 (d, *J* = 11.5 Hz, 2H), 2.34 (d, *J* = 6.8 Hz, 2H), 2.27-2.20 (m, 2H), 2.17-2.10 (m, 2H), 1.94-1.85 (m, 2H), 1.77 (m, 1H), 1.74-1.66 (m, 2H), 1.66 – 1.57 (m, 2H), 1.57-1.42 (m, 4H), 1.41-1.31 (m, 2H).

^{13}C NMR (126 MHz, CD_3OD) δ 159.75, 155.98, 155.95, 153.13, 150.44, 145.02, 142.67, 131.65, 127.27, 120.17, 119.19, 118.66, 113.51, 113.25, 109.67, 90.57, 81.88, 55.47, 52.60, 40.54, 35.50, 29.40, 28.56, 28.42, 27.42.

Supplementary Figure legends

Supplementary Figure 1| cryo-EM data processing Part 1.
Workflow for Cryo-EM data processing for 0º and 30º datasets.

Supplementary Figure 2| cryo-EM data processing Part 2.
a, Workflow for Cryo-EM data processing of combined 0º and 30º datasets. **b**, **c**, Angular distribution plot (**b**) and posterior position directional distribution plot (**c**) for the final local refinement. **d**, Gold-standard Fourier shell correlation at a cut-off of 0.143. **e**, Local resolution estimation.

Supplementary Figure 3| Gating strategy for flow cytometry and FACS.
Gating strategy for SMARCA2 stability CRISPR/Cas9 screens, DCAF16 deep mutational scanning screens, and CRISPR screen validation.

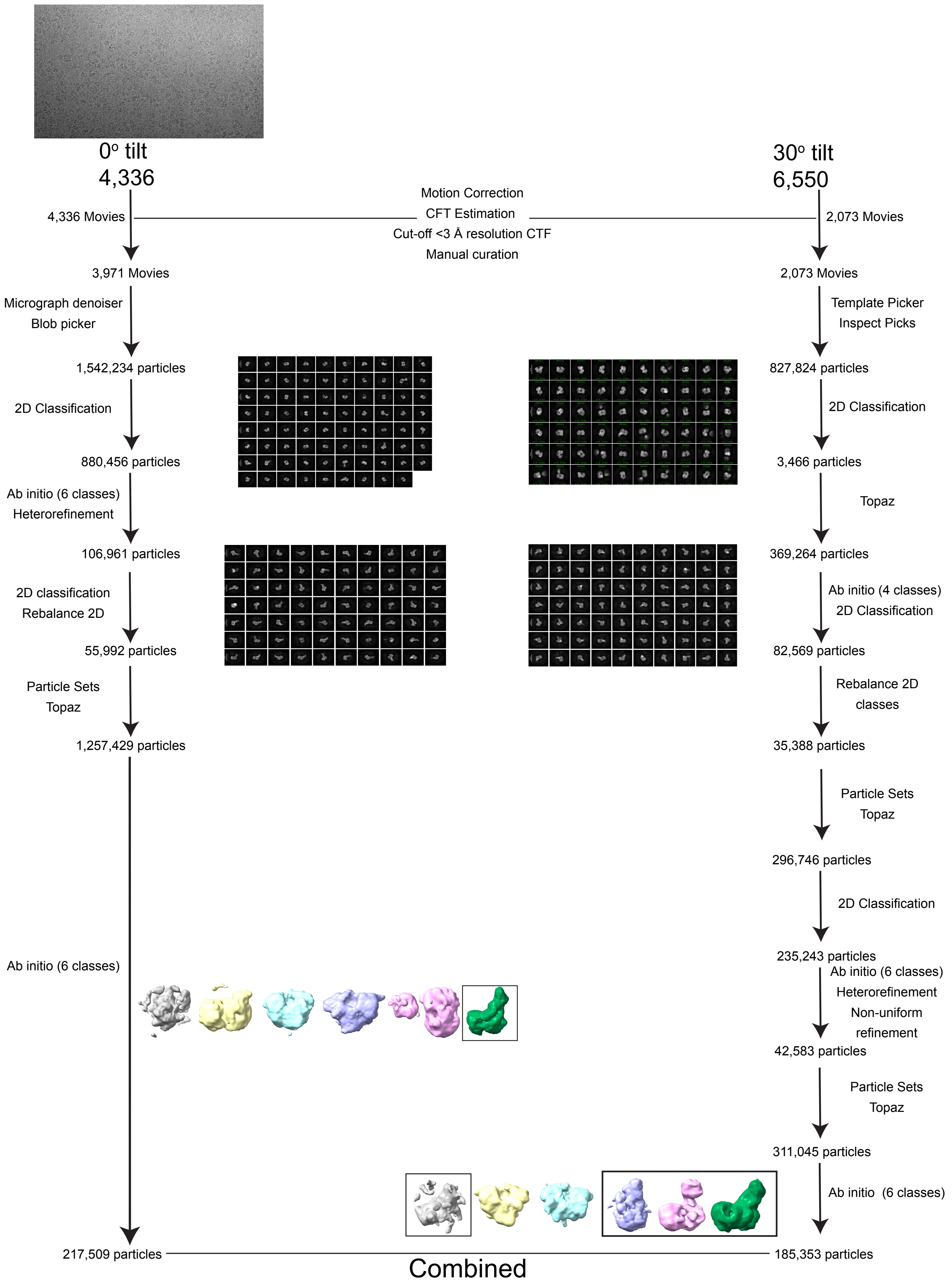

0° tilt
4,336
30° tilt
6,550
Motion Correction
CFT Estimation
Cut-off <3 Å resolution CTF
Manual curation
4,336 Movies
2,073 Movies
3,971 Movies
2,073 Movies
Micrograph denoiser
Blob picker
Template Picker
Inspect Picks
1,542,234 particles
827,824 particles
2D Classification
2D Classification
880,456 particles
3,466 particles
Ab initio (6 classes)
Heterorefinement
Topaz
106,961 particles
369,264 particles
2D classification
Rebalance 2D
Ab initio (4 classes)
2D Classification
55,992 particles
82,569 particles
Particle Sets
Topaz
Rebalance 2D
classes
1,257,429 particles
35,388 particles
Particle Sets
Topaz
296,746 particles
2D Classification
235,243 particles
Ab initio (6 classes)
Ab initio (6 classes)
Heterorefinement
Non-uniform
refinement
42,583 particles
Particle Sets
Topaz
311,045 particles
Ab initio (6 classes)
217,509 particles
185,353 particles
Combined

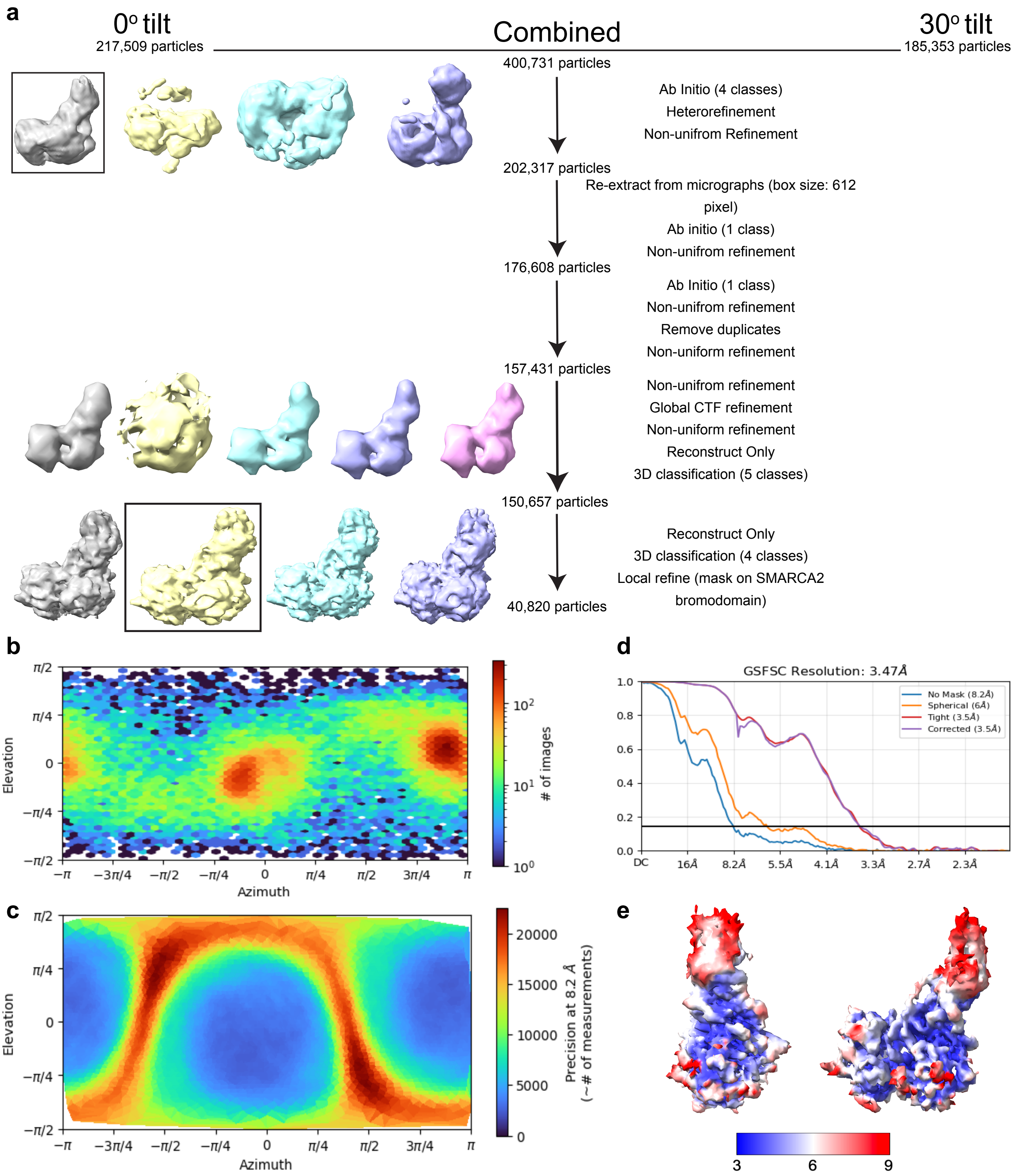
a
0° tilt
217,509 particles
Combined
30° tilt
185,353 particles
400,731 particles
Ab Initio (4 classes)
Heterorefinement
Non-unifrom Refinement
202,317 particles
Re-extract from micrographs (box size: 612 pixel)
Ab initio (1 class)
Non-unifrom refinement
176,608 particles
Ab Initio (1 class)
Non-unifrom refinement
Remove duplicates
Non-uniform refinement
157,431 particles
Non-unifrom refinement
Global CTF refinement
Non-unifrom refinement
Reconstruct Only
3D classification (5 classes)
150,657 particles
Reconstruct Only
3D classification (4 classes)
Local refine (mask on SMARCA2 bromodomain)
40,820 particles
b
Elevation
Azimuth
# of images
c
Elevation
Azimuth
Precision at 8.2 Å (~# of measurements)
d
GSFSC Resolution: 3.47Å
No Mask (8.2Å)
Spherical (6Å)
Tight (3.5Å)
Corrected (3.5Å)
DC
16Å
8.2Å
5.5Å
4.1Å
3.3Å
2.7Å
2.3Å
e
3
6
9

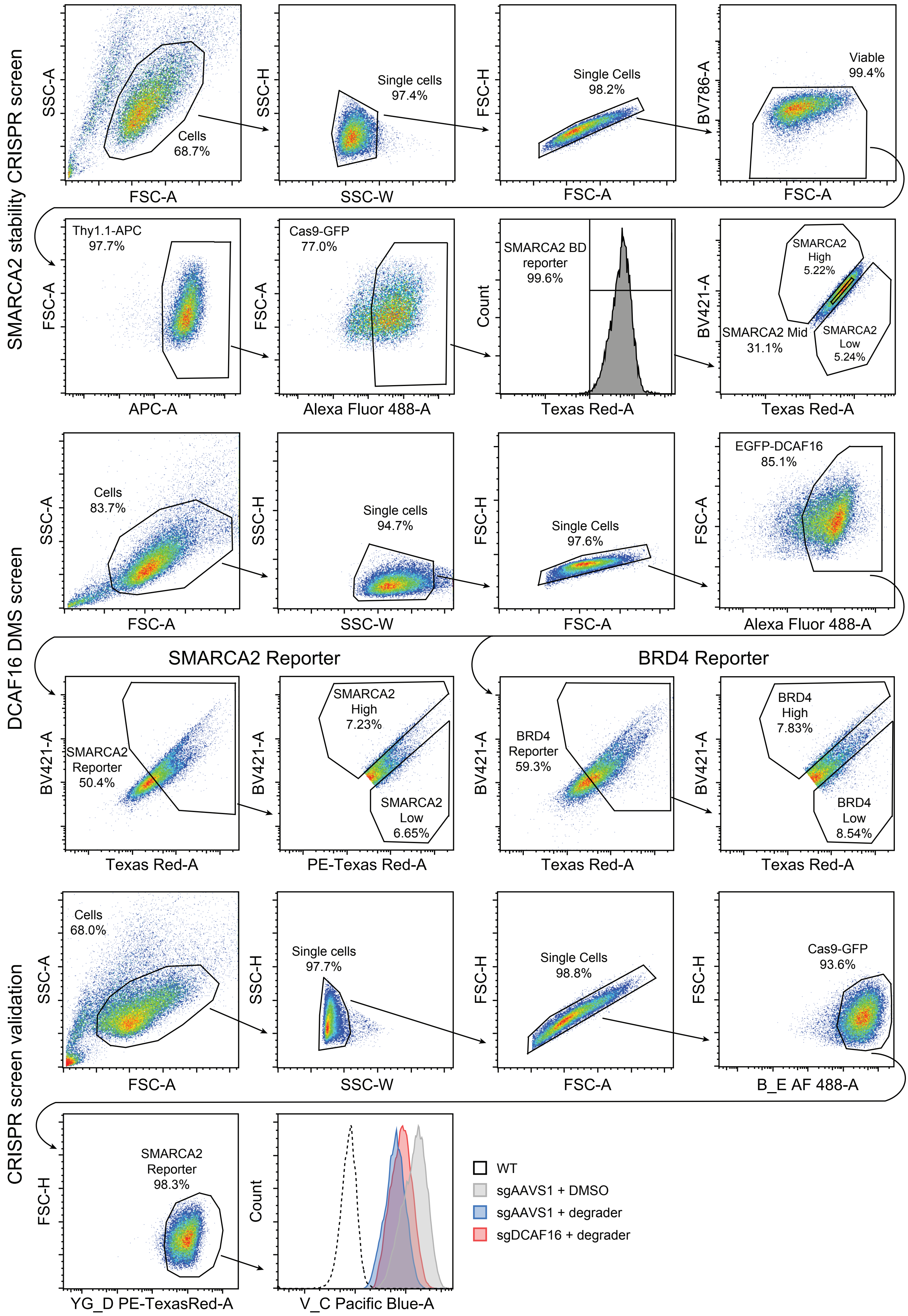

SMARCA2 stability CRISPR screen
SSC-A
FSC-A
Cells
68.7%
SSC-H
SSC-W
Single cells
97.4%
FSC-H
Single Cells
98.2%
BV786-A
Viable
99.4%
FSC-A
APC-A
Thy1.1-APC
97.7%
Alexa Fluor 488-A
Cas9-GFP
77.0%
Count
Texas Red-A
SMARCA2 BD
reporter
99.6%
BV421-A
SMARCA2
High
5.22%
SMARCA2 Mid
31.1%
SMARCA2
Low
5.24%
DCAF16 DMS screen
Cells
83.7%
Single cells
94.7%
Single Cells
97.6%
EGFP-DCAF16
85.1%
SMARCA2 Reporter
SMARCA2
Reporter
50.4%
PE-Texas Red-A
SMARCA2
High
7.23%
SMARCA2
Low
6.65%
BRD4 Reporter
BRD4
Reporter
59.3%
BRD4
High
7.83%
BRD4
Low
8.54%
CRISPR screen validation
Cells
68.0%
Single cells
97.7%
Single Cells
98.8%
Cas9-GFP
93.6%
B_E AF 488-A
SMARCA2
Reporter
98.3%
YG_D PE-TexasRed-A
V_C Pacific Blue-A
WT
sgAAVS1 + DMSO
sgAAVS1 + degrader
sgDCAF16 + degrader

Supplementary table 5: Plasmids used in this study.

Plasmid name	Application
pRRL-SFFV-SMARCA2(BD)-3xV5-mTagBFP-P2A-mCherry	Protein stability reporter, genetic screens
pRRL-SFFV-BRD4(TandemBDs)-3xV5-mTagBFP-P2A-mCherry	Protein stability reporter, genetic screens
pRRL-U6-sgRNA-EF1as-Thy1.1-P2A-NeoR	Knockouts in KBM7 iCas9 cells
px458-U6-sgRNA-CMV-3xFLAG-pSpCas9(BB)-T2A-GFP	Knockouts in 293T cells
pRRL-SFFV-EGFP-cpHalo-DCAF16(Lib)-EF1as-PuroR	DCAF16 Deep Mutational Scanning library
pRRL-SFFV-3xFLAG-DCAF16-EF1αs-iRFP670	Exogenous expression of DCAF16
pRRL-SFFV-3xFLAG-DCAF16-(Y62T)-EF1αs-iRFP670	Exogenous expression of DCAF16
pRRL-SFFV-3xFLAG-DCAF16(C173S)-EF1αs-iRFP670	Exogenous expression of DCAF16
pRRL-SFFV-3xFLAG-DCAF16-(S51K)-EF1αs-iRFP670	Exogenous expression of DCAF16
pRRL-SFFV-3xFLAG-DCAF16-(L59N)-EF1αs-iRFP670	Exogenous expression of DCAF16
pRRL-SFFV-3xFLAG-DCAF16-(L59W)-EF1αs-iRFP670	Exogenous expression of DCAF16
pRRL-SFFV-3xFLAG-DCAF16-(Y165D)-EF1αs-iRFP670	Exogenous expression of DCAF16
pRRL-SFFV-3xFLAG-DCAF16-(S172C)-EF1αs-iRFP670	Exogenous expression of DCAF16
pRRL-SFFV-3xFLAG-DCAF16-(G180N)-EF1αs-iRFP670	Exogenous expression of DCAF16
pRRL-SFFV-3xFLAG-DCAF16-(E188C)-EF1αs-iRFP670	Exogenous expression of DCAF16
pRRL-SFFV-3xFLAG-DCAF16-(I195W)-EF1αs-iRFP670	Exogenous expression of DCAF16
pLEX-hPGK-Puro-P2A-2xHA-FBXO22(WT)	Exogenous expression of FBXO22
pLEX-hPGK-Puro-P2A-2xHA-FBXO22(C143A)	Exogenous expression of FBXO22
pLEX-hPGK-Puro-P2A-2xHA-FBXO22(C227A)	Exogenous expression of FBXO22
pLEX-hPGK-Puro-P2A-2xHA-FBXO22(C228A)	Exogenous expression of FBXO22
pLEX-hPGK-Puro-P2A-2xHA-FBXO22(C326A)	Exogenous expression of FBXO22
pLEX-hPGK-Puro-P2A-2xHA-FBXO22(C365A)	Exogenous expression of FBXO22
pLEX-hPGK-Puro-P2A-2xHA-SPIN4	Exogenous expression of SPIN4
pcDNA5 FRT-TO miniTurbo-HiBiT-SMARCA2-BD	SMARCA2-BD BioID
pRK5-HA-DCAF16 WT	Exogenous expression of DCAF16
pCMV5 HA-FBXO22	Exogenous expression of FBXO22
LgBiT Expression Vector (N2681)	Exogenous expression of LargeBiT
HaloTag®-Ubiquitin Fusion Vector (N272A)	Exogenous expression of HaloTag-Ubiquitin
pFASTBacDUAL His-TEV-DCAF16 (C58S)	Recombinant Protein Production
pFASTBacDUAL His-TEV-DCAF16 (L59W)	Recombinant Protein Production
pFASTBacDUAL His-TEV-DCAF16 (C173S)	Recombinant Protein Production
GST-Precision Protease-SMARCA2 (1376-1590 d1403-1420)	Recombinant Protein Production
His-SUMO-SMARCA2 (1376-1590 d1403-1420)	Recombinant Protein Production
pFASTBacDUAL DDB1 (dBPB):DDA1	Recombinant Protein Production
pFASTBacDUAL His-TEV-DCAF16:DDB1:DDA1	Recombinant Protein Production
Flp-recombinase expression vector (pOG44)	Stable cell line generation

Supplementary Table 6: sgRNAs used in this study.

sgRNA/Oligo	Sequence (5' to 3')	Application
sgAAVS1	GCTGTGCCCCGATGCACAC	Knockout control
sgDCAF16	GGACTCCACAAGAGGCCAGA	*DCAF16* knockout
sgFBXO22	GATCCAGGTTACGCTCCGAT	*FBXO22* knockout
sgSMARCA2	CGGATGATGAGTGATCAGTA	SMARCA2 HiBiT knockin
sgSMARCA4	CCATCTCAGCTCTGGAACGA	SMARCA4 HiBiT knockin
SMARCA2 ssODN	CTTTTAGGAACAGTCAGAAGGAAGTGGGACGGATGATGAGAGTGGTTCTGGAGTGAGCGGCTGGCGGCTGTTCAAGAAGATTAGCTGATCAGTATGCACATTTTTCCTTGGTAGAACTGAATTCCTTCCTC	SMARCA2 HiBiT knockin
SMARCA4 ssODN	CCCTCTCCAGGACCGCTCAGGAAGTGGCAGCGAAGAAGACAGTGGTTCTGGAGTGAGCGGCTGGCGGCTGTTCAAGAAGATTAGCTGAGGGCCGACATTCCAGTCTCGACCCCGAGCCACTCGTTCCAGAGCTGA	SMARCA4 HiBiT knockin

Supplementary table 7: Summary of cryo-EM data collection conditions and image processing.

	EMDB-xxxx<br>PDB xxxx
**Data collection and processing**	
Magnification	165000
Voltage (kV)	300
Electron exposure (e–/Å^2)	60
Defocus range (μm)	-2.8 to -1.0
Pixel size (Å)	0.513
Symmetry imposed	C1
Initial particle images (no.)	2,370,058
Final particle images (no.)	40,820
Map resolution (Å)	3.47
FSC threshold	0.143
Map resolution range (Å) (min-max)	3.219-59.653
25th percentile, median and 75th percentile	5.264, 8.136, 10.595
Tilt applied	0° and 30°
**Refinement**	
Initial model used (PDB code)	8G46, AlphaFold
Model resolution (Å)	3.3
FSC threshold	0.143
Model resolution range (Å)	3.2-3.9
Map sharpening *B* factor (Å^2)	0
Model composition	
Non-hydrogen atoms	8,695
Protein residues	1,084
Ligands	T58, ZN
B factors (Å^2)	
Protein (mean)	186.79
Ligand (mean)	164.70
Model-Map	
CC (mask)	0.73
R.m.s. deviations	
Bond lengths (Å)	0.013
Bond angles (°)	1.204
Validation	
MolProbity score	2.40
Clashscore	54.92
CaBLAM outliers (%)	1.05
Rotamer outliers (%)	0.21
CB outliers (%)	0.00
Ramachandran plot	
Favored (%)	96.90
Allowed (%)	3.10
Disallowed (%)	0.00
EMringer	1.40
