# Supplementary material for: Dual E3 ligase recruitment by monovalent degraders for tunable SMARCA 2/4 degradation
Source: Nat Chem Biol. 2026 May 12;22(10):1666–75. doi: 10.1038/s41589-026-02224-y (PMC13612222; doi:10.1038/s41589-026-02224-y)

EDF3d

DCAF16 WT
+SMARCA2
+ **1**

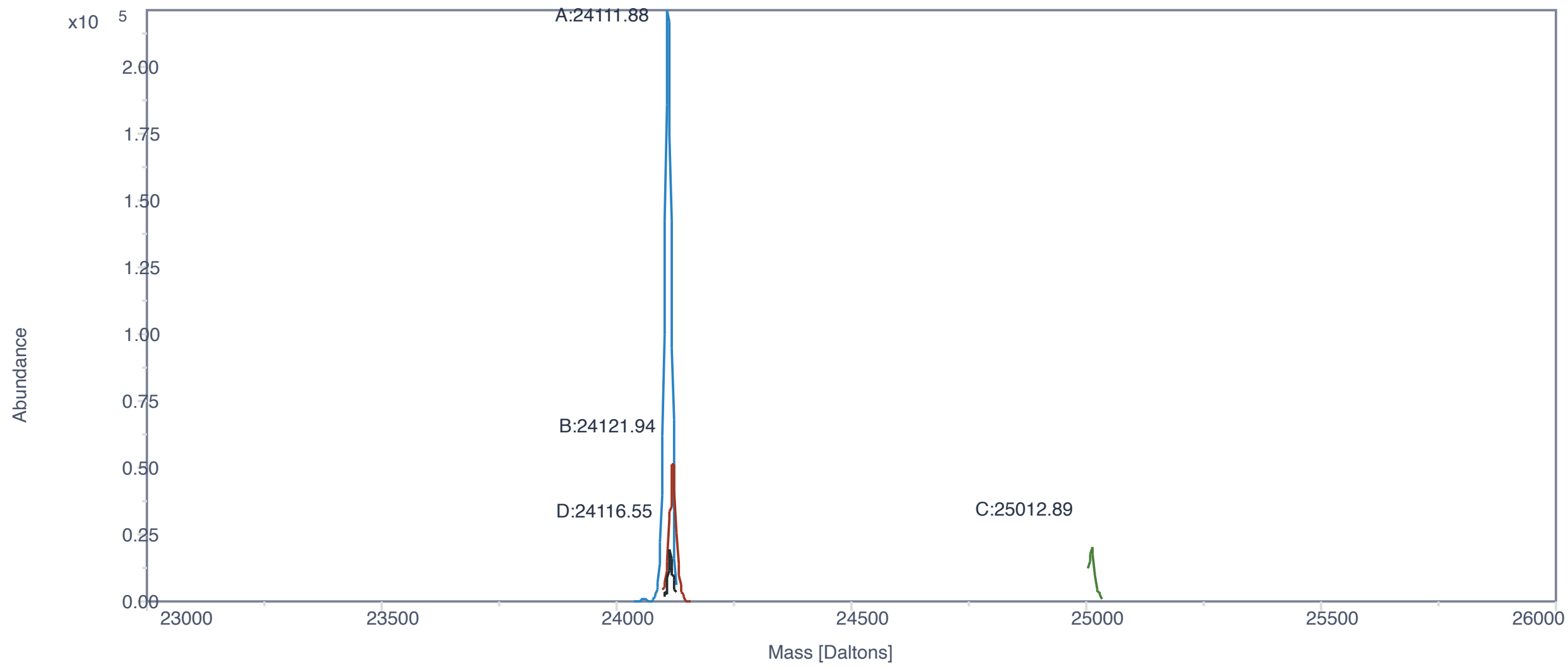

EDF3e

DCAF16 WT
+MMH2

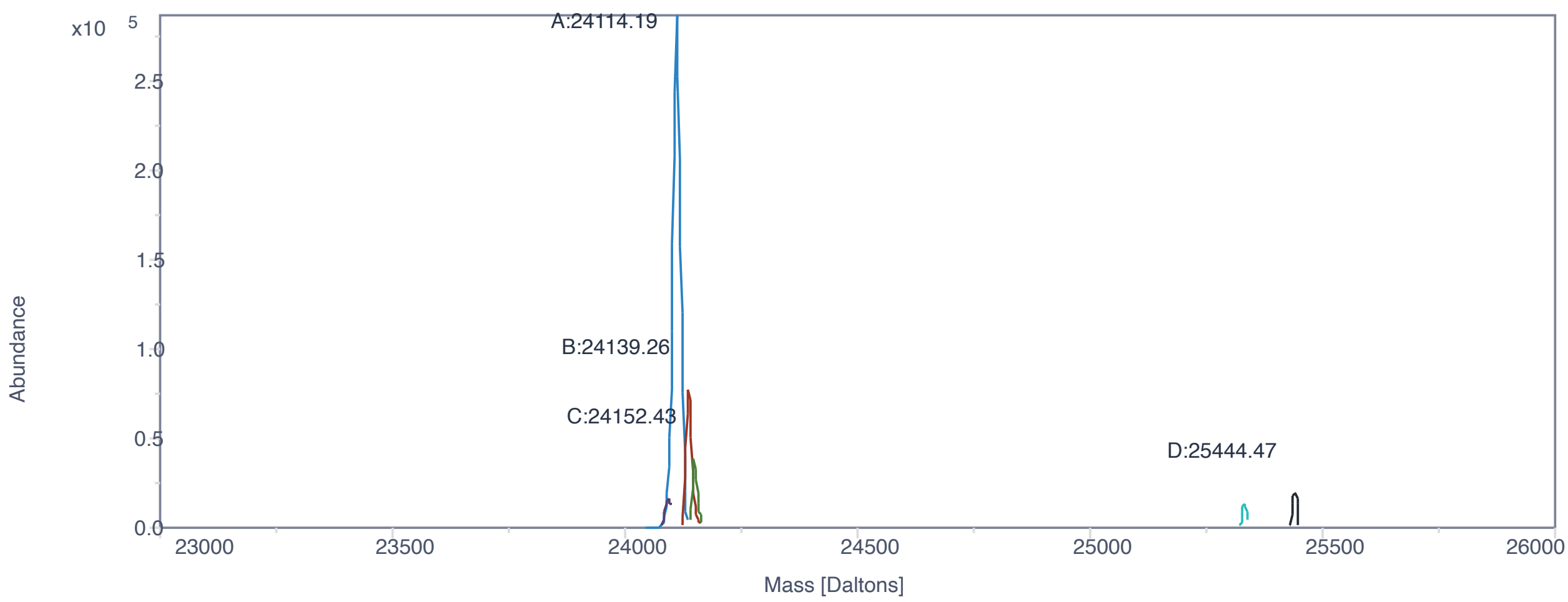

DCAF16 WT
+BRD4 BD2
+MMH2

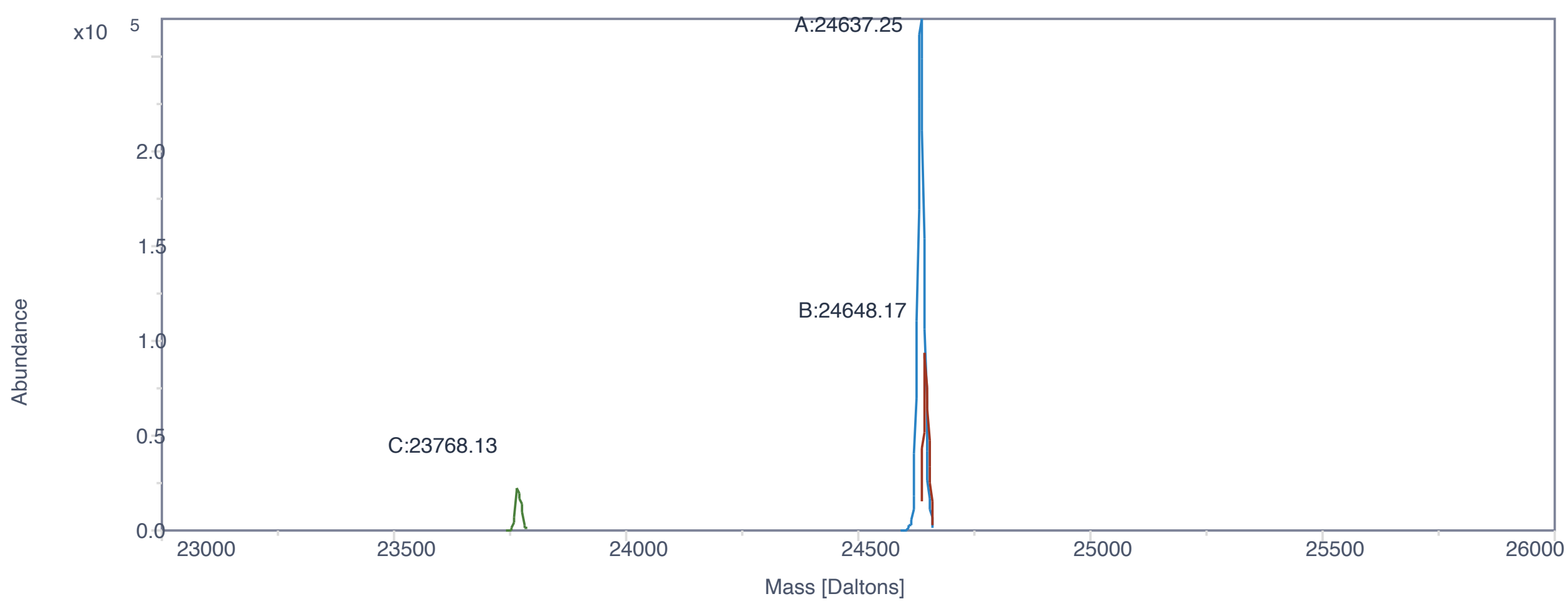

Supplement: Supplementary file 6 [file 41589_2026_2224_MOESM20_ESM.pdf]
