# Supplementary material for: Dual E3 ligase recruitment by monovalent degraders for tunable SMARCA 2/4 degradation
Source: Nat Chem Biol. 2026 May 12;22(10):1666–75. doi: 10.1038/s41589-026-02224-y (PMC13612222; doi:10.1038/s41589-026-02224-y)

EDF 5a

SMARCA2

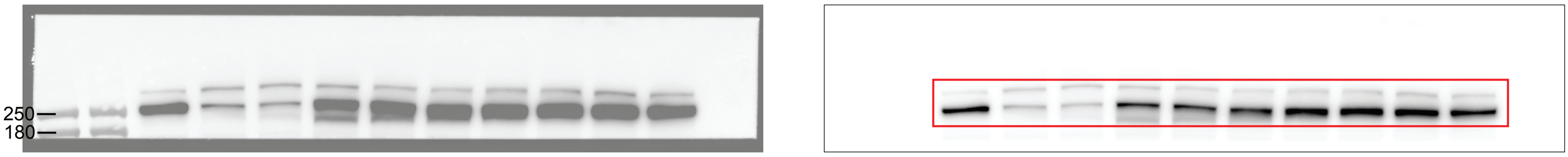

HSP90

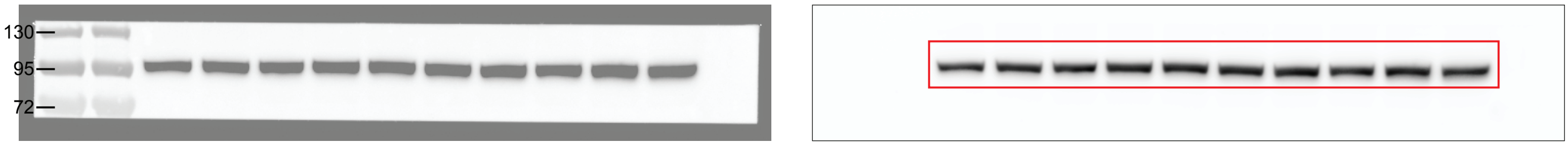

EDF 5b

IP HA: Streptavidin

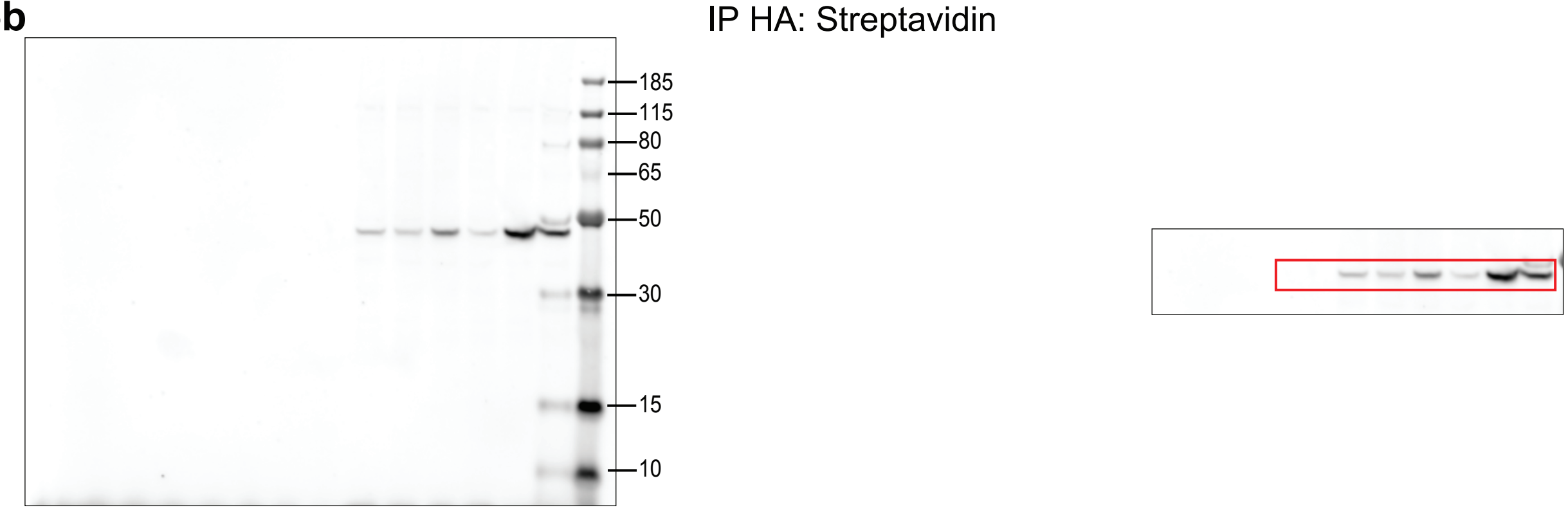

IP HA: HA

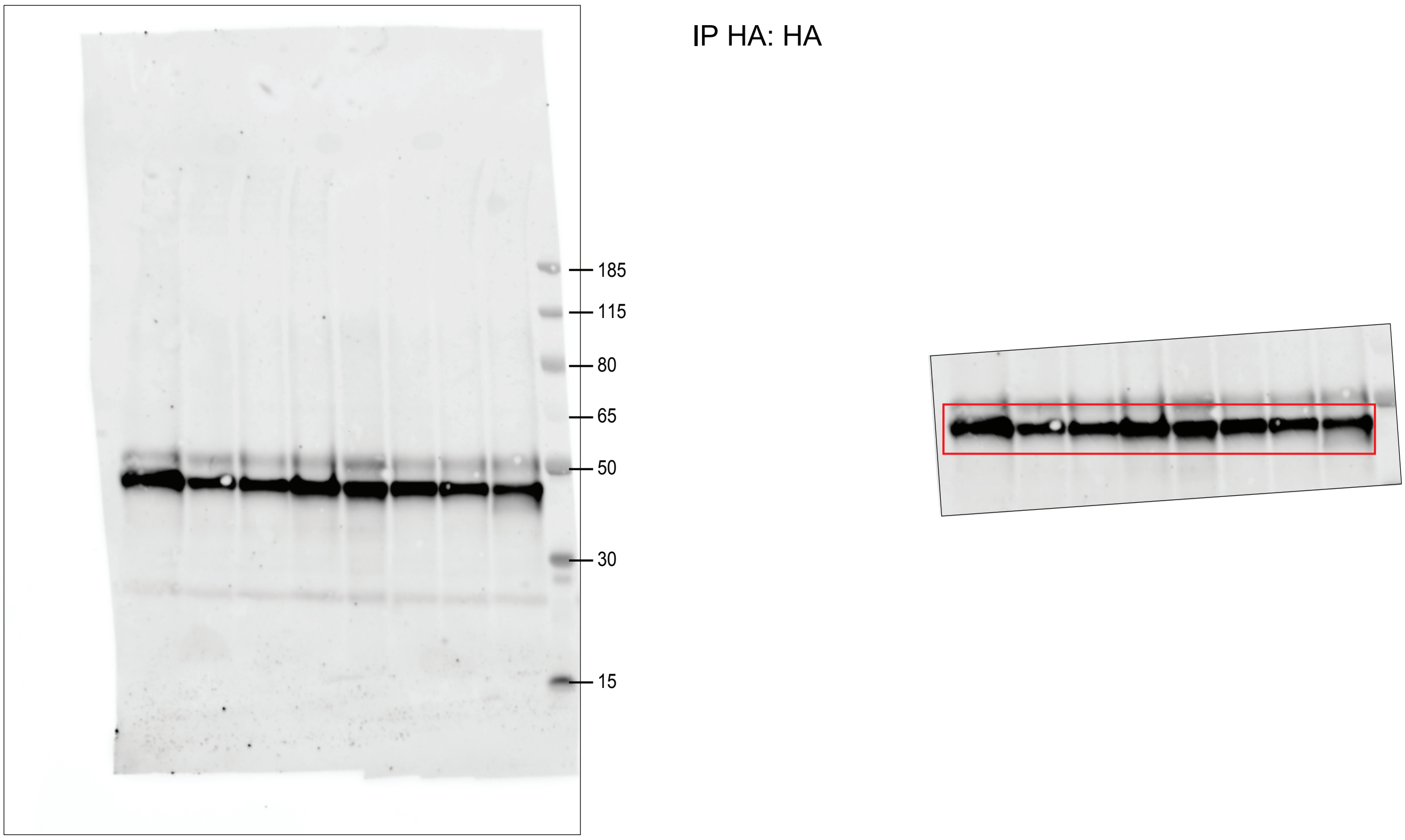

EDF 5b

Input: Streptavidin

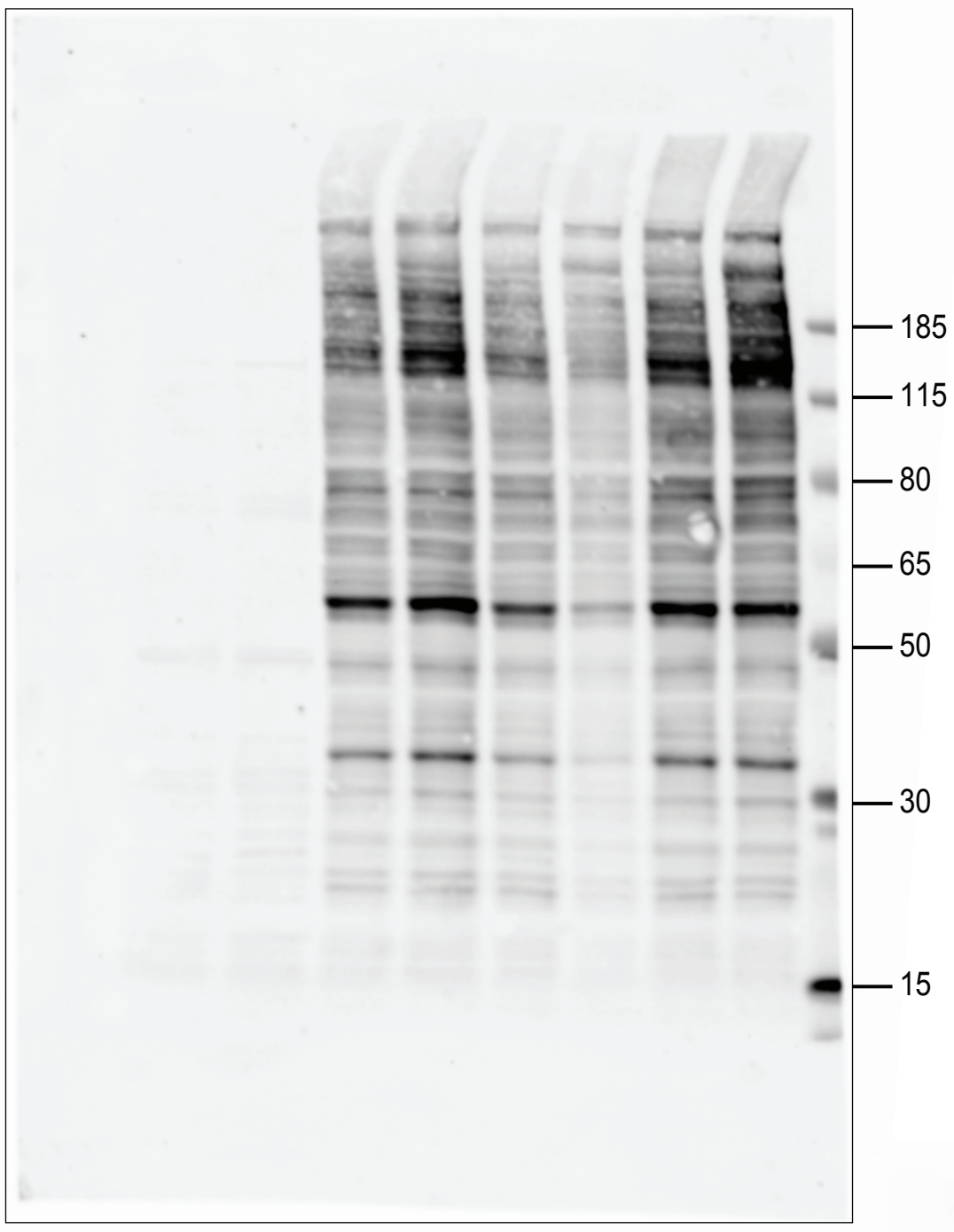

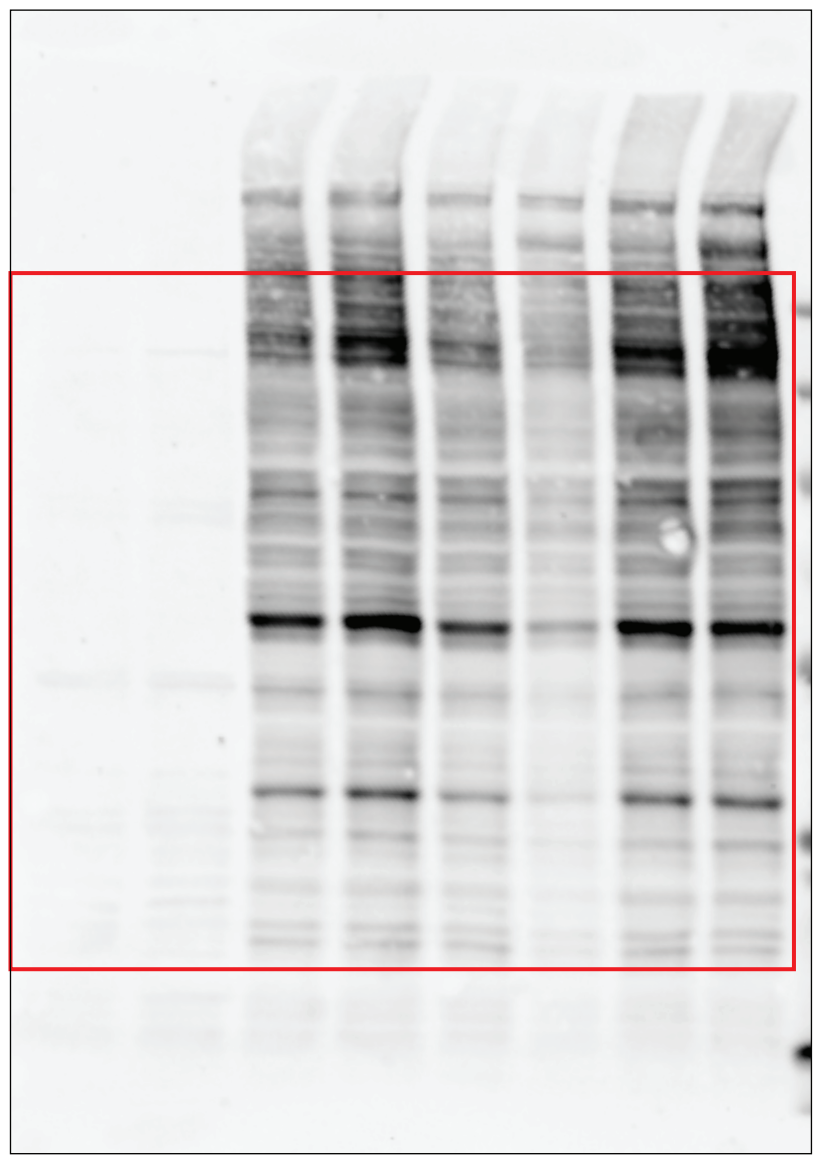

Input: HiBiT

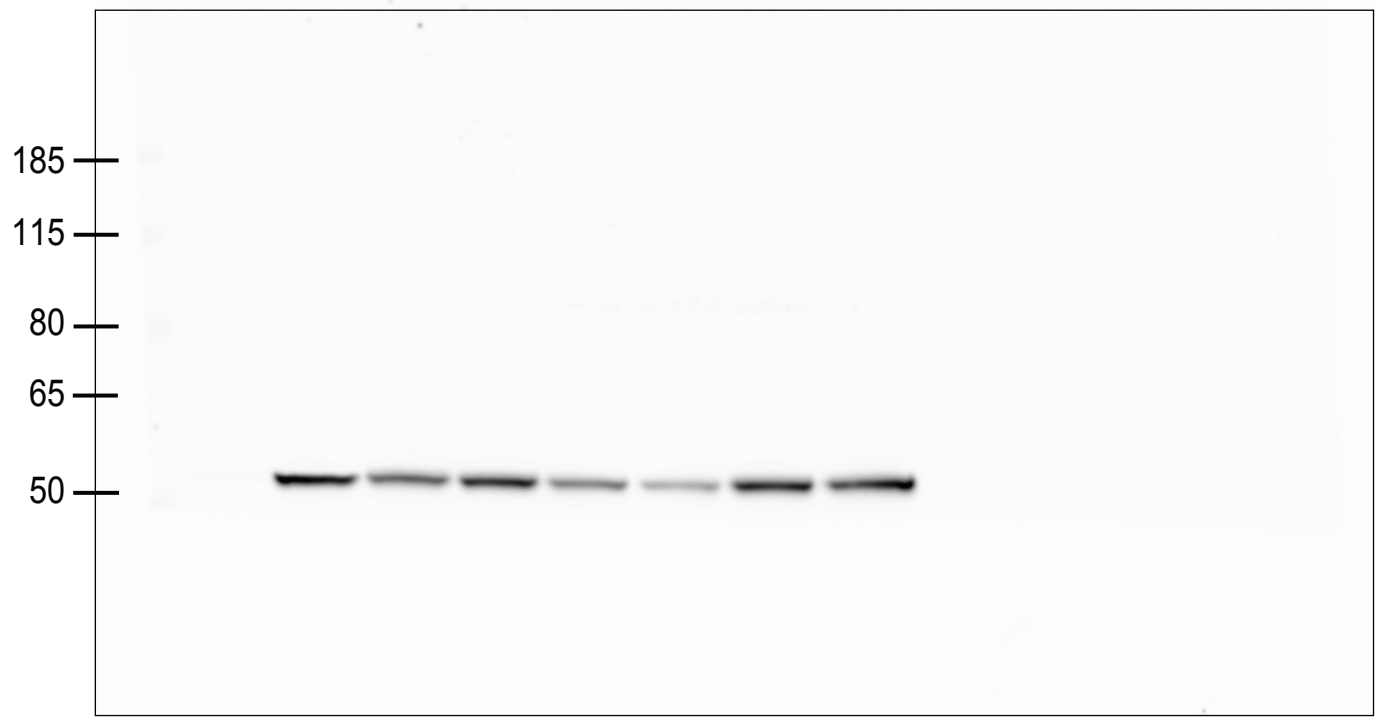

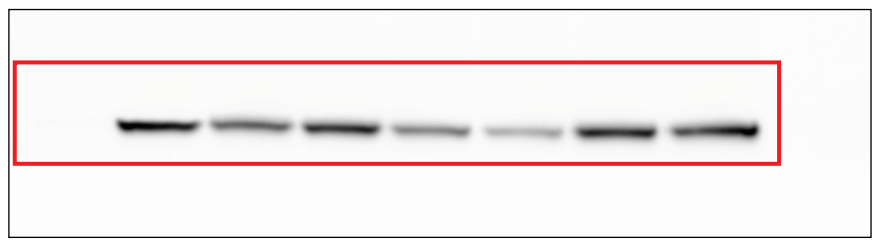

Input: HA

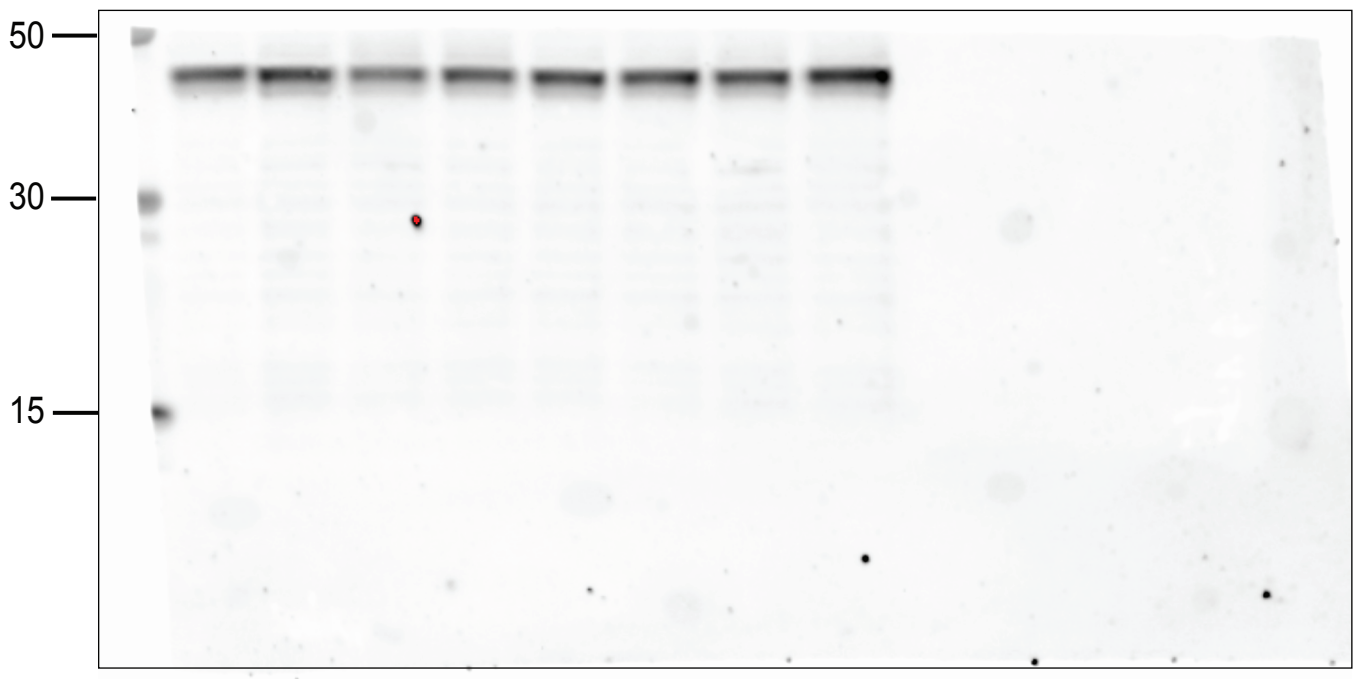

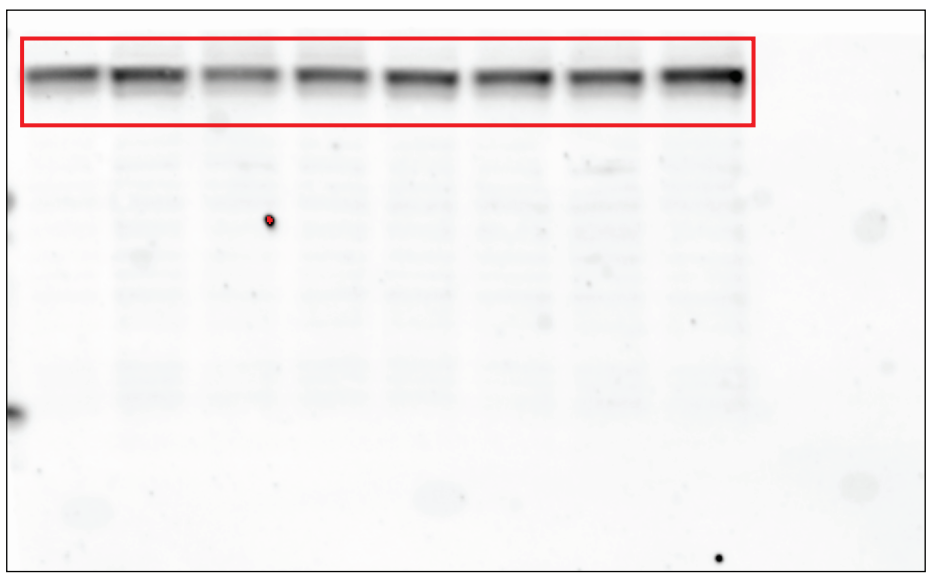

Input: Tubulin

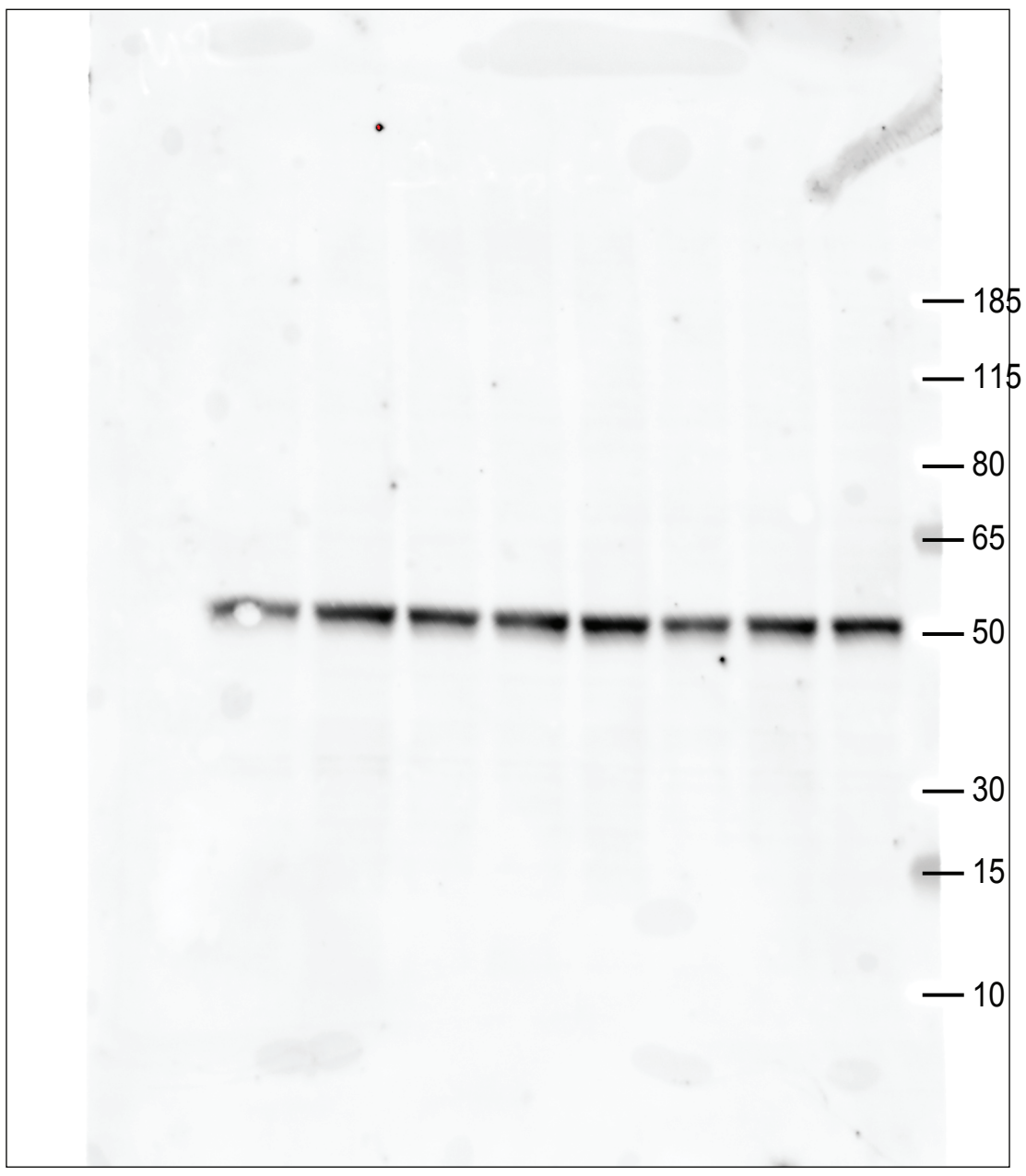

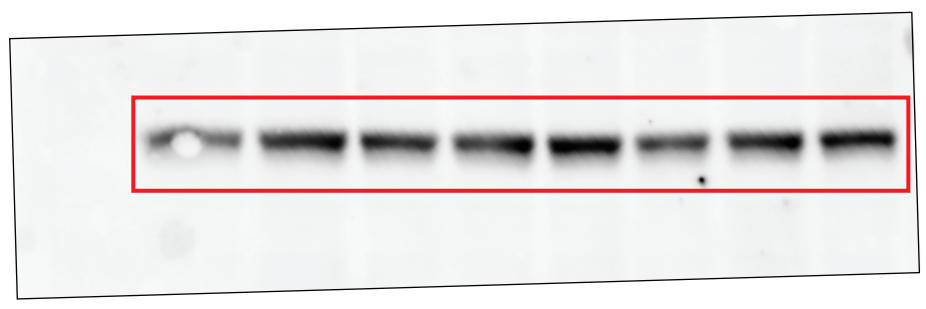

EDF 5c

SMARCA2

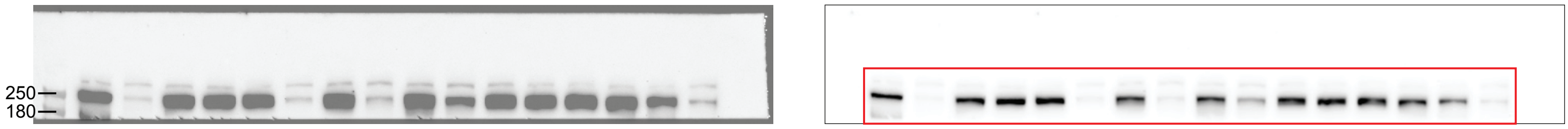

FBXO22

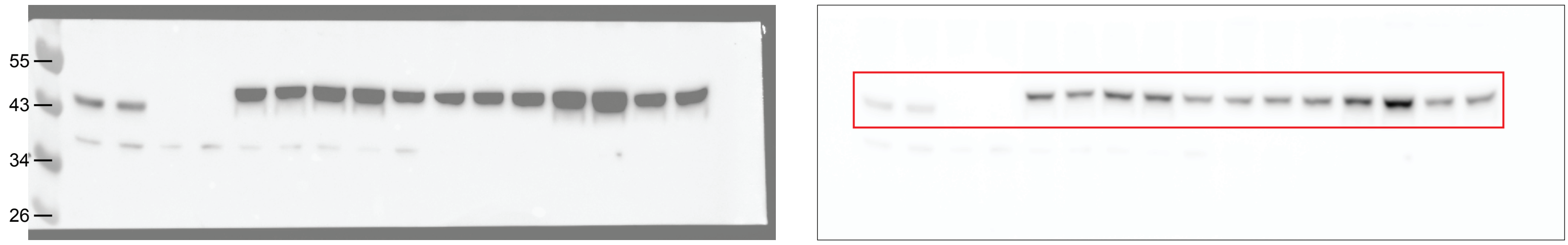

HSP90

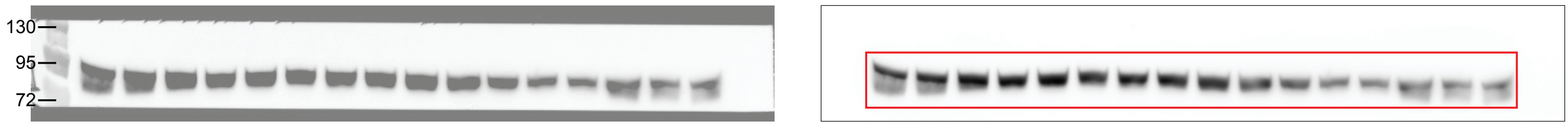

EDF 5d

SMARCA2

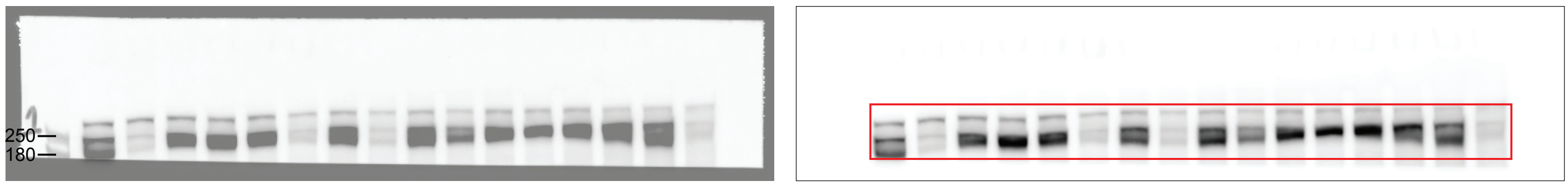

FBXO22

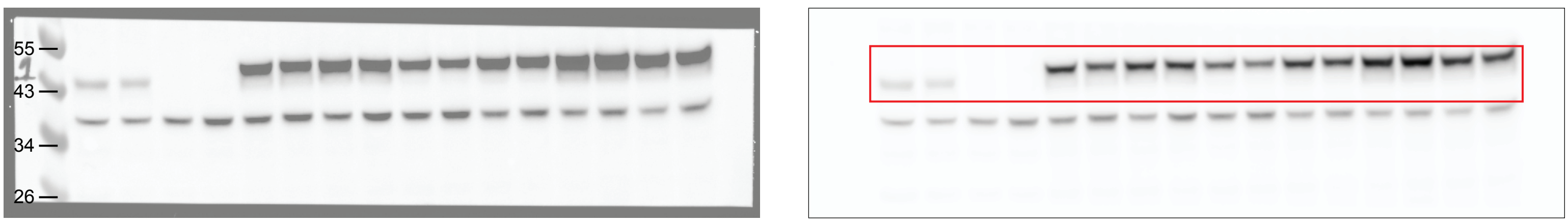

HSP90

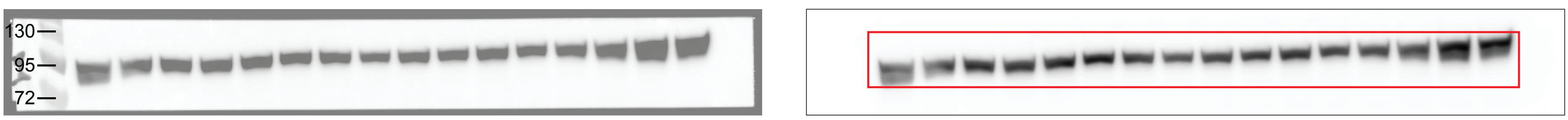

Supplement: Supplementary file 7 [file 41589_2026_2224_MOESM21_ESM.pdf]
