# Supplementary material for: Dual E3 ligase recruitment by monovalent degraders for tunable SMARCA 2/4 degradation
Source: Nat Chem Biol. 2026 May 12;22(10):1666–75. doi: 10.1038/s41589-026-02224-y (PMC13612222; doi:10.1038/s41589-026-02224-y)

EDF 10a

SMARCA2

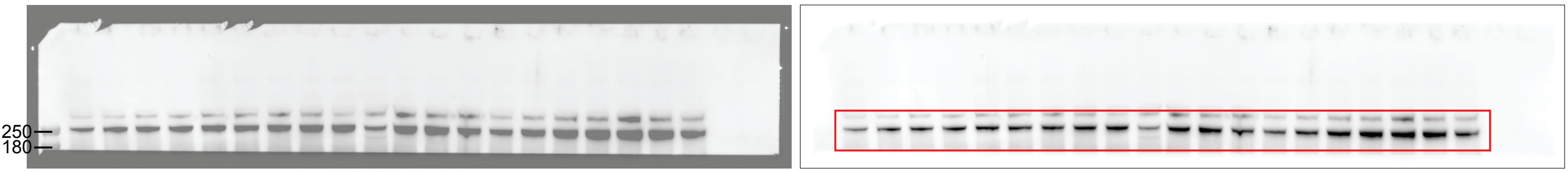

FLAG

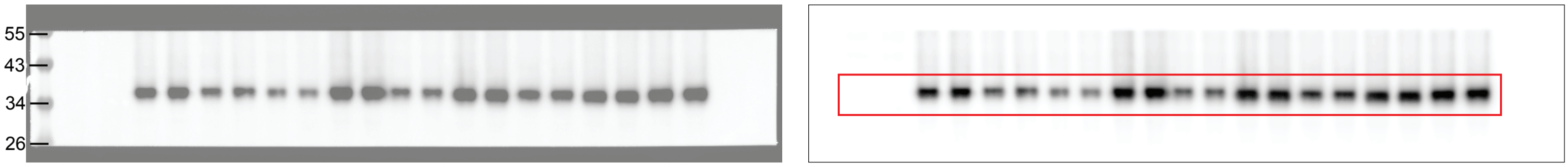

HSP90

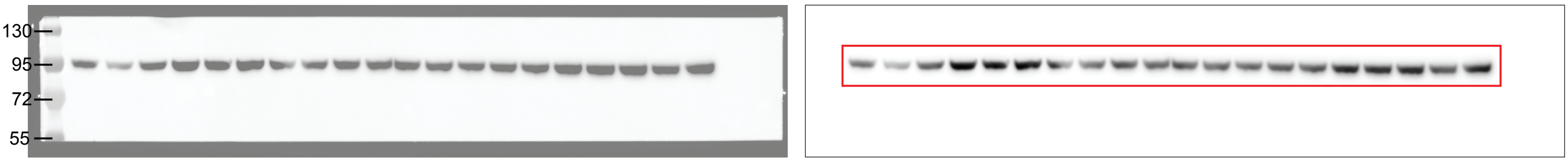

SMARCA2

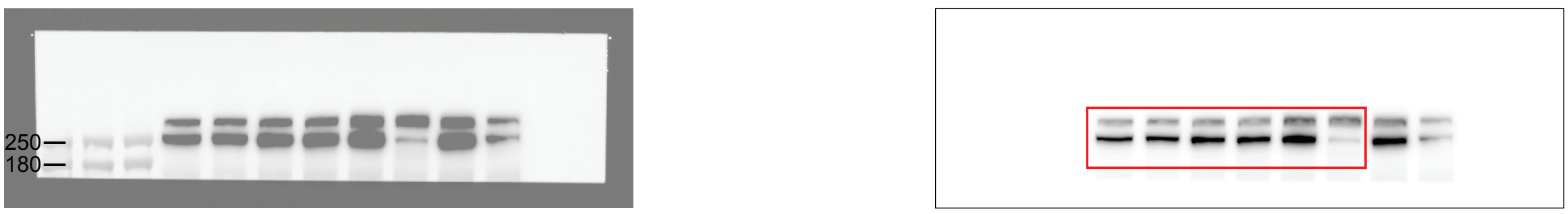

FLAG

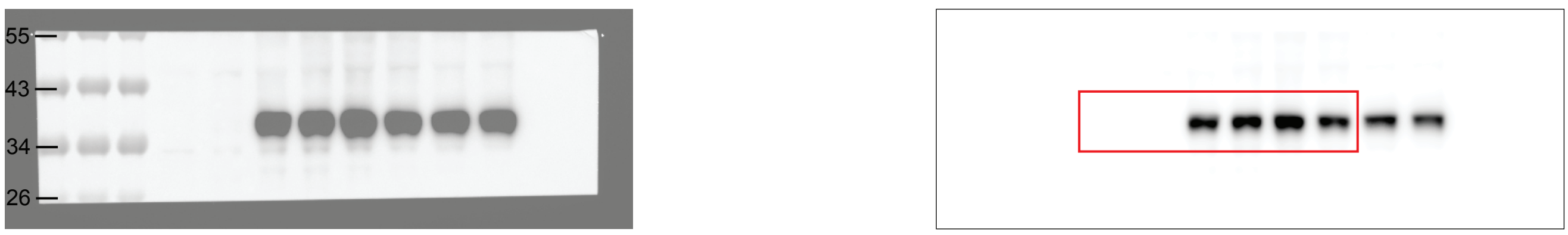

HSP90

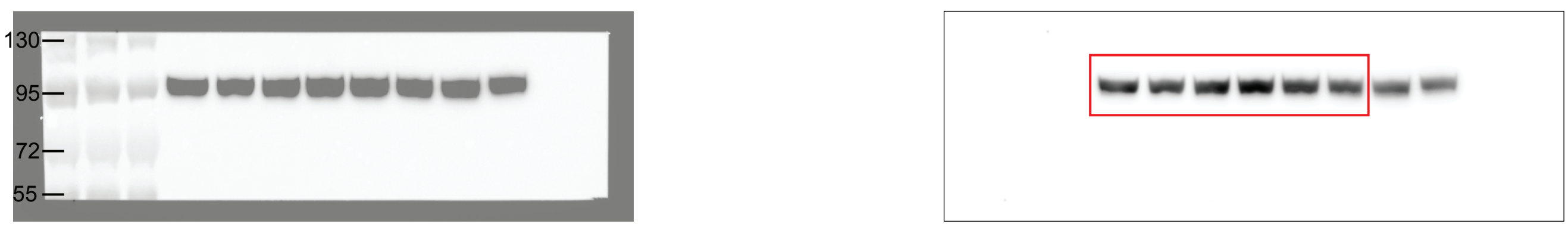

EDF 10b

BRD4

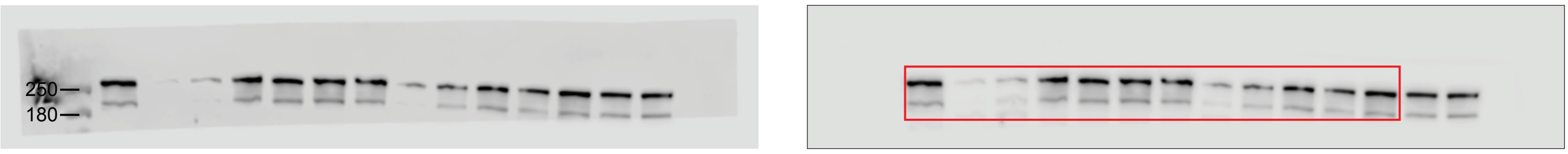

FLAG

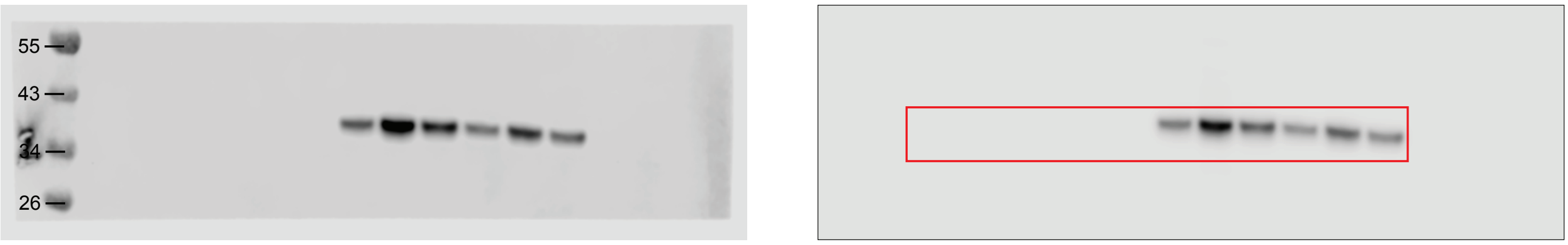

HSP90

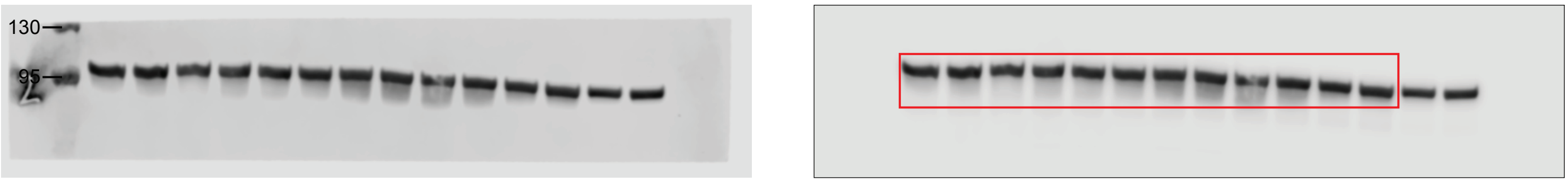

EDF 10c

HA

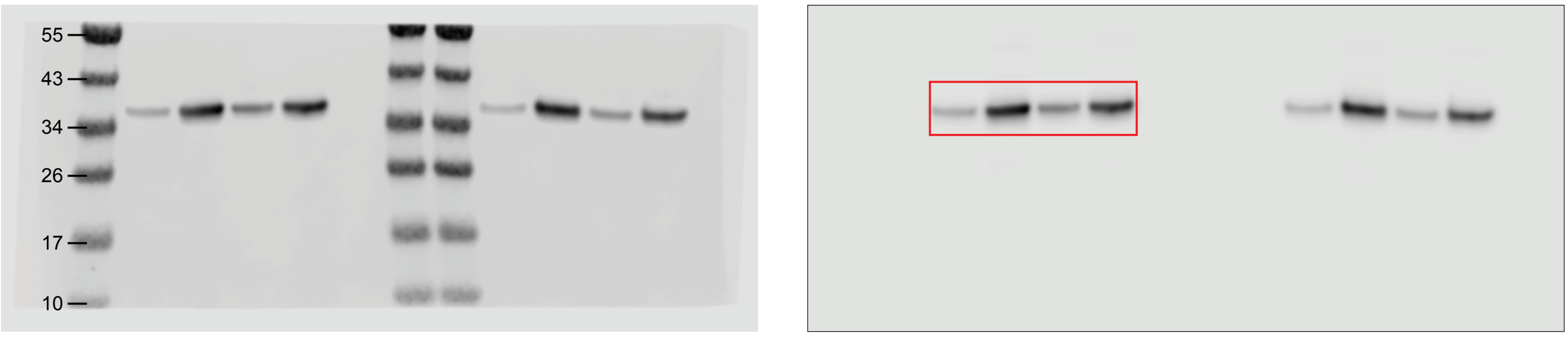

FLAG

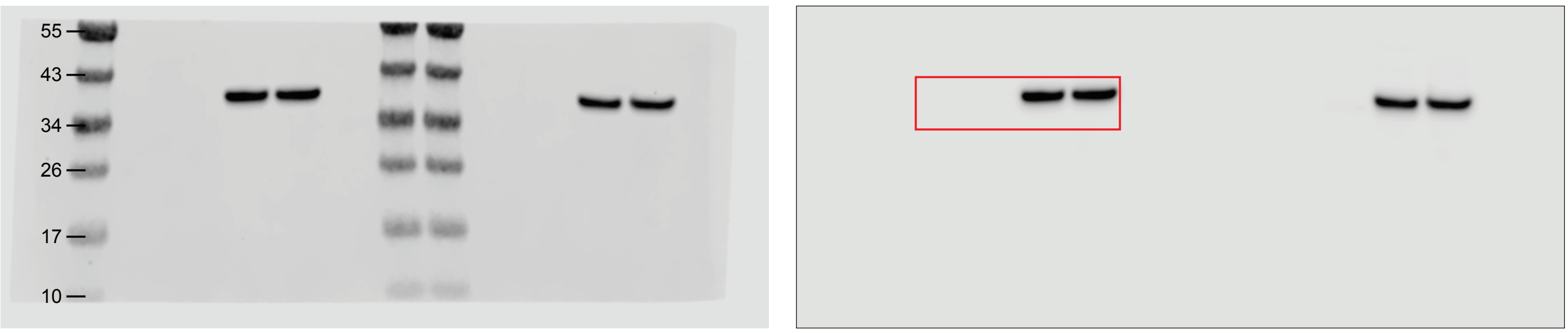

HSP90

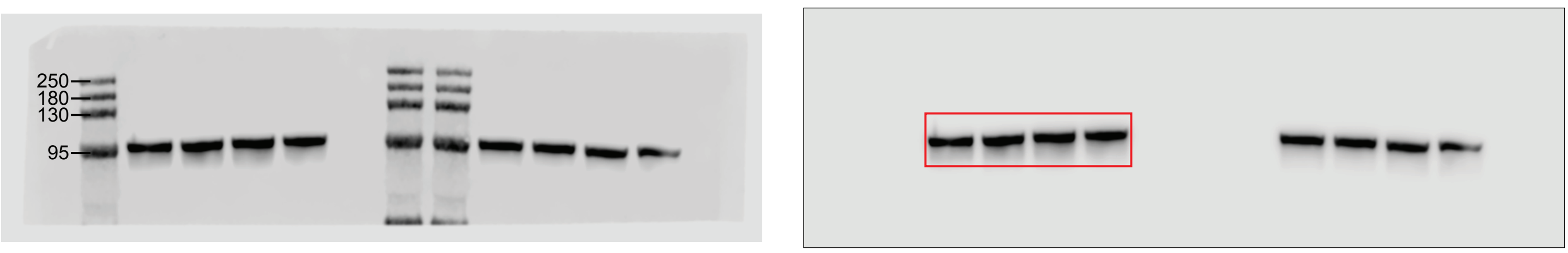

Supplement: Supplementary file 11 [file 41589_2026_2224_MOESM29_ESM.pdf]
