# Supplementary material for: Dual E3 ligase recruitment by monovalent degraders for tunable SMARCA 2/4 degradation
Source: Nat Chem Biol. 2026 May 12;22(10):1666–75. doi: 10.1038/s41589-026-02224-y (PMC13612222; doi:10.1038/s41589-026-02224-y)
Supplement: Supplementary file 12 [file 41589_2026_2224_MOESM2_ESM.pdf]

nature portfolio

Corresponding author(s): Alessio Ciulli, Georg Winter

Last updated by author(s): Mar 5, 2026

Reporting Summary

Nature Portfolio wishes to improve the reproducibility of the work that we publish. This form provides structure for consistency and transparency in reporting. For further information on Nature Portfolio policies, see our Editorial Policies and the Editorial Policy Checklist.

Statistics

For all statistical analyses, confirm that the following items are present in the figure legend, table legend, main text, or Methods section.

n/a	Confirmed	
☐	☒	The exact sample size (*n*) for each experimental group/condition, given as a discrete number and unit of measurement
☐	☒	A statement on whether measurements were taken from distinct samples or whether the same sample was measured repeatedly
☐	☒	The statistical test(s) used AND whether they are one- or two-sided<br>*Only common tests should be described solely by name; describe more complex techniques in the Methods section.*
☒	☐	A description of all covariates tested
☐	☒	A description of any assumptions or corrections, such as tests of normality and adjustment for multiple comparisons
☐	☒	A full description of the statistical parameters including central tendency (e.g. means) or other basic estimates (e.g. regression coefficient) AND variation (e.g. standard deviation) or associated estimates of uncertainty (e.g. confidence intervals)
☐	☒	For null hypothesis testing, the test statistic (e.g. *F*, *t*, *r*) with confidence intervals, effect sizes, degrees of freedom and *P* value noted<br>*Give P values as exact values whenever suitable.*
☒	☐	For Bayesian analysis, information on the choice of priors and Markov chain Monte Carlo settings
☒	☐	For hierarchical and complex designs, identification of the appropriate level for tests and full reporting of outcomes
☒	☐	Estimates of effect sizes (e.g. Cohen's *d*, Pearson's *r*), indicating how they were calculated

Our web collection on statistics for biologists contains articles on many of the points above.

Software and code

Policy information about availability of computer code

Data collection	Flow cytometry and sorting: Data was collected on a BD LSR Fortessa using BD FACSDiva software (v9.0), BD FACSAria Fusion using BD FACSDiva software (v8.0.2)<br>Western blotting/SDS gels: ChemiDoc Touch imaging system (BioRad) operated on Image Lab (v2.4.0.03).<br>NGS: NovaSeq 6000 platform (https://www.illumina.com/)<br>TR-FRET, HiBiT and NanoBRET endpoint assays: BMG Labtech PHERAstar (firmware v1.33), Perkin Elmer Victor X3 plate reader (PerkinElmer 2030 software v4.0)<br>Promega kinetic assays: GloMax® Discover System (software v4.0.0, firmware v4.92)<br>Mass spectrometry: Orbitrap Ascend Tribrid mass spectrometer, Orbitrap Astral Mass Spectrometer and UHPLC Agilent 1290 Infinity III High-Throughput System; InfinityLab Pro iQ Series Mass Detect<br>Cryo-EM: Titan Krios (Thermo Fisher) with Falcon4i direct electron detector, operated on EPU (v3.0) software.
Data analysis	Flow Cytometry Analysis: Flowjo (v10.10.0)<br>FACS-based CRISPR screens: All relevant software is described in detail in the corresponding methods section. Pipelines for sgRNA quantification and statistical analysis are available on Github (https://github.com/ZuberLab/crispr-process-nf/ and https://github.com/ZuberLab/crispr-mageck-nf/). Used packages: cutadapt (v4.4), Bowtie2 (v2.4.5), featureCounts (v2.0.1), MAGeCK (v0.5.9).<br>Deep mutational scanning screen: All relevant software is described in detail in the corresponding methods section. Used packages: samtools (v1.15.1), bedtools (v.2.30.0), Trim Galore (v0.6.6), bwa software package (v0.7.17), AnalyzeSaturationMutagenesis tool from GATK (v4.1.8.1), pheatmap (v.1.0.12).<br>Data compiling, processing and statistical analyses: Microsoft Excel for Microsoft 365 (v2208.16.0.15601.20526), R Studio (v2022.12.0 Build 353) with R (v4.1.0), GraphPad Prism (v9.5.1, v10.0.3, v10.4.1)<br>Mass Spectrometry: DIA-NN, version 1.8 (https://github.com/vdemichev/DIaNN), Agilent OpenLab CDS (version 2.8), Perseus (version

2.0.11.0)
Cryo-EM: Cryosparc (v4.5), eLBOW (in Phenix v1.21-5207), ColabFold (v1.3), Coot (v. 0.9.8.93), Phenix (v1.21-5207), ChimeraX (v1.8), The PyMOL Molecular Graphics System (v3.0.2)
Molecular dynamics simulations: Schrodinger Suite (2024-3 release), Masetro (14.1.138), Desmond (in Schrodinger release).
FBXO22 Structural model outputs: mit Boltz-2 (2025 NVIDIA Corporation), Desmond (in Schrodinger release), Schrodinger Suite (2024-3 release) and PyMOL Molecular Graphics System (v3.0.2)

For manuscripts utilizing custom algorithms or software that are central to the research but not yet described in published literature, software must be made available to editors and reviewers. We strongly encourage code deposition in a community repository (e.g. GitHub). See the Nature Portfolio guidelines for submitting code & software for further information.

Data

Policy information about availability of data

All manuscripts must include a data availability statement. This statement should provide the following information, where applicable:
- Accession codes, unique identifiers, or web links for publicly available datasets
- A description of any restrictions on data availability
- For clinical datasets or third party data, please ensure that the statement adheres to our policy

Source data for Figure 1b, d, Figure 2a, Figure 3, Figure 4b, c, Fig 5a, Extended Data Figure 2c, Extended Data Figure 3a, b, Extended Data Figure 6e, and Extended Data Figure 9a, b are included in the supplementary information files of the manuscript (Supplementary Tables 1-4). Cryo-EM density maps are deposited in the EMDB with the accession code EMD-54549. The atomic model is deposited under Protein Data Bank ID 9S3R, extended PDB ID pdb_00009S3R. Quantitative proteomics data have been deposited to the ProteomeXchange Consortium PRIDE repository with the accession codes PXD065445 and PXD065523.
All biological materials are available upon reasonable requests under material transfer agreements (MTA) with The Centre for Targeted Protein Degradation, University of Dundee, or CeMM Research Center for Molecular Medicine of the Austrian Academy of Sciences, respectively.

Research involving human participants, their data, or biological material

Policy information about studies with human participants or human data. See also policy information about sex, gender (identity/presentation), and sexual orientation and race, ethnicity and racism.

Reporting on sex and gender	N/A
Reporting on race, ethnicity, or other socially relevant groupings	N/A
Population characteristics	N/A
Recruitment	N/A
Ethics oversight	N/A

Note that full information on the approval of the study protocol must also be provided in the manuscript.

Field-specific reporting

Please select the one below that is the best fit for your research. If you are not sure, read the appropriate sections before making your selection.

☒ Life sciences ☐ Behavioural & social sciences ☐ Ecological, evolutionary & environmental sciences

For a reference copy of the document with all sections, see nature.com/documents/nr-reporting-summary-flat.pdf

Life sciences study design

All studies must disclose on these points even when the disclosure is negative.

Sample size	Sample sizes were not predetermined using statistical analyses. Sample sizes were based on prior experience in the field and our previous studies (Zengerle et al. ACS Chem Biol 2015; Gadd et al. Nat Chem Biol 2017; Riching et al. ACS Chem Biol. 2018; Hsia et al. Nature 2024.)
Data exclusions	In quantitative proteomics, BioID, and CRISPR screens, proteins/genes with a p-value and Fold-Change below the cutoff indicated in the figure legend were excluded from data interpretation. For cryo-EM, data filtering is outlined in Supplementary Figure 1 and 2.
Replication	Unless stated in figure legends or method sections, all experiments were done at least twice and the reproductions were successful. The number of independent replicates are specified in the respective figure legends.
Randomization	Since no animal and behavioral studies were performed, there was no need for data randomization. All experiments were carried out with appropriate internal negative and/or positive controls.
Blinding	No blinding was performed as no subjective measurements were done.

Reporting for specific materials, systems and methods

We require information from authors about some types of materials, experimental systems and methods used in many studies. Here, indicate whether each material, system or method listed is relevant to your study. If you are not sure if a list item applies to your research, read the appropriate section before selecting a response.

Materials & experimental systems

n/a	Involved in the study
☐	☒ Antibodies
☐	☒ Eukaryotic cell lines
☒	☐ Palaeontology and archaeology
☒	☐ Animals and other organisms
☒	☐ Clinical data
☒	☐ Dual use research of concern
☒	☐ Plants

Methods

n/a	Involved in the study
☒	☐ ChIP-seq
☐	☒ Flow cytometry
☒	☐ MRI-based neuroimaging

Antibodies

Antibodies used

The following primary antibodies were used for immunoblotting: HSP90 (1:1000, Cell signalling Technology, 4877), FBXO22 (1:200, Santa Cruz, sc-100736), SMARCA2 (1:1000, Bethyl Laboratories, A301-015A), SMARCA4 (1:1000, Bethyl Laboratories, A300-813A), HA epitope (3F10) (1:1000, Merck, 11867423001), HA epitope [C29F4] (1:1000, Cell Signalling Technology, 3724), HiBiT (1:1000, Promega, N7200), IRDye®800CWStreptavidin (1:1000, Licor, 926-32230), hFABTM rhodamine anti-tubulin (1:5000, Bio-Rad, 12004165) and FLAG (1:1000, Sigma-Aldrich, F1804-200UG).

The following secondary antibodies were used: HRP anti-rabbit IgG (1:5000, Cell Signaling Technology, 7074), HRP anti-mouse IgG (1:5000, Cell Signaling Technology, 7076), IRDye 800CW anti-rabbit (1:5000, Licor, 926-32211), IRDye 800CW anti-mouse (1:5000, Licor, 926-32210) and IRDye 800CW anti-rat (1:5000, Licor, 926-32219) and HRP anti-mouse IgG (1:5000, Cell Signaling Technology, 7076).

The following antibodies were used for Immunoprecipitation: HA antibody: 4 µg/500 of µg of cell lysate (51064-2-AP, Proteintech)

The following antibodies were used for FACS analysis and cell sorting: anti-Thy1.1-APC (1:400, BioLegend, 202526), Zombie NIR™ Fixable Viability Dye (1:1000, BioLegend, 423105), and Human TruStain FcX™ Fc Receptor Blocking Solution (1:400, BioLegend, 422301)

Validation

Specificity for the following antibodies were previously confirmed by the Winter group: anti-FBXO22 (Santa Cruz, sc-100736): Knockout validated, disappearance of the band in immunoblotting after expression of sgRNA against FBXO22 (Kagiou et al. Nat Commun 2024). Target specificity for SMARCA2 (Bethyl Laboratories, A301-015A) was validated by manufacturer via protein pulldown and by the Winter group via disappearance of the band in immunoblotting after treatment with degraders/PROTACs. Target specificity for SMARCA4 (Bethyl Laboratories, A300-813A) was confirmed in previous work via CRISPR knockout (Yibo Xue, Nat. Comms. 2019) and by the Winter group via disappearance of the band in immunoblotting after treatment with degraders/PROTACs. Target specificity for HA epitope (Merck, 11867423001), HA epitope (Cell Signalling Technology, 3724), HiBiT (Promega, N7200) and FLAG (Sigma-Aldrich, F1804-200UG) were verified by ectopic overexpression. Target specificity for IRDye®800CWStreptavidin (Licor, 926-32230) was confirmed by biotin treatment (Fig. 1e).

Target specificity for APC anti-mouse CD90.1/Thy-1.1 antibody (BioLegend, 202526) was verified by ectopic overexpression.

Eukaryotic cell lines

Policy information about cell lines and Sex and Gender in Research

Cell line source(s)

Human HEK293/HEK293T and HCT-116 cells were obtained from ATCC.
KBM7 (originally obtained from Haplogen Bioscience) iCas9 cells were a gift from Johannes Zuber (IMP - Research Institute of Molecular Pathology).
Lenti-X 293T cells were purchased from Clontech.
Flp-In T-REx-293 were purchased from Thermo Fisher Scientific.

Authentication

All used cell lines were authenticated by short tandem repeat (STR) profiling. Successful CRISPR-based editing of Knock-in cell lines was confirmed via genotyping and via a lytic HiBiT degradation assay with the SMARCA-specific PROTAC, ACBI1. CRISPR-based editing of Knock out cells were confirmed either by cell-based degradation assays (preventing BRD4 degradation by IBG3 for the DCAF16 KO cell lines) or FBXO22 immunobloting for FBXO22 KO cell lines, as well as by MiaSeq Sequencing. Stable miniTurbo-SMARCA2-BD cell lines were tested by Doxycycline induction followed by Biotin treatment validated by Western blotting.

Mycoplasma contamination

All used cell lines were routinely tested and confirmed negative for mycoplasma contamination.

Commonly misidentified lines
(See ICLAC register)

No commonly misidentified cell lines were used.

Plants

Seed stocks

N/A

Novel plant genotypes

N/A

Authentication

N/A

Flow Cytometry

Plots

Confirm that:

☒ The axis labels state the marker and fluorochrome used (e.g. CD4-FITC).

☒ The axis scales are clearly visible. Include numbers along axes only for bottom left plot of group (a 'group' is an analysis of identical markers).

☒ All plots are contour plots with outliers or pseudocolor plots.

☒ A numerical value for number of cells or percentage (with statistics) is provided.

Methodology

Sample preparation

Sample preparation for each experiment is described in detail in the methods section.

To engineer the SMARCA2/BRD4 protein stability reporers, KBM7 iCas9 or HEK293T cells were transduced with lentivirus expressing SFFV-SMARCA2-BD-mTagBFP-P2A-mCherry or BRD4-Tandem-BDs-mTagBFP–P2A–mCherry to generate stable reporter cell lines. mTagBFP/mCherry double positive cells were sorted on a Cytoflex SRT (Beckman Coulter). For evaluation of SMARCA2/BRD4-TagBFP reporter degradation, cells were treated with DMSO or SMARCA2/BRD4 degraders for 24 hours before flow cytometry analysis on an LSRFortessa (BD Biosciences).

For SMARCA2 stability CRISPR screens, library transduced and selected SMARCA2 stability reporter KBM7 iCas9 cells were induced with doxycycline for 3 days and harvested after 24 hours of treatment with DMSO or degraders. Cells were washed with 1xPBS, stained with Zombie NIR™ Fixable Viability Dye (1:1000, BioLegend) and APC anti-mouse CD90.1/Thy-1.1 antibody (1:400, BioLegend) in the presence of Human TruStain FcX™ Fc Receptor Blocking Solution (1:400, BioLegend), and fixed with BD CytoFix Fixation Buffer (BD Biosciences) for 45 minutes at 4 ºC, while protected from light. Cells were then washed with, and stored in FACS buffer (1xPBS containing 5% FBS and 1 mM EDTA) at 4 ºC over night. The next day, cells were strained trough a 35 µm nylon mesh and sorted on a BD FACSAria™ Fusion (BD Biosciences) using a 70 µm nozzle. Aggregates, dead (ZombieNIR positive), Cas9-negative (GFP-negative) and sgRNA library-negative (Thy1.1–APC-negative) cells were excluded, and the remaining cells were sorted based on their SMARCA2-BFP and mCherry levels into SMARCA2_HIGH (Approximately 5% of cells), SMARCA2_LOW (Approximately 5% of cells), and SMARCA2_MID (Approximately 30% of cells) fractions. For each sample, cells corresponding to at least 2000-fold library representation were sorted per replicate.

To validate CRISPR screen screen hits, SMARCA2 reporter cells were lentivirally transduced with an sgRNA expression plasmid (pLenti-U6-sgRNA-IT-EF1αs-Thy1.1-P2A-NeoR) and selected with Neomycin. Cas9 expression was induced with docycycline (0.4 µg ml−1) for 5 days, , followed by 24hours of degrader treatment before flow cytometry analysis on an LSR Fortessa (BD Biosciences). Data analysis was performed in FlowJo v10.10.0. BFP and mCherry mean fluorescence intensity values from Cas9 expressing cells were first normalized by background subtraction of the respective values from reporter negative cells. SMARCA2 abundance was calculated as the ratio of background subtracted BFP to mCherry mean fluorescence intensity and is displayed normalized to DMSO treated cells.

For DCAF16 deep mutation scanning screens, library transduced and selected SMARCA2/BRD4 stability reporter HEK293T cells were harvested after 24 hours of treatment with DMSO or degraders. Cells were washed with 1xPBS, and then fixed with BD CytoFix Fixation Buffer (BD Biosciences) for 40 minutes at 4°C, while protected from light. Cells were then washed with, and stored in FACS buffer (1xPBS containing 5% FBS and 1 mM EDTA) at 4 ºC over night. The next day, cells were strained through a 35 µm nylon mesh and sorted on a BD FACSAria™ Fusion (BD Biosciences) using a 70 µm nozzle. Aggregates, dead, reporter negative (BFP and mCherry negative), and library negative (EGFP negative) cells were excluded from the sort. The remaining cells were sorted based on their SMARCA2 BD-BFP or BRD4-Tandem-BDs-BFP and mCherry levels into low and high fractions (~5 – 15% of cells in each fraction), ensuring a minimum library representation of 1500 x per replicate.

Instrument

All flow cytometric analyses were performed on BD LSRFortessa (4 laser, 16 detector configuration; BD Bioscience). All sorts

Instrument	were performed on BD FACSAria Fusion (5 lasers, 16 detectors; BD Bioscience) or CytoFLEX SRT (4 lasers, 15 detectors; Beckman Coulter) cell sorters.
Software	BD FACSDiva software (v8.0.2 and v9.0), Beckman Coulter CytExpert SRT (v 1.1.0.10007), Flowjo (v10.10.0)
Cell population abundance	In SMARCA2 stability CRISPR screens, cells were sorted into SMARCA2_HIGH (Approximately 5% of cells), SMARCA2_LOW (Approximately 5% of cells), and SMARCA2_MID (Approximately 30% of cells) populations. In DCAF16 deep mutational scanning screens, cells were sorted into SMARCA2/BRD4_LOW (5-15% of cells) and SMARCA2/BRD4_HIGH (5-15% of cells) populations All collected fractions were reanalyzed using a BD LSRFortessa for purity.
Gating strategy	In all analyses, forward scatter area vs. side scatter area plot was used to separate cell events from debris and dead cells. Forward scatter height vs. forward scatter area and/or side scatter width vs. side scatter height plots were used to separate single cells from aggregates.<br><br>For the sorting of fixed cells in CRISPR SMARCA2 protein stability screens, dead cells were excluded based on Zombia-NIR staining (BV786-A) vs FSC-A. SgRNA library Thy1.1 (APC-A), Cas9-GFP (Alexa Fluor 488-A) and reporter (Texas Red-A) triple positive cells were sorted into SMARCA2_LOW, SMARCA2_HIGH, and SMARCA2_MID populations based on SMARCA2-BFP (BV421-A) vs mCherry (TexasRed-A) scatter plots. These gates were dynamically adjusted to keep the percentage at approximately 5% for SMARCA2_LOW and SMARCA2_HIGH, and approximately 30% for SMARCA2 MID populations.<br><br>For the sorting of fixed cells in DCAF16 deep mutational scanning screens, DCAF16 library (Alexa Fluor 488-A) and reporter (BV421-A + TexasRed-A) positive cells were sorted into SMARCA2/BRD4_LOW and SMARCA2/BRD4_HIGH populations based on SMARCA2/BRD4-BFP (BV421-A) vs mCherry (TexasRed-A) scatter plots. These gates were dynamically adjusted to keep the percentage at approximately 5-15% for SMARCA2/BRD4_LOW and SMARCA2/BRD4_HIGH populations.<br><br>A figure exemplifying the gating strategy for all FACS experiments and FACS-based screens is provided (Supplementary Figure 3.

☒ Tick this box to confirm that a figure exemplifying the gating strategy is provided in the Supplementary Information.
