# Supplementary material for: Dual E3 ligase recruitment by monovalent degraders for tunable SMARCA 2/4 degradation
Source: Nat Chem Biol. 2026 May 12;22(10):1666–75. doi: 10.1038/s41589-026-02224-y (PMC13612222; doi:10.1038/s41589-026-02224-y)
Supplement: Supplementary file 13 [file 41589_2026_2224_MOESM30_ESM.pdf]

EDF10d

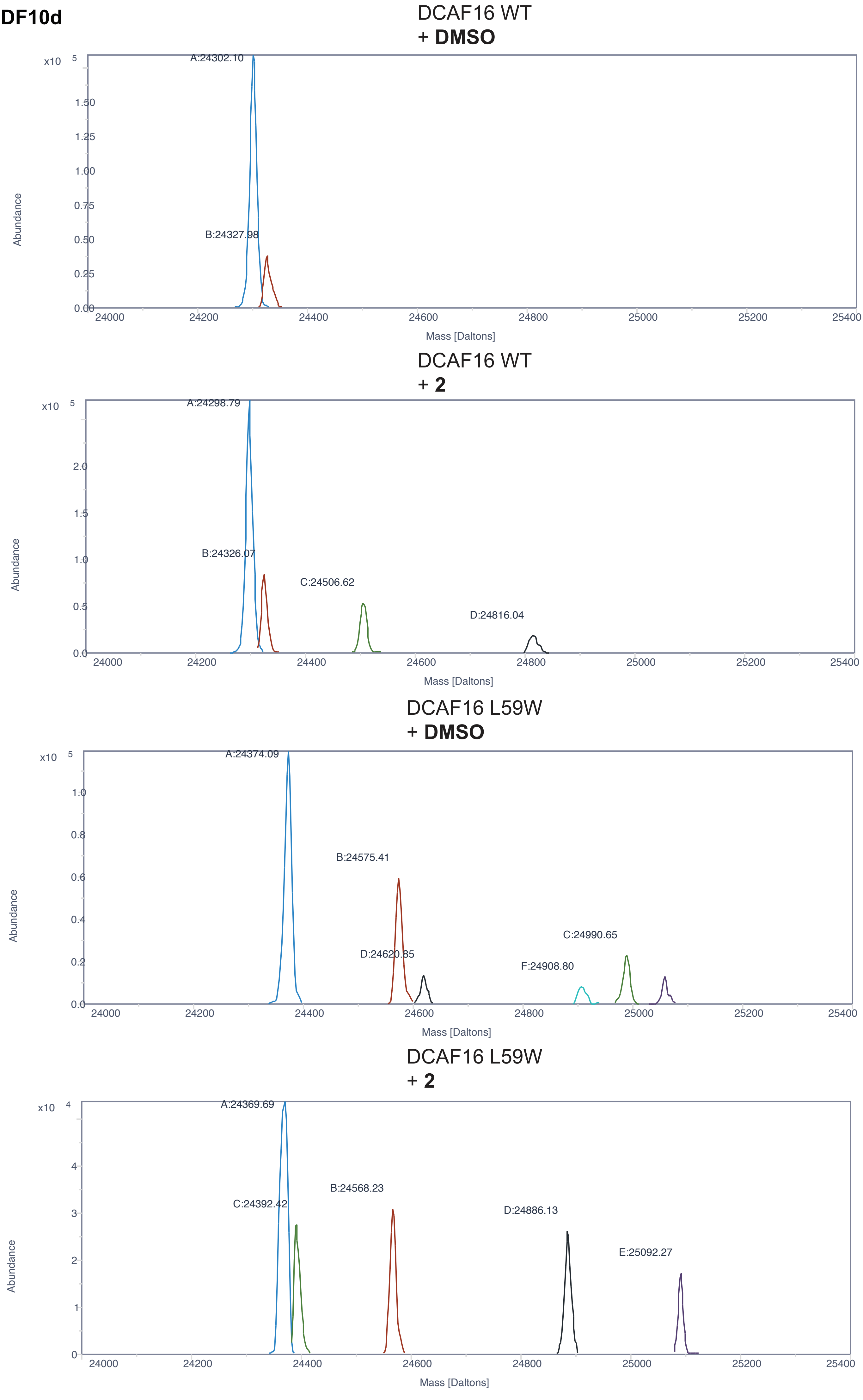
DCAF16 WT
+ DMSO
x10 5
A:24302.10
B:24327.98
Abundance
Mass [Daltons]
DCAF16 WT
+ 2
A:24298.79
B:24326.07
C:24506.62
D:24816.04
DCAF16 L59W
+ DMSO
A:24374.09
B:24575.41
D:24620.85
C:24990.65
F:24908.80
DCAF16 L59W
+ 2
x10 4
A:24369.69
C:24392.42
B:24568.23
D:24886.13
E:25092.27
